# Supplementary material for: Phytochemistry and Comprehensive Chemical Profiling Study of Flavonoids and Phenolic Acids in the Aerial Parts of Allium Mongolicum Regel and Their Intestinal Motility Evaluation
Source: Molecules. 2020 Jan 29;25(3):577. doi: 10.3390/molecules25030577 (PMC7036834; doi:10.3390/molecules25030577)
Supplement: Supplementary file 1 [file molecules-25-00577-s001.pdf]

Phytochemistry and Comprehensive Chemical Profiling Study of Flavonoids and Phenolic Acids in the Aerial Parts of *Allium Mongolicum* Regel and Their Intestinal Motility Evaluation

Yongzhe Dong [1,2,¶], Jingya Ruan [1,2,¶], Zhijuan Ding [2], Wei Zhao [1], Mimi Hao [2], Ying Zhang [2], HongYu Jiang [2], Yi Zhang [1,2,*] and Tao Wang[1,2,*]

1 Tianjin Key Laboratory of TCM Chemistry and Analysis, Tianjin University of Traditional Chinese Medicine, 10 Poyanghu Road, West Area, Tuanbo New Town, Jinghai District,301617,Tianjin, China; dongyongzhe44@hotmail.com (Y.D.); Ruanjy19930919@163.com (J.R.); zhaowei126123@126.com (W.Z.)

2 Institute of TCM, Tianjin University of Traditional Chinese Medicine, 10 Poyanghu Road, West Area, Tuanbo New Town, Jinghai District, 301617, Tianjin, China; 15222792071@163.com (Z.D.); haomimi126@126.com (M.H.); zyingtzy@163.com (Y.Z.); jhy15731602454@163.com (H.J.)

¶ Y. Dong and J. Ruan contributed equally to this work.

* Correspondence: zhwwxzh@tjutcm.edu.cn (Y.Z.); wangtao@tjutcm.edu.cn (T.W.); Tel./Fax: +86-22-5959-6168 (T.W.)

Fig. S1. ^{1}H NMR (500 MHz, DMSO-d_6) spectrum of compound **1** 4
Fig. S2. ^{13}C NMR (125 MHz, DMSO-d_6) spectrum of compound **1**............ 4
Fig. S3. DEPT 135 (DMSO-d_6) spectrum of compound **1**............ 5
Fig. S4. ^{1}H ^{1}H COSY (DMSO-d_6) spectrum of compound **1**............ 5
Fig. S5. HSQC (DMSO-d_6) spectrum of compound **1**............ 6
Fig. S6. HMBC (DMSO-d_6) spectrum of compound **1**............ 6
Fig. S7. HSQC-TOCSY (DMSO-d_6) spectrum of compound **1**............ 7
Fig. S8. ESI-Q-Orbitrap MS spectrum of compound **1**............ 7
Fig. S9. ^{1}H NMR (500 MHz, DMSO-d_6) spectrum of compound **2**............ 8
Fig. S10. ^{13}C NMR (125 MHz, DMSO-d_6) spectrum of compound **2**............ 8
Fig. S11. ^{1}H ^{1}H COSY (DMSO-d_6) spectrum of compound **2**............ 9
Fig. S12. HSQC (DMSO-d_6) spectrum of compound **2**............ 9
Fig. S13. HMBC (DMSO-d_6) spectrum of compound **2**............ 10
Fig. S14. HSQC-TOCSY (DMSO-d_6) spectrum of compound **2**............ 10
Fig. S15. ESI-Q-Orbitrap MS spectrum of compound **2**............ 11
Fig. S16. ^{1}H NMR (500 MHz, DMSO-d_6) spectrum of compound **3**............ 12
Fig. S17. ^{13}C NMR (125 MHz, DMSO-d_6) spectrum of compound **3**............ 12
Fig. S18. DEPT 135 (DMSO-d_6) spectrum of compound **3**............ 13
Fig. S19. ^{1}H ^{1}H COSY (DMSO-d_6) spectrum of compound **3**............ 13
Fig. S20. HSQC (DMSO-d_6) spectrum of compound **3**............ 14
Fig. S21. HMBC (DMSO-d_6) spectrum of compound **3**............ 14
Fig. S22. HSQC-TOCSY (DMSO-d_6) spectrum of compound **3**............ 15
Fig. S23. ESI-Q-Orbitrap MS spectrum of compound **3**............ 15
Fig. S24. ^{1}H NMR (500 MHz, DMSO-d_6) spectrum of compound **4**............ 16
Fig. S25. ^{13}C NMR (125 MHz, DMSO-d_6) spectrum of compound **4**............ 16
Fig. S26. ^{1}H ^{1}H COSY (DMSO-d_6) spectrum of compound **4**............ 17
Fig. S27. HSQC (DMSO-d_6) spectrum of compound **4**............ 17
Fig. S28. HMBC (DMSO-d_6) spectrum of compound **4**............ 18
Fig. S29. ESI-Q-Orbitrap MS spectrum of compound **4**............ 18
Fig. S30. ^{1}H NMR (500 MHz, DMSO-d_6) spectrum of compound **5**............ 19
Fig. S31. ^{13}C NMR (125 MHz, DMSO-d_6) spectrum of compound **5**............ 19
Fig. S32. DEPT 135 (DMSO-d_6) spectrum of compound **5**............ 20
Fig. S33. ^{1}H ^{1}H COSY (DMSO-d_6) spectrum of compound **5**............ 20
Fig. S34. HSQC (DMSO-d_6) spectrum of compound **5**............ 21
Fig. S35. HMBC (DMSO-d_6) spectrum of compound **5**............ 21
Fig. S36. ESI-Q-Orbitrap MS spectrum of compound **5**............ 22
Fig. S37. ^{1}H NMR (500 MHz, DMSO-d_6) spectrum of compound **6**............ 23
Fig. S38. ^{13}C NMR (125 MHz, DMSO-d_6) spectrum of compound **6**............ 23
Fig. S39. DEPT 135 (DMSO-d_6) spectrum of compound **6**............ 24
Fig. S40. ^{1}H ^{1}H COSY (DMSO-d_6) spectrum of compound **6**............ 24
Fig. S41. HSQC (DMSO-d_6) spectrum of compound **6**............ 25
Fig. S42. HMBC (DMSO-d_6) spectrum of compound **6**............ 25
Fig. S43. HSQC-TOCSY (DMSO-d_6) spectrum of compound **6**............ 26
Fig. S44. ESI-Q-Orbitrap MS spectrum of compound **6**............ 26

Fig. S45. ^{1}H NMR (500 MHz, DMSO-d_6) spectrum of compound **7**.......... 27
Fig. S46. ^{13}C NMR (125 MHz, DMSO-d_6) spectrum of compound **7**.......... 27
Fig. S47. DEPT 135 (DMSO-d_6) spectrum of compound **7**.......... 28
Fig. S48. ^{1}H ^{1}H COSY (DMSO-d_6) spectrum of compound **7**.......... 28
Fig. S49. HSQC (DMSO-d_6) spectrum of compound **7**.......... 29
Fig. S50. HMBC (DMSO-d_6) spectrum of compound **7**.......... 29
Fig. S51. ESI-Q-Orbitrap MS spectrum of compound **7**.......... 30
Fig. S52. ^{1}H NMR (500 MHz, DMSO-d_6) spectrum of compound **8**.......... 31
Fig. S53. ^{13}C NMR (125 MHz, DMSO-d_6) spectrum of compound **8**.......... 31
Fig. S54. DEPT 135 (DMSO-d_6) spectrum of compound **8**.......... 32
Fig. S55. ^{1}H ^{1}H COSY (DMSO-d_6) spectrum of compound **8**.......... 32
Fig. S56. HSQC (DMSO-d_6) spectrum of compound **8**.......... 33
Fig. S57. HMBC (DMSO-d_6) spectrum of compound **8**.......... 33
Fig. S58. ESI-Q-Orbitrap MS spectrum of compound **8**.......... 34
Fig. S59. ^{1}H NMR (500 MHz, DMSO-d_6) spectrum of compound **9**.......... 35
Fig. S60. ^{13}C NMR (125 MHz, DMSO-d_6) spectrum of compound **9**.......... 35
Fig. S61. DEPT 135 (DMSO-d_6) spectrum of compound **9**.......... 36
Fig. S62. ^{1}H ^{1}H COSY (DMSO-d_6) spectrum of compound **9**.......... 36
Fig. S63. HSQC (DMSO-d_6) spectrum of compound **9**.......... 37
Fig. S64. HMBC (DMSO-d_6) spectrum of compound **9**.......... 37
Fig. S65. HSQC-TOCSY (DMSO-d_6) spectrum of compound **9**.......... 38
Fig. S66. ESI-Q-Orbitrap MS spectrum of compound **9**.......... 38
Fig. S67. ^{1}H NMR (500 MHz, CD_3OD) spectrum of compound **10**.......... 39
Fig. S68. ^{13}C NMR (125 MHz, CD_3OD) spectrum of compound **10**.......... 39
Fig. S69. DEPT 135 (CD_3OD) spectrum of compound **10**.......... 40
Fig. S70. ^{1}H ^{1}H COSY (CD_3OD) spectrum of compound **10**.......... 40
Fig. S71. HSQC (CD_3OD) spectrum of compound **10**.......... 41
Fig. S72. HMBC (CD_3OD) spectrum of compound **10**.......... 41
Fig. S73. ESI-Q-Orbitrap MS spectrum of compound **10**.......... 42
Fig. S74. The tandem MS of the $[M{-}H]^-$ ions for peaks 30' and 33'.......... 43
Fig. S75. The tandem MS of the [M−H]− ions for peak 28'.......... 44
Fig. S76. The MS/MS spectrum of $[M{-}H]^-$ ions of fragment ions for peaks 7', 12' and 17' by ESI-Q-Orbitrap MS.......... 45
Extraction and Isolation 46
Table S1. The qualitative analysis of *A. mongolicum* extracts by UPLC-ESI-Q-Orbitrap MS.......... 50

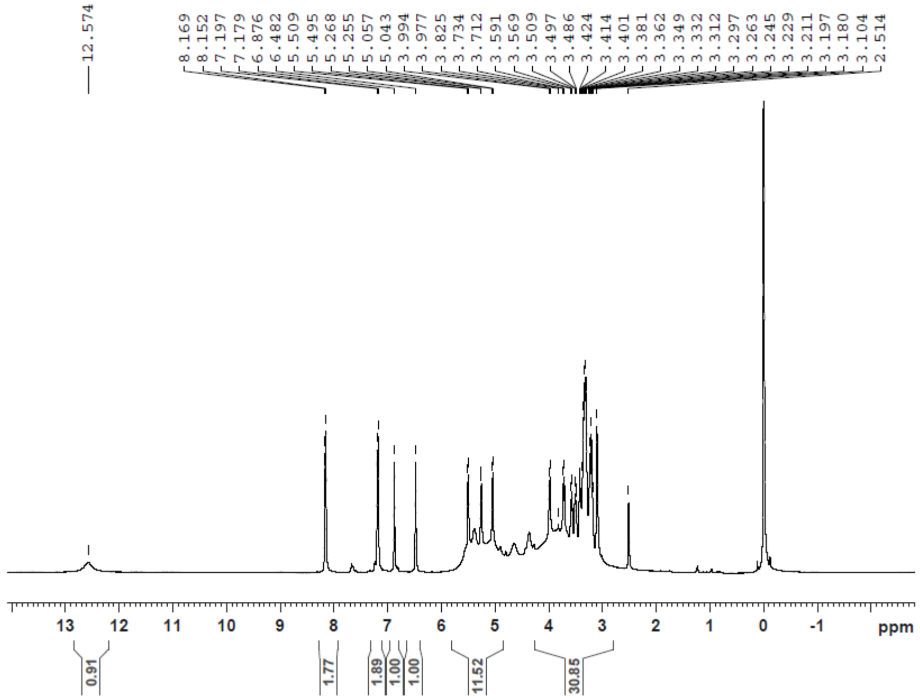

Fig. S1. ^{1}H NMR (500 MHz, DMSO-d_6) spectrum of compound **1**

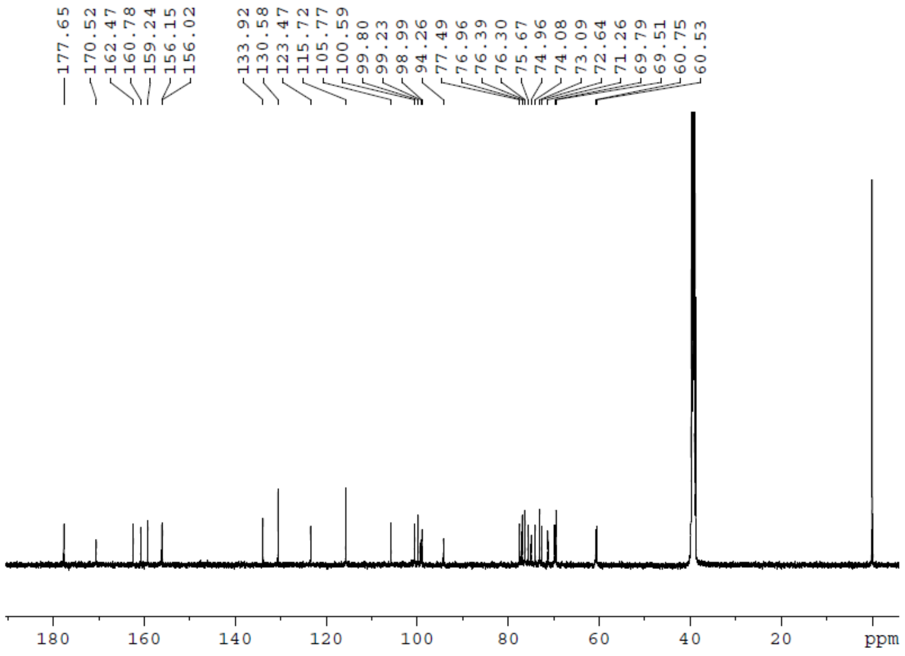

Fig. S2. ^{13}C NMR (125 MHz, DMSO-d_6) spectrum of compound **1**

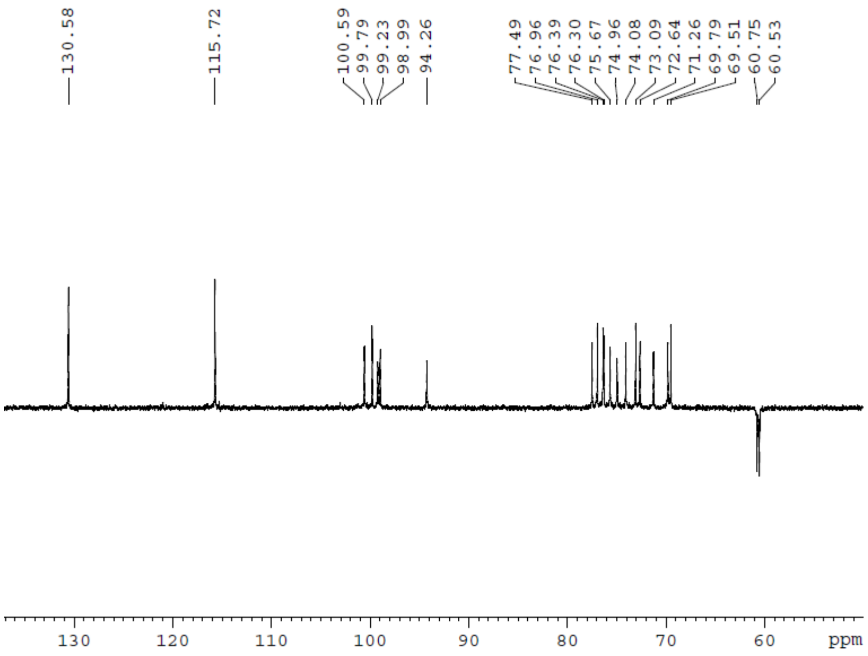

Fig. S3. DEPT 135 (DMSO-d_6) spectrum of compound **1**

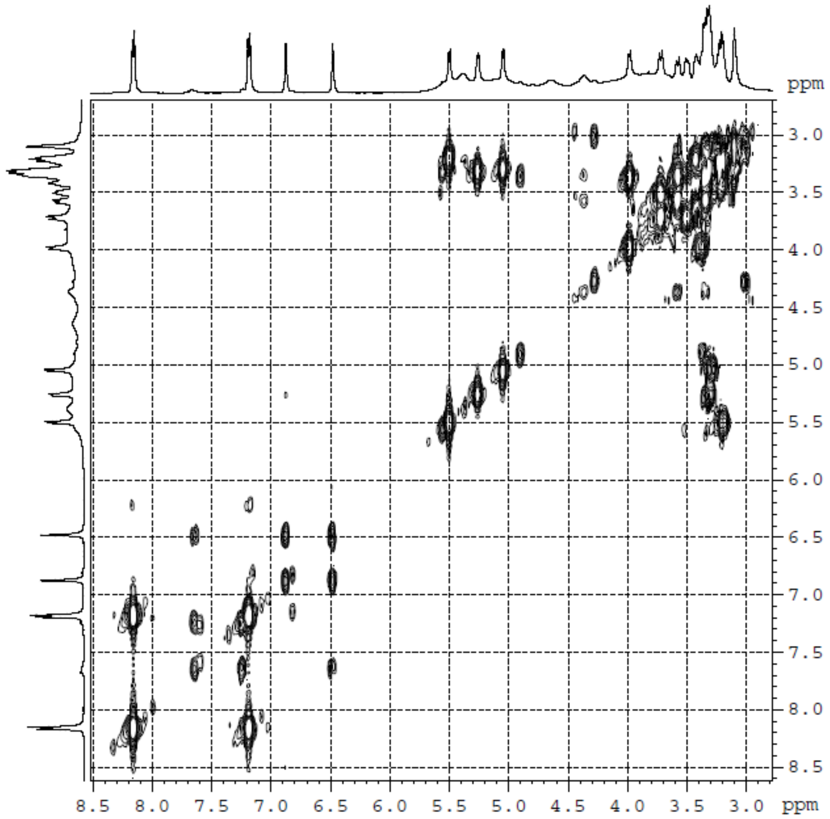

Fig. S4. ^{1}H ^{1}H COSY (DMSO-d_6) spectrum of compound **1**

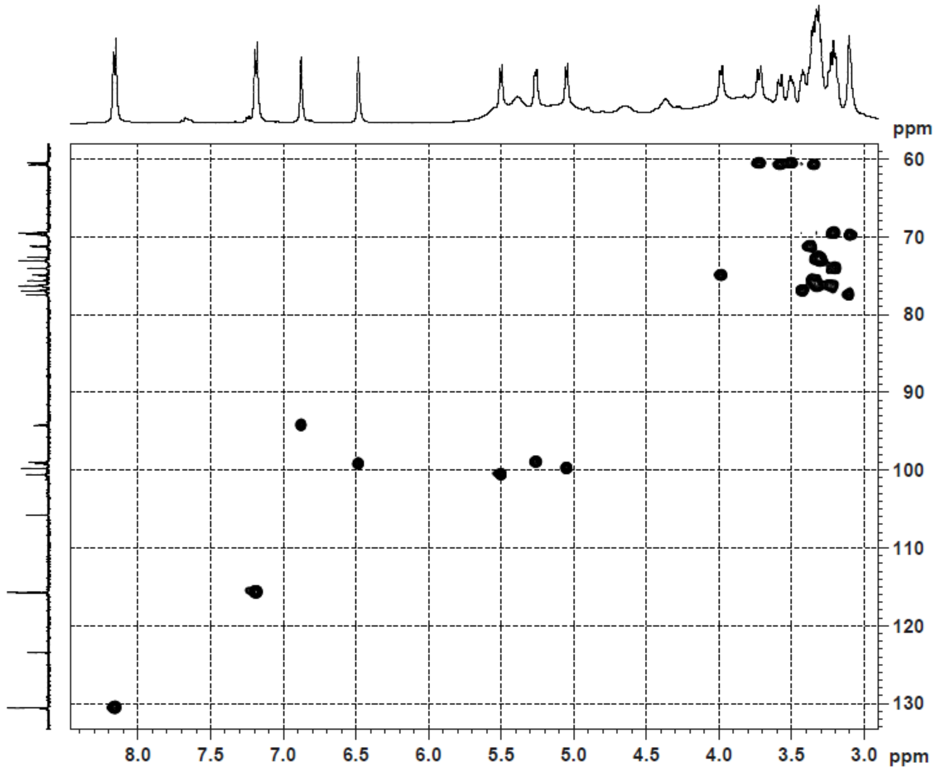

Fig. S5. HSQC (DMSO-d_6) spectrum of compound **1**

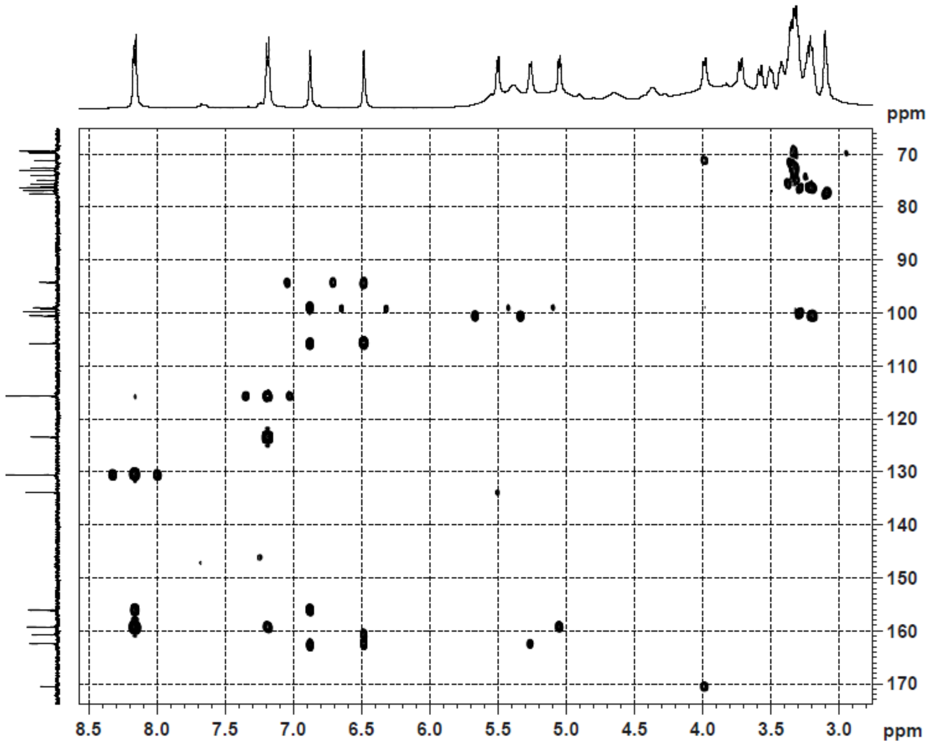

Fig. S6. HMBC (DMSO-d_6) spectrum of compound **1**

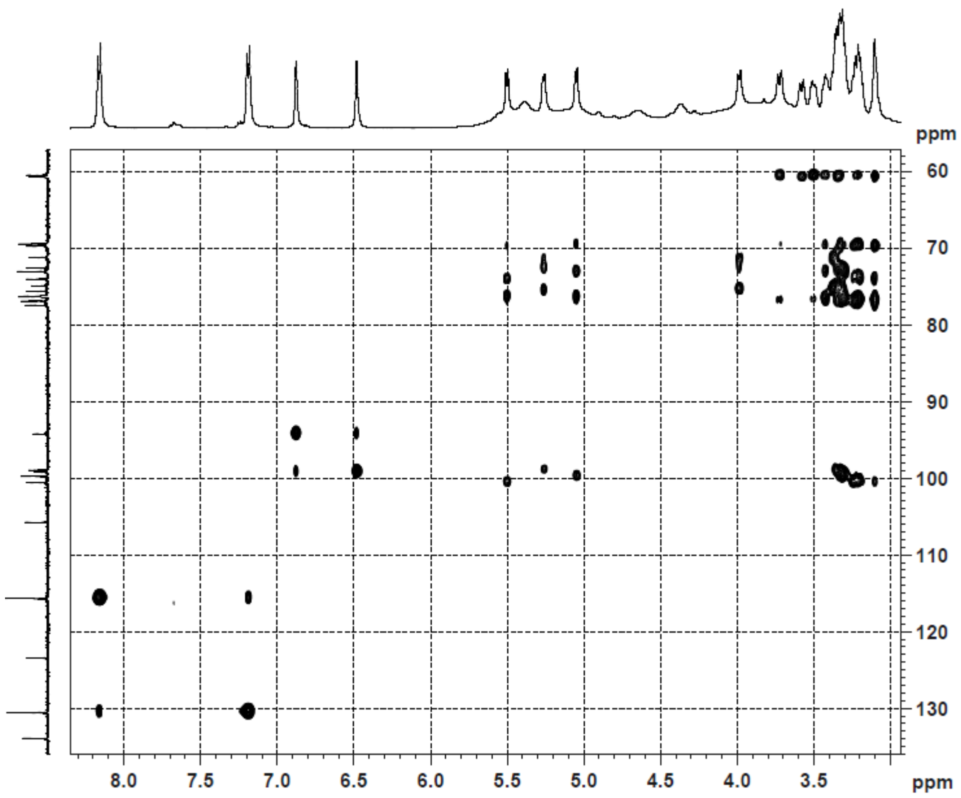

Fig. S7. HSQC-TOCSY (DMSO-d_6) spectrum of compound **1**

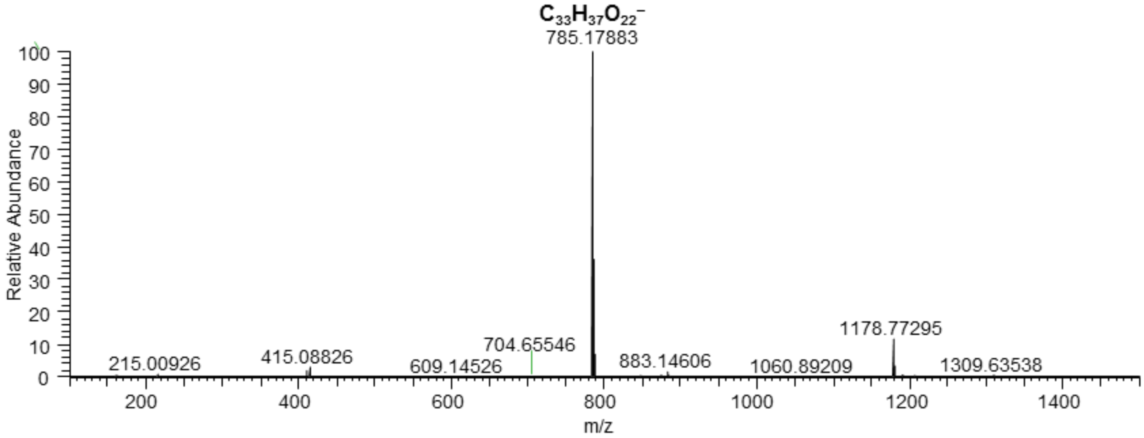

Fig. S8. ESI-Q-Orbitrap MS spectrum of compound **1**

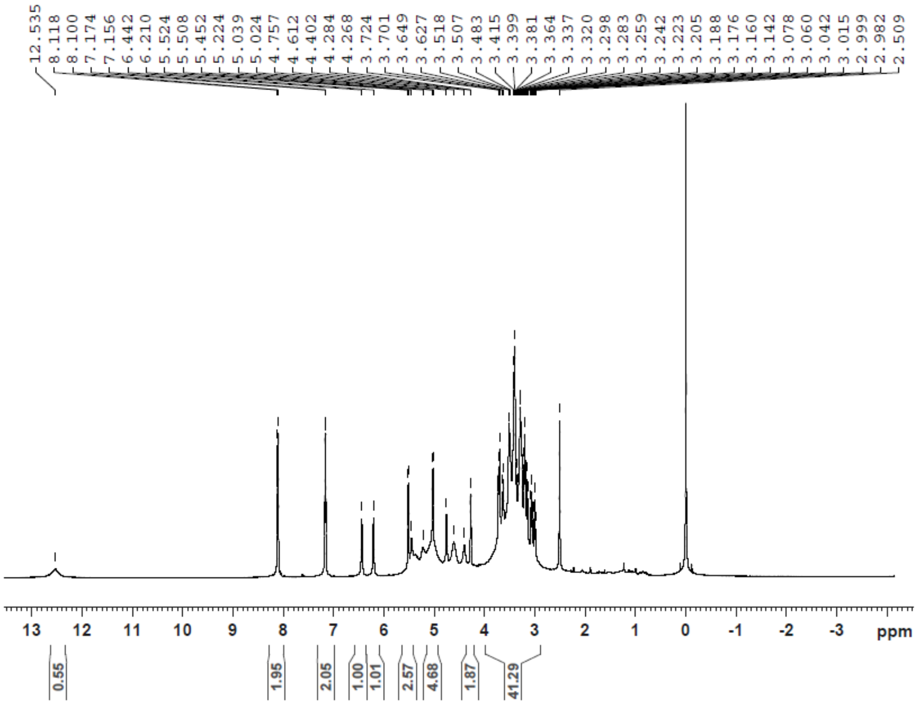

Fig. S9. ^{1}H NMR (500 MHz, DMSO-d_6) spectrum of compound **2**

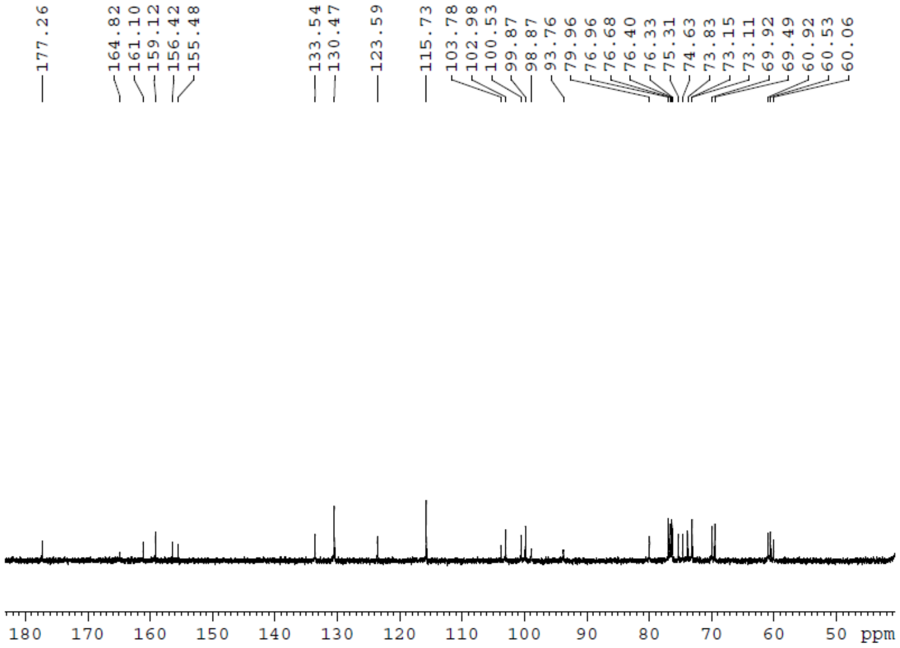

Fig. S10. ^{13}C NMR (125 MHz, DMSO-d_6) spectrum of compound **2**

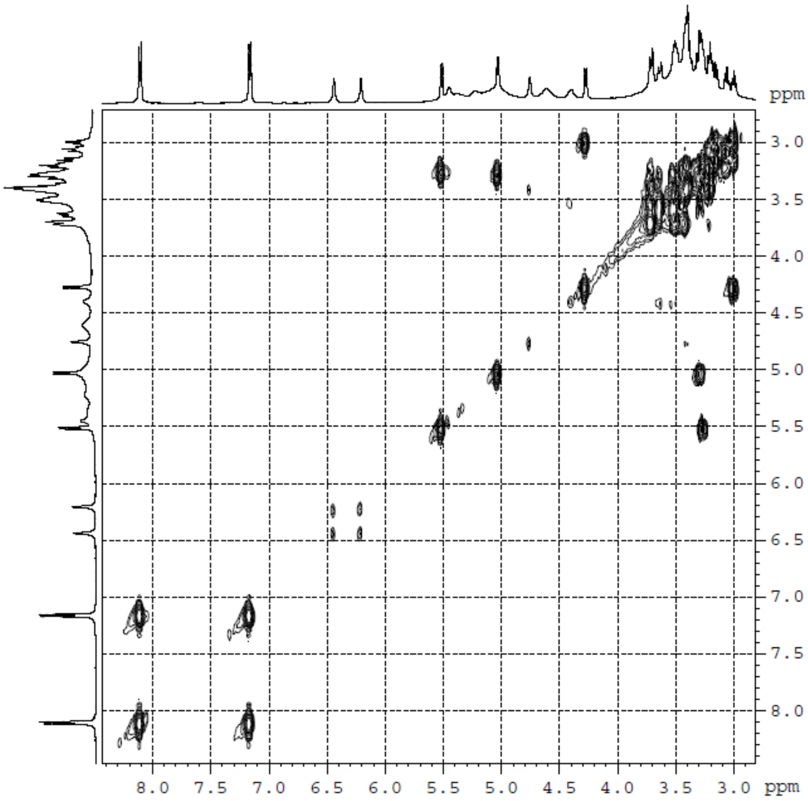

Fig. S11. ^{1}H ^{1}H COSY (DMSO-d_6) spectrum of compound **2**

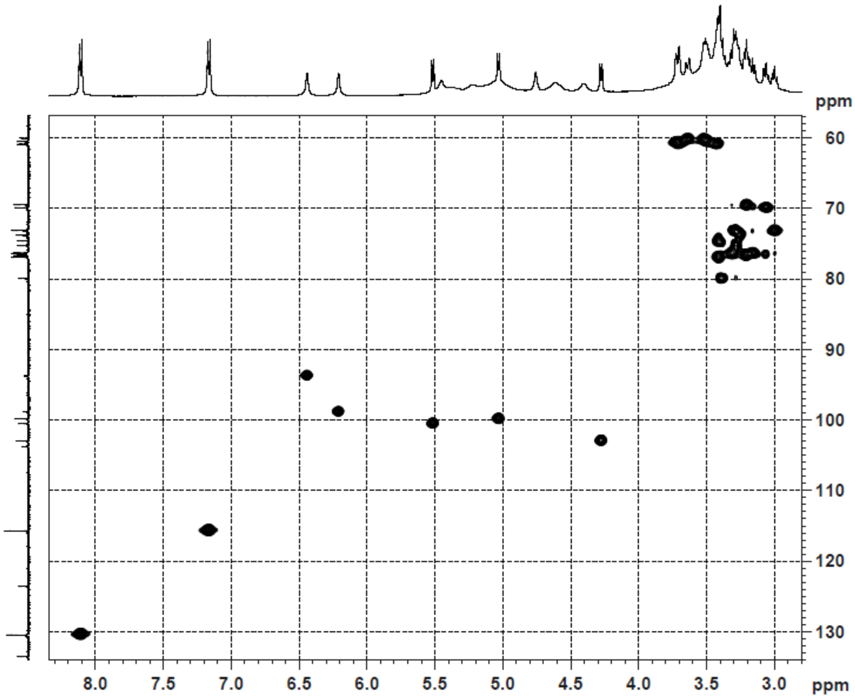

Fig. S12. HSQC (DMSO-d_6) spectrum of compound **2**

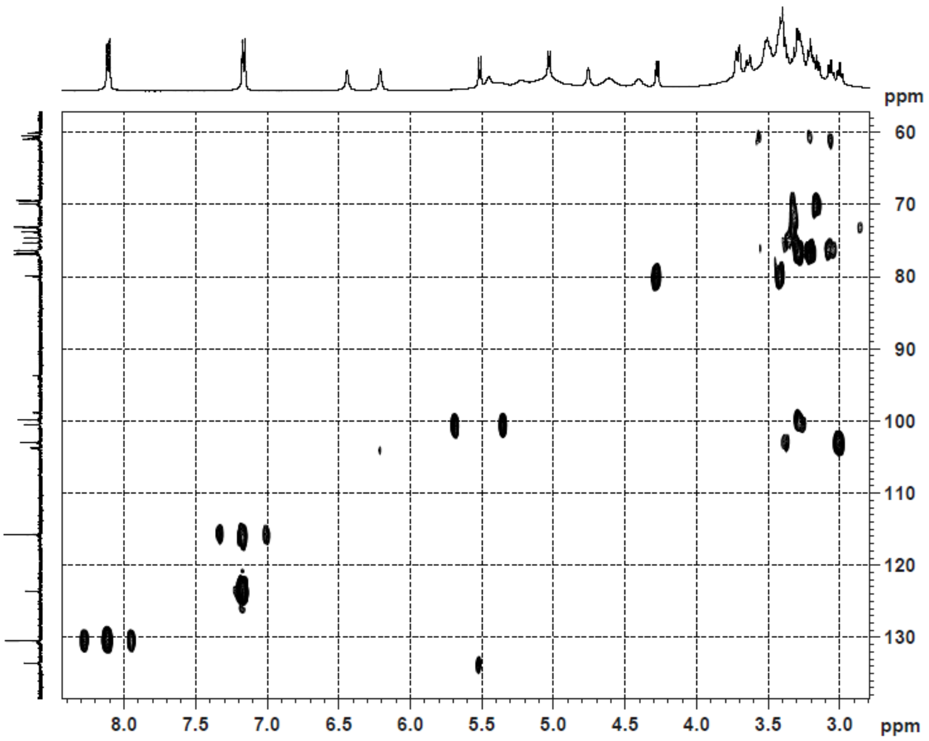

Fig. S13. HMBC (DMSO-d_6) spectrum of compound **2**

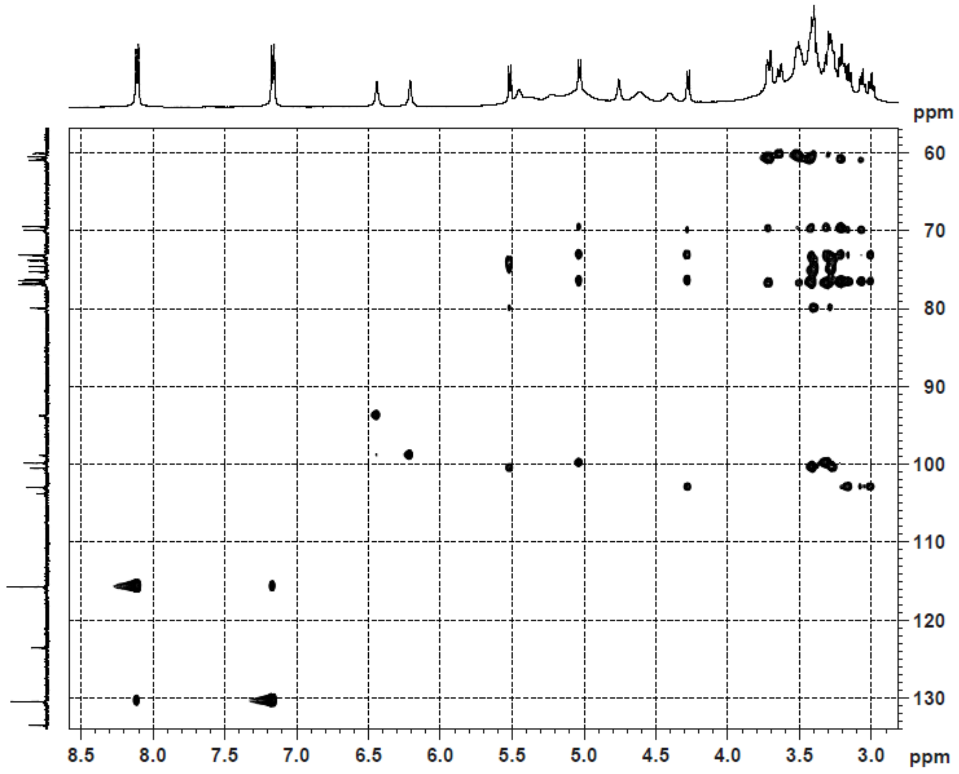

Fig. S14. HSQC-TOCSY (DMSO-d_6) spectrum of compound **2**

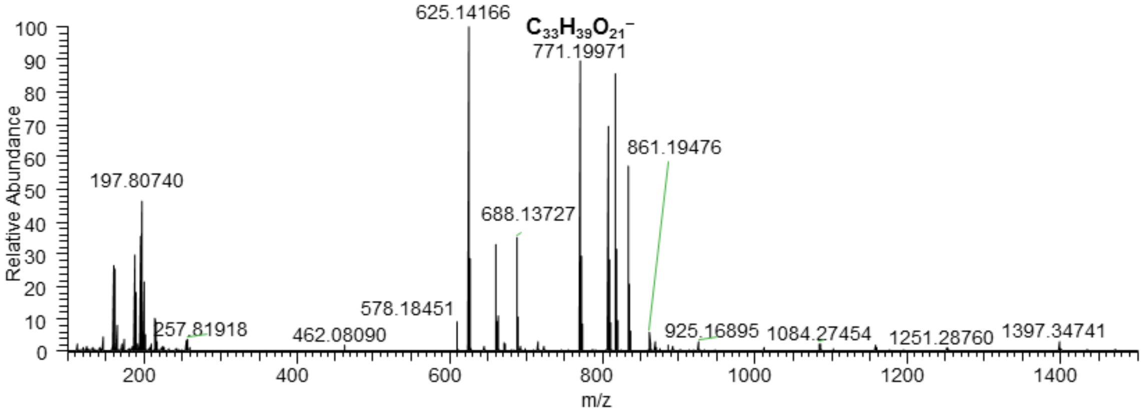

Fig. S15. ESI-Q-Orbitrap MS spectrum of compound **2**

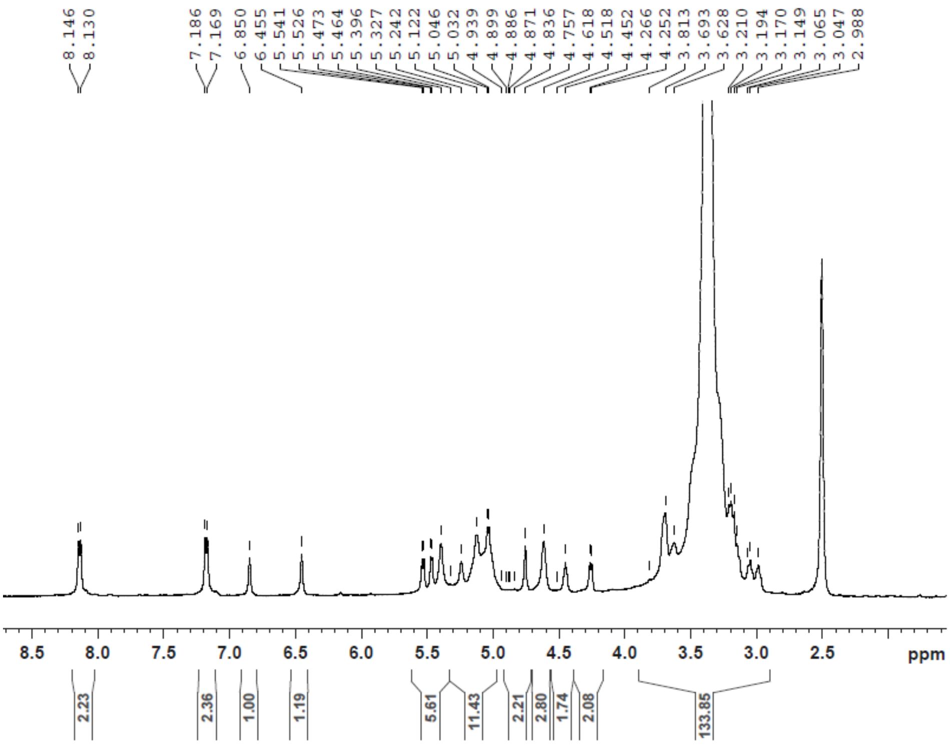

Fig. S16. ^{1}H NMR (500 MHz, DMSO-d_6) spectrum of compound **3**

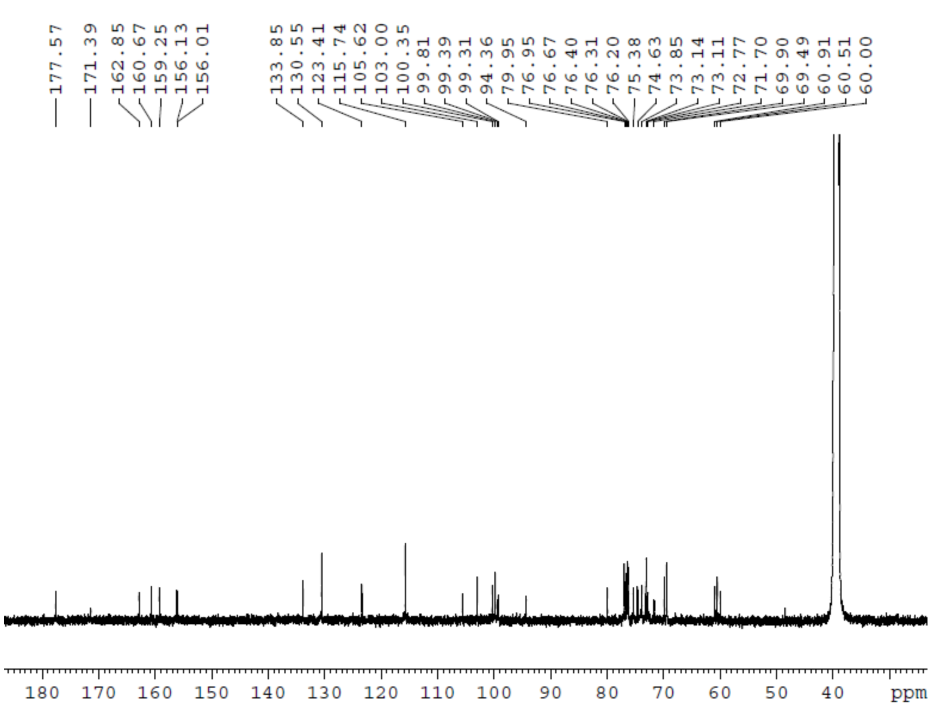

Fig. S17. ^{13}C NMR (125 MHz, DMSO-d_6) spectrum of compound **3**

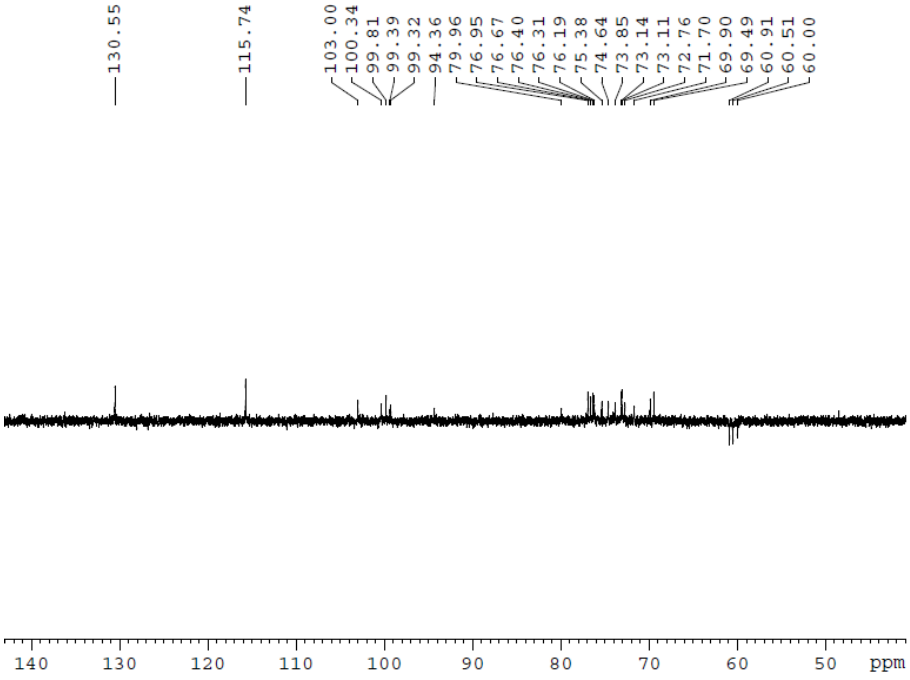

Fig. S18. DEPT 135 (DMSO-d_6) spectrum of compound **3**

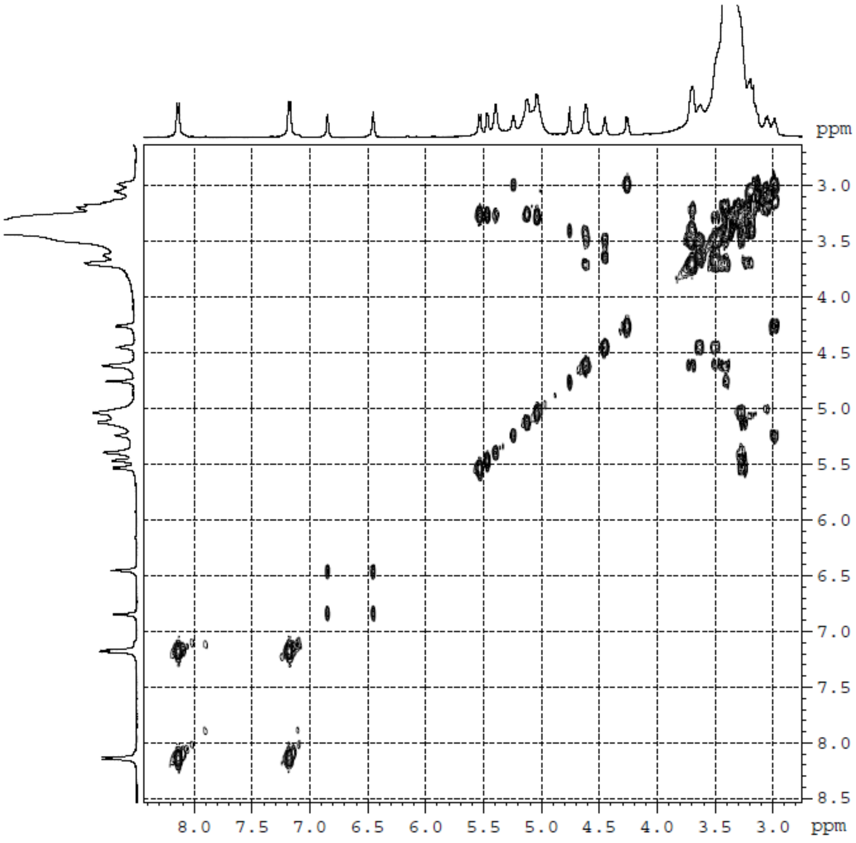

Fig. S19. ^1H ^1H COSY (DMSO-d_6) spectrum of compound **3**

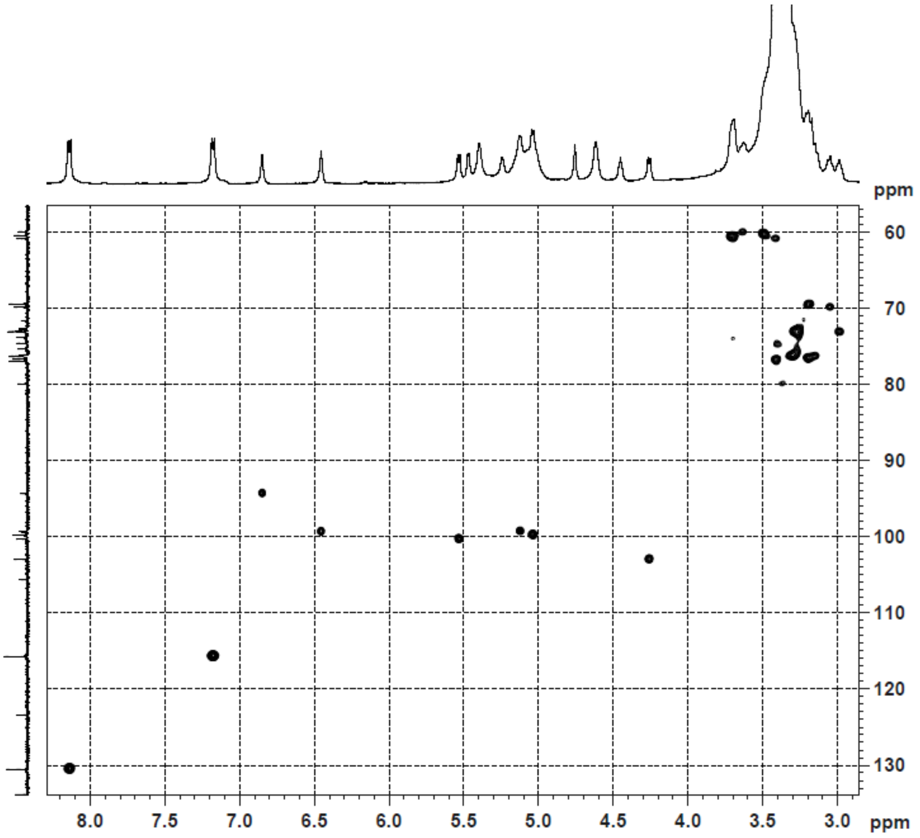

Fig. S20. HSQC (DMSO-d_6) spectrum of compound **3**

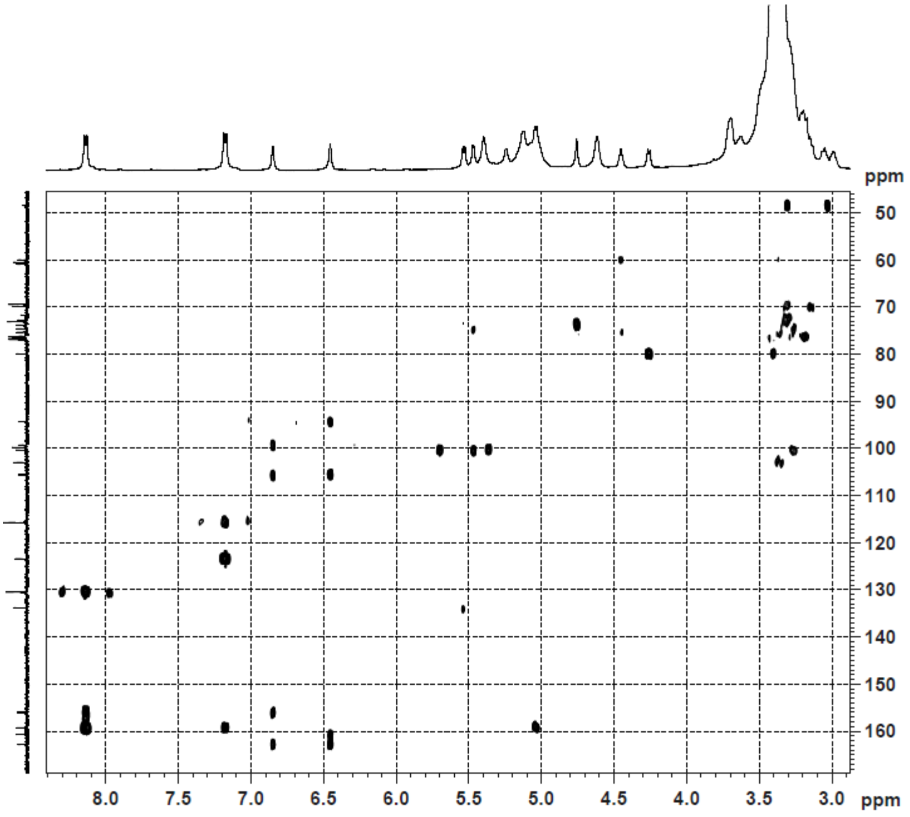

Fig. S21. HMBC (DMSO-d_6) spectrum of compound **3**

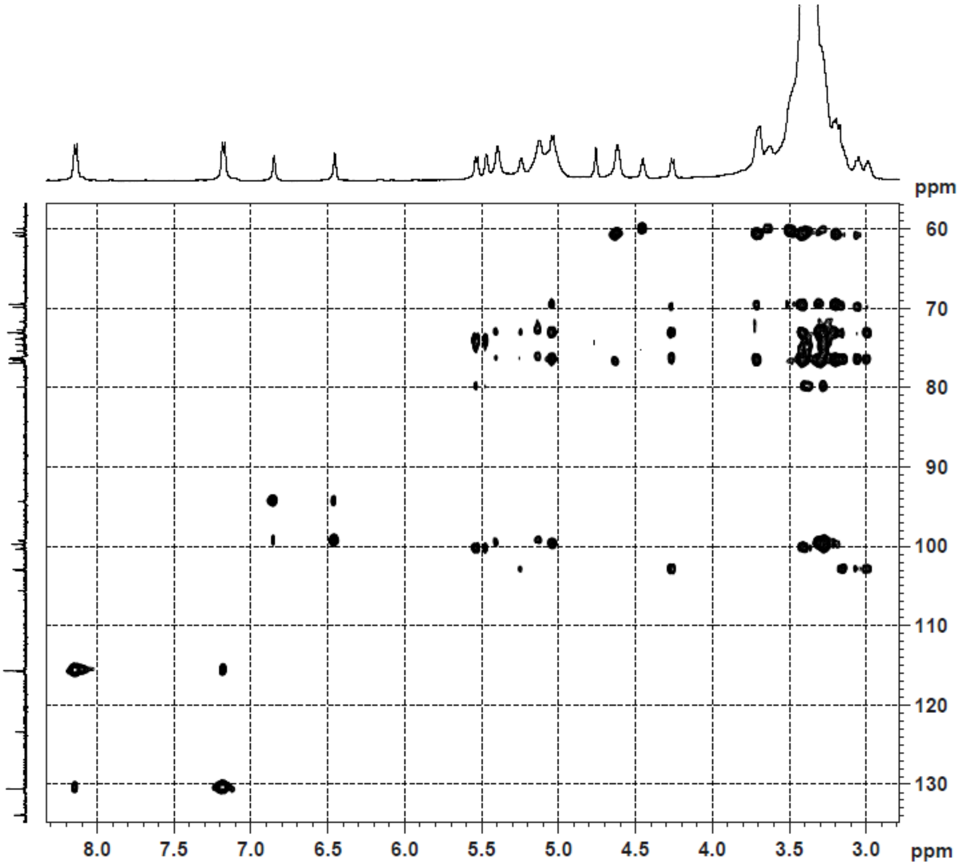

Fig. S22. HSQC-TOCSY (DMSO-d_6) spectrum of compound **3**

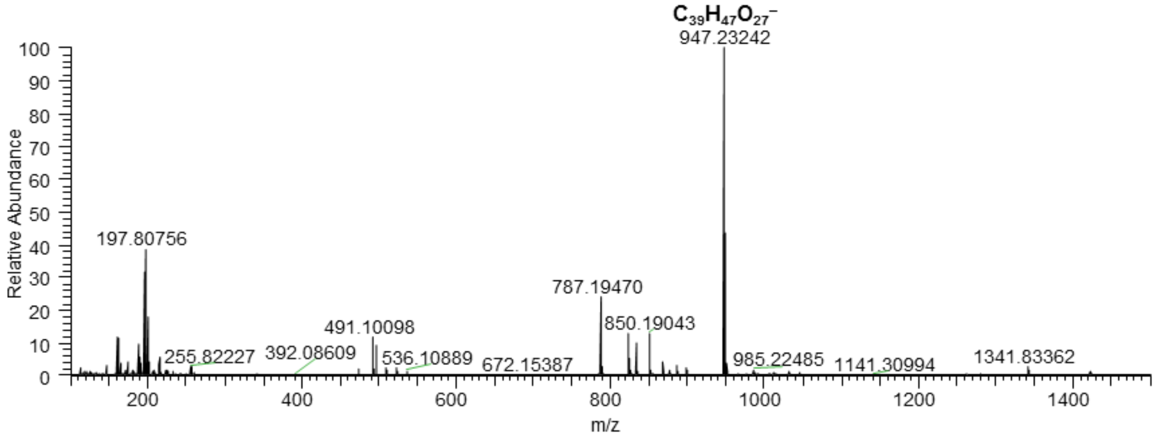

Fig. S23. ESI-Q-Orbitrap MS spectrum of compound **3**

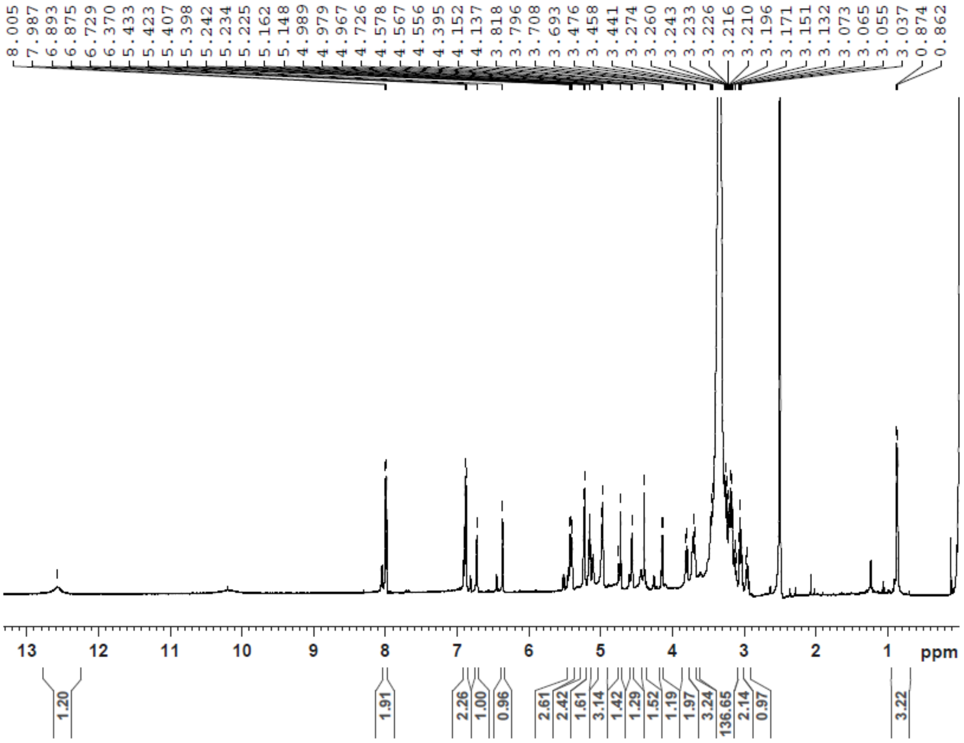

Fig. S24. ^{1}H NMR (500 MHz, DMSO-d_6) spectrum of compound **4**

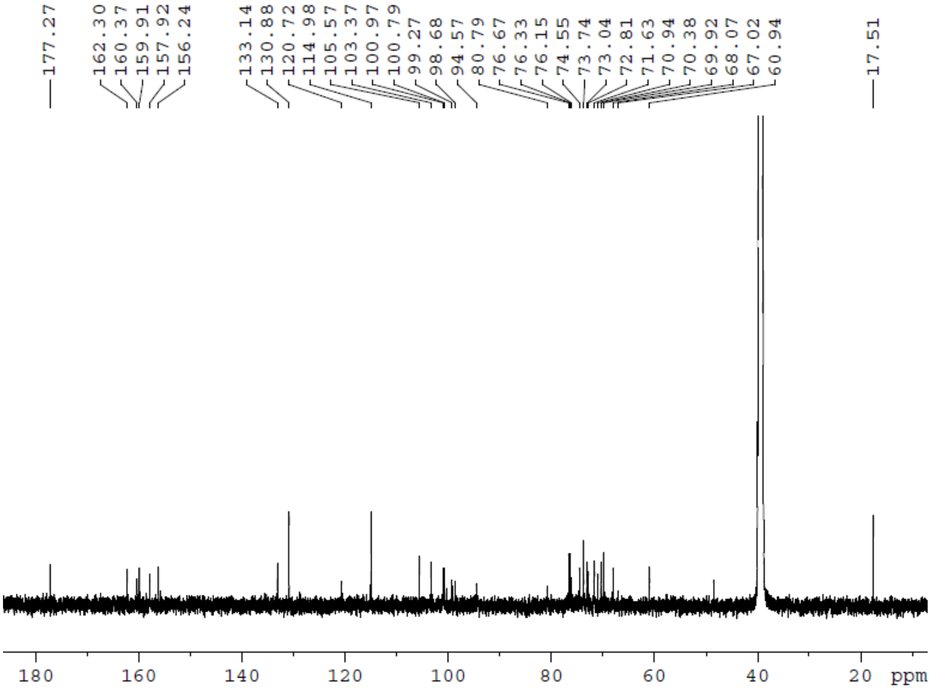

Fig. S25. ^{13}C NMR (125 MHz, DMSO-d_6) spectrum of compound **4**

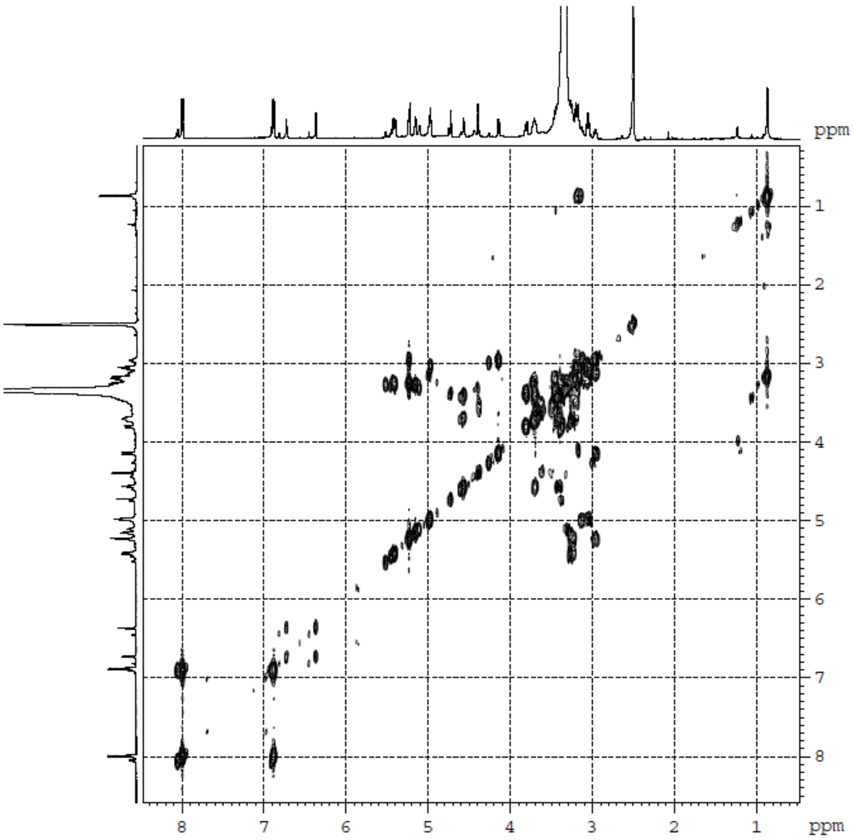

Fig. S26. ^{1}H ^{1}H COSY (DMSO-*d*$_6$) spectrum of compound **4**

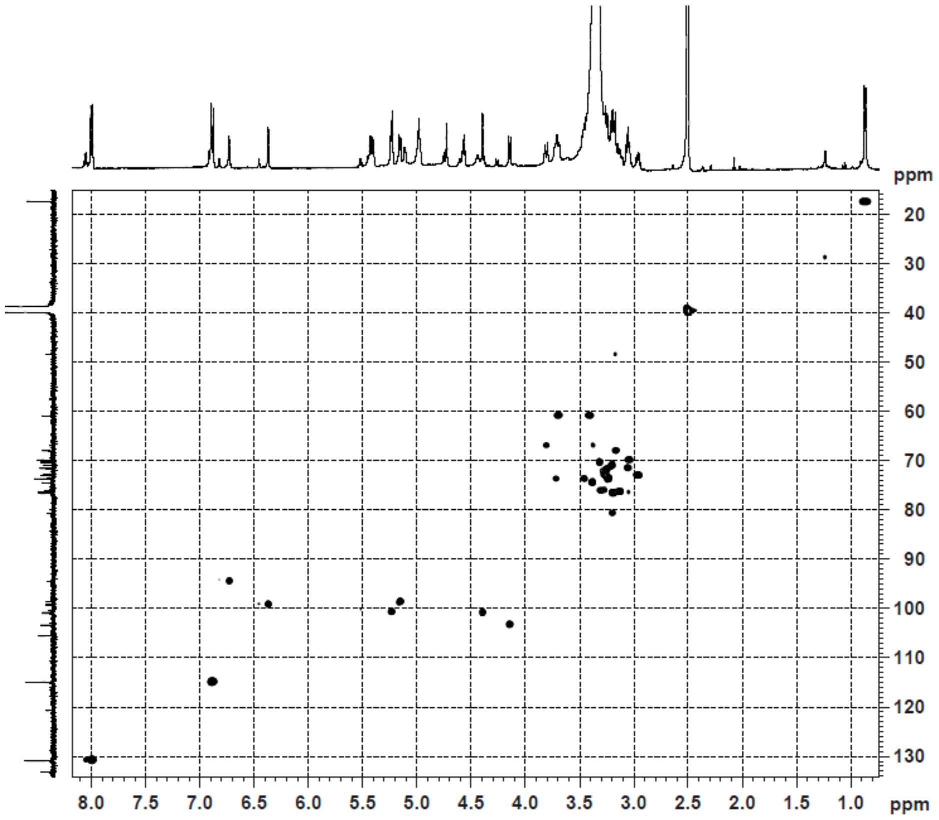

Fig. S27. HSQC (DMSO-*d*$_6$) spectrum of compound **4**

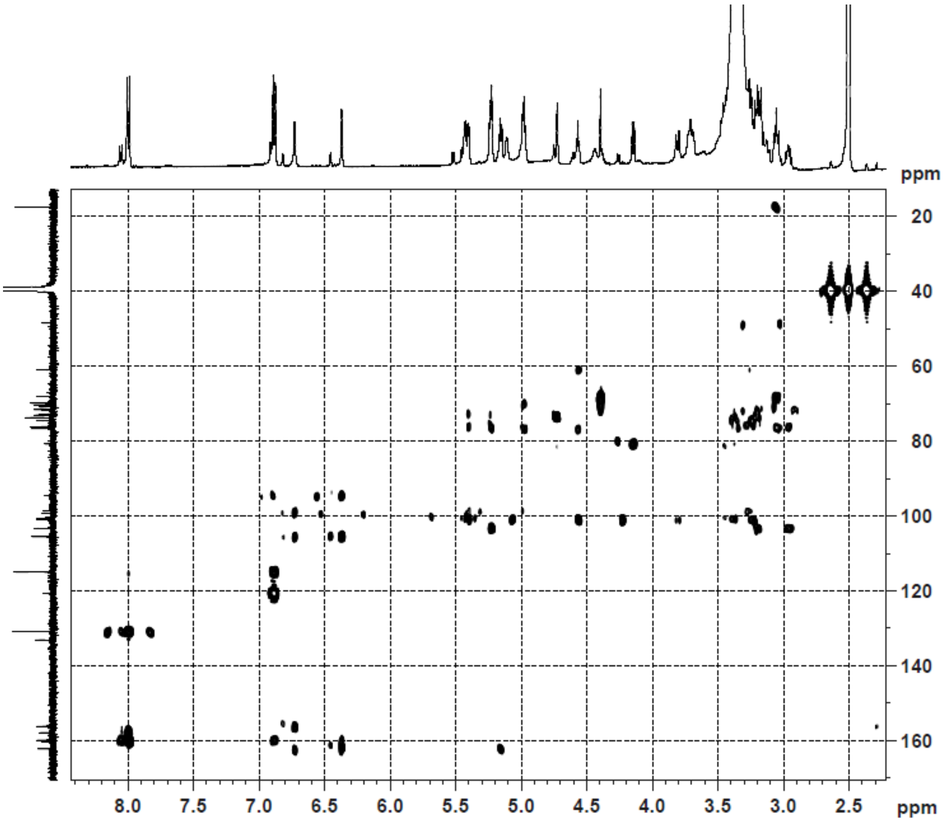

Fig. S28. HMBC (DMSO-d_6) spectrum of compound **4**

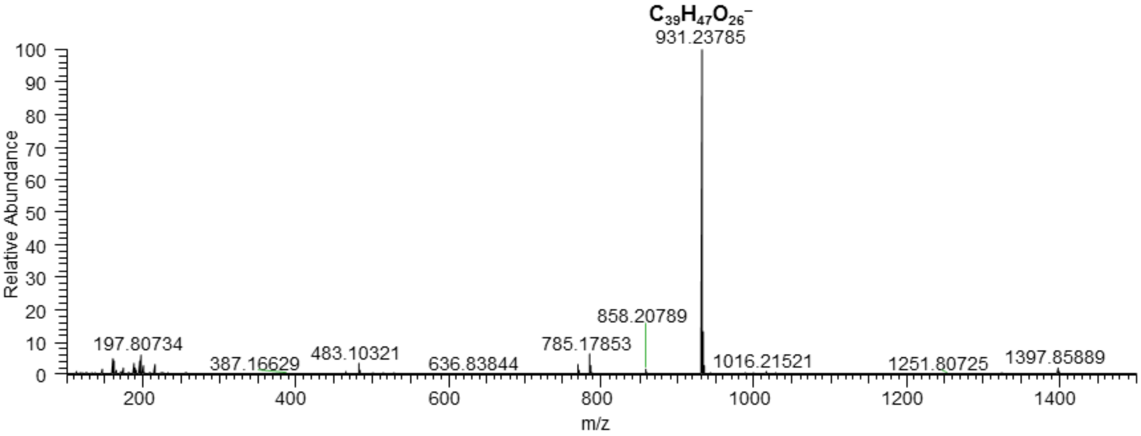

Fig. S29. ESI-Q-Orbitrap MS spectrum of compound **4**

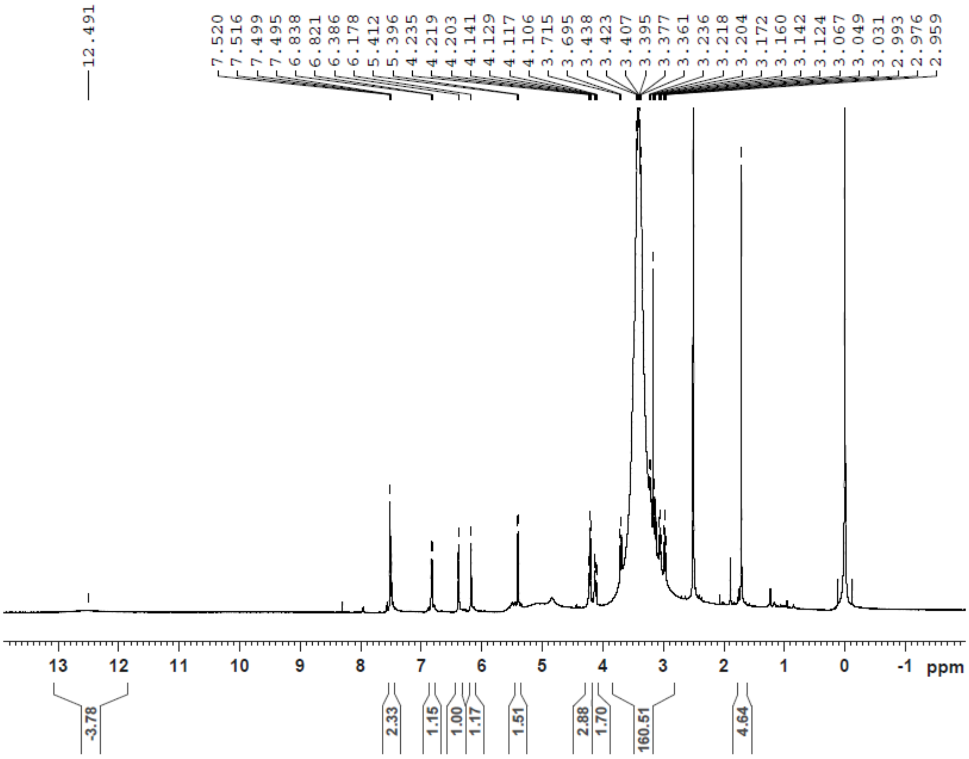

Fig. S30. ^{1}H NMR (500 MHz, DMSO-*d*6) spectrum of compound **5**

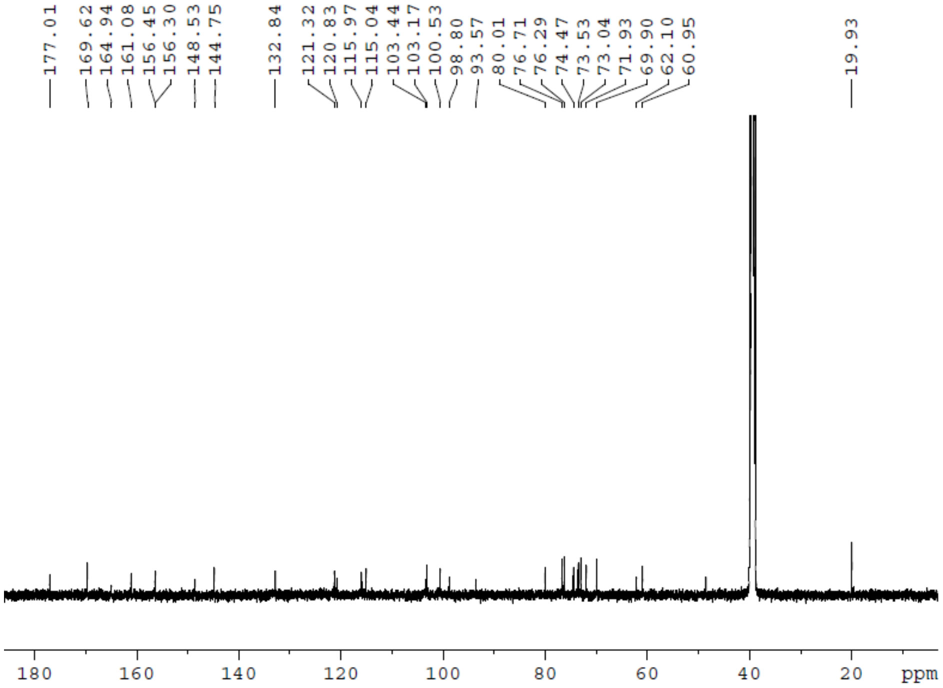

Fig. S31. ^{13}C NMR (125 MHz, DMSO-*d*6) spectrum of compound **5**

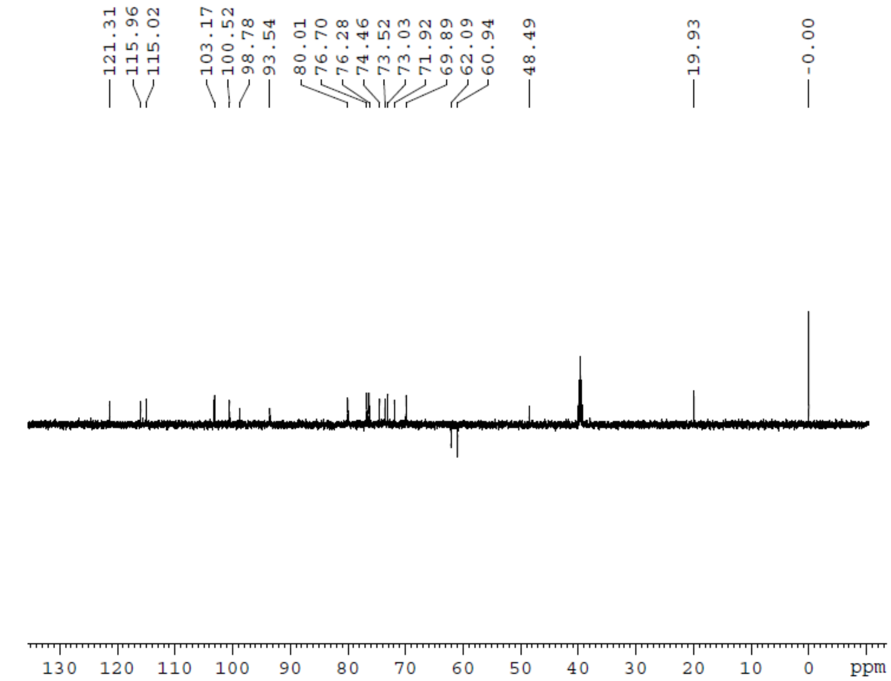

Fig. S32. DEPT 135 (DMSO-d_6) spectrum of compound **5**

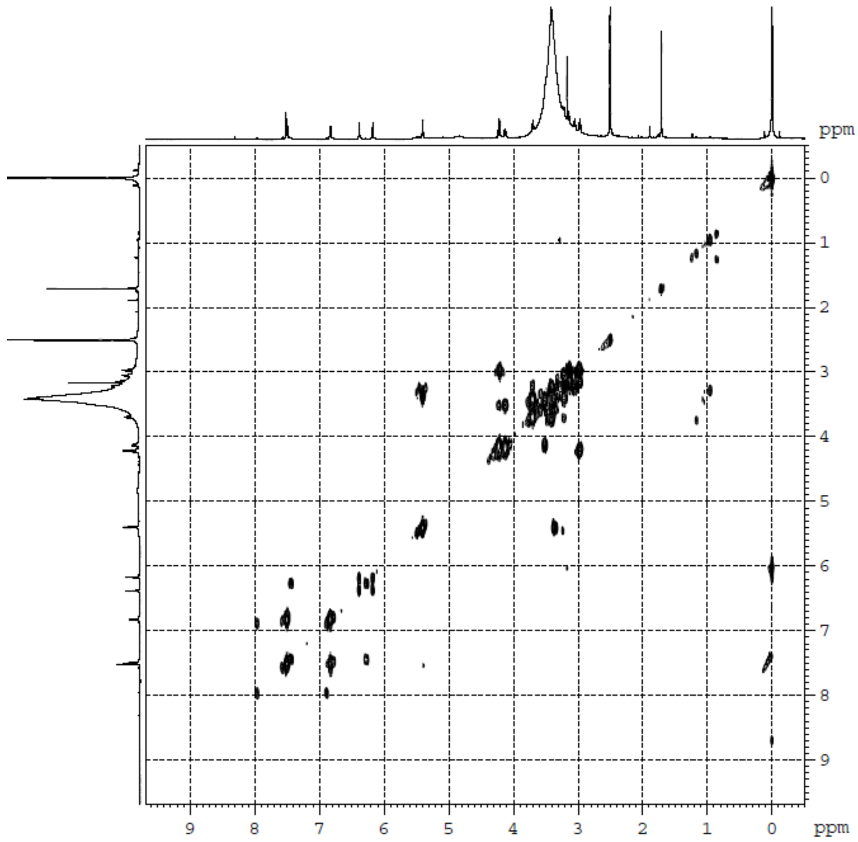

Fig. S33. ^{1}H ^{1}H COSY (DMSO-d_6) spectrum of compound **5**

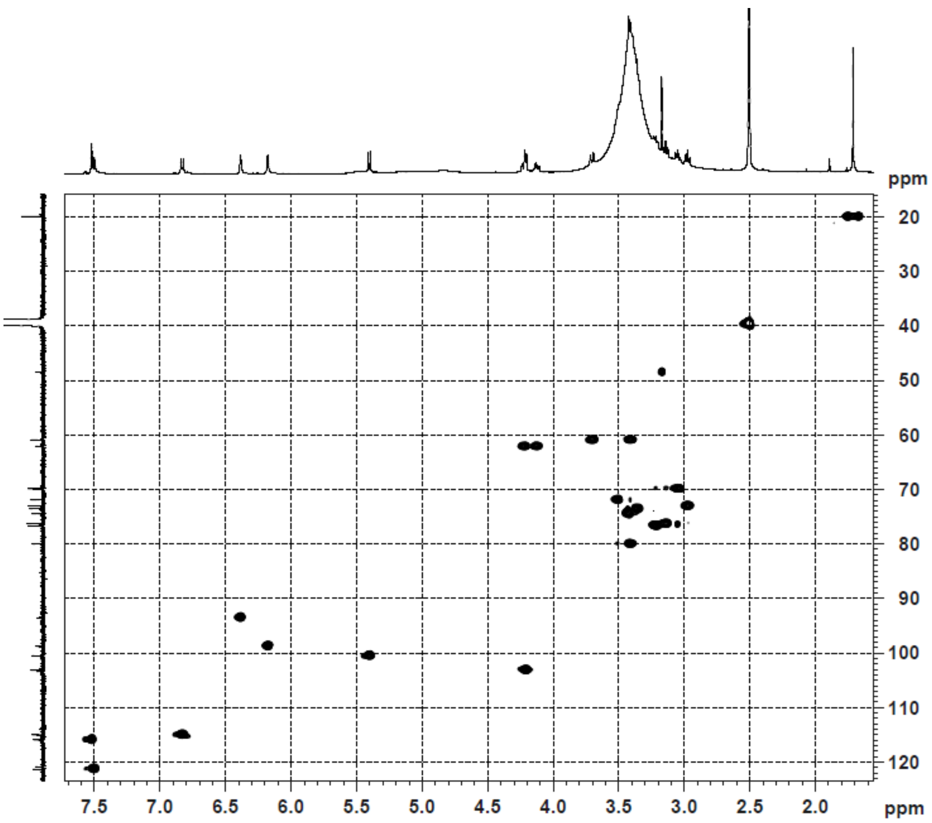

Fig. S34. HSQC (DMSO-d_6) spectrum of compound **5**

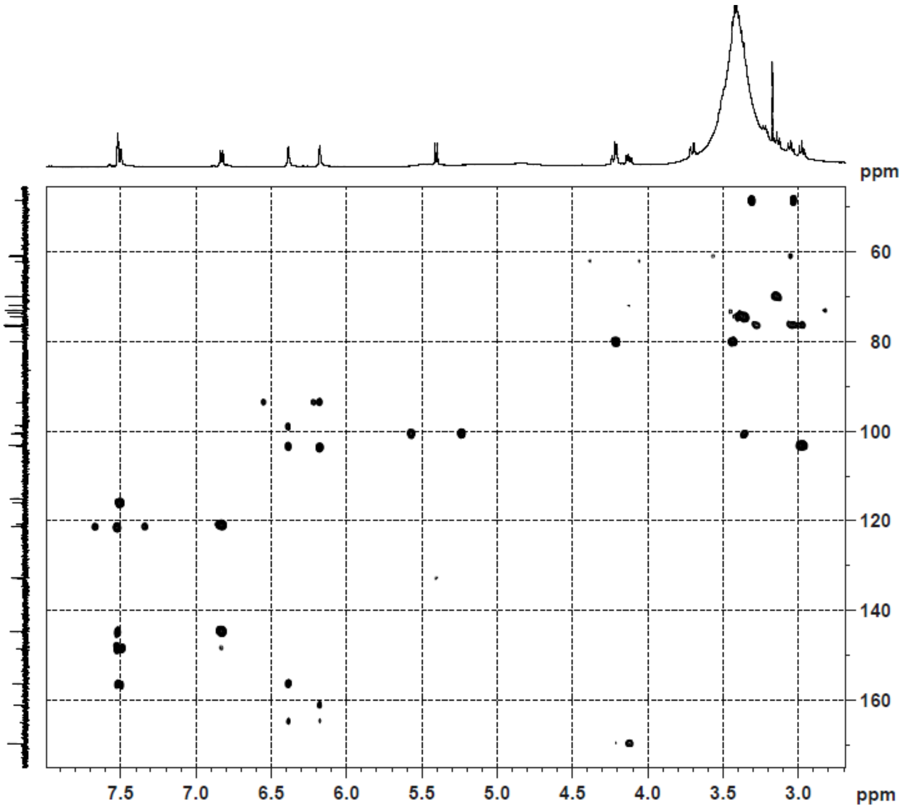

Fig. S35. HMBC (DMSO-d_6) spectrum of compound **5**

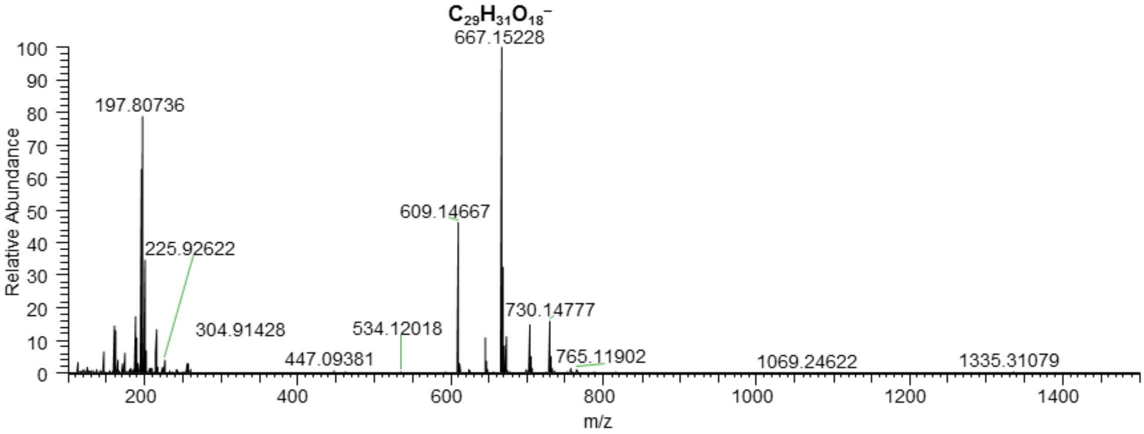

Fig. S36. ESI-Q-Orbitrap MS spectrum of compound **5**

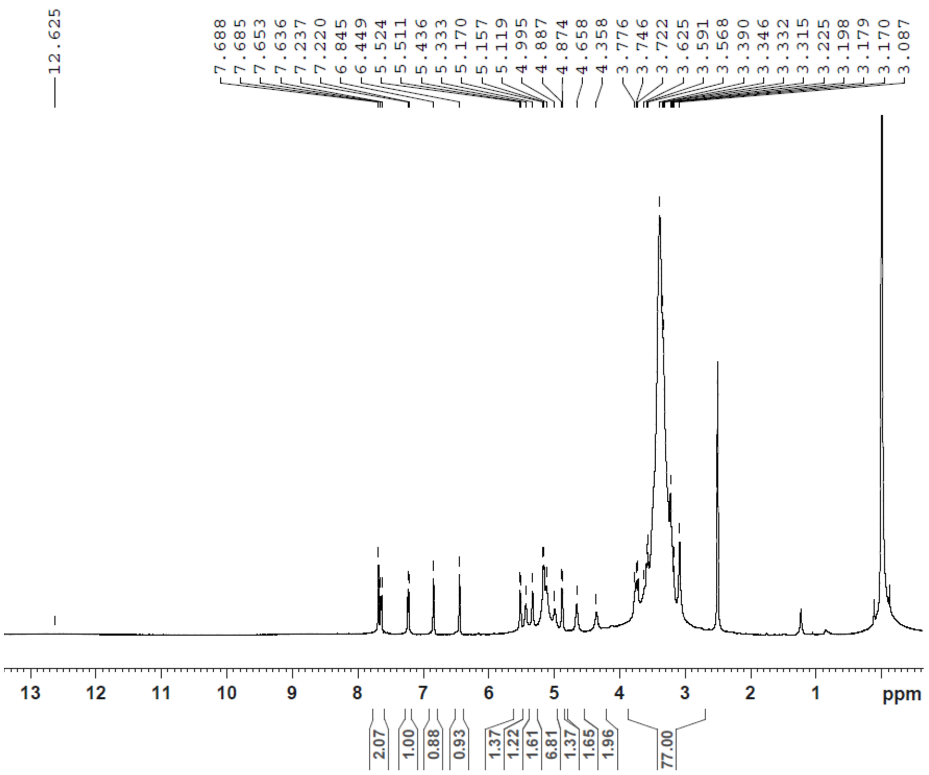

Fig. S37. ^{1}H NMR (500 MHz, DMSO-d_6) spectrum of compound **6**

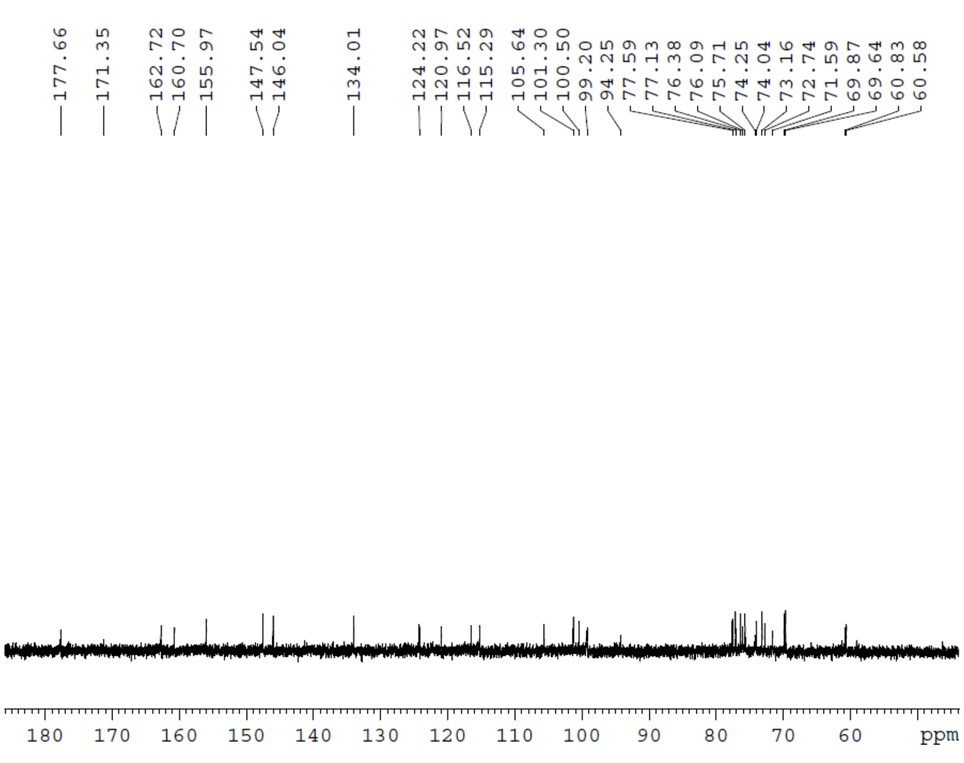

Fig. S38. ^{13}C NMR (125 MHz, DMSO-d_6) spectrum of compound **6**

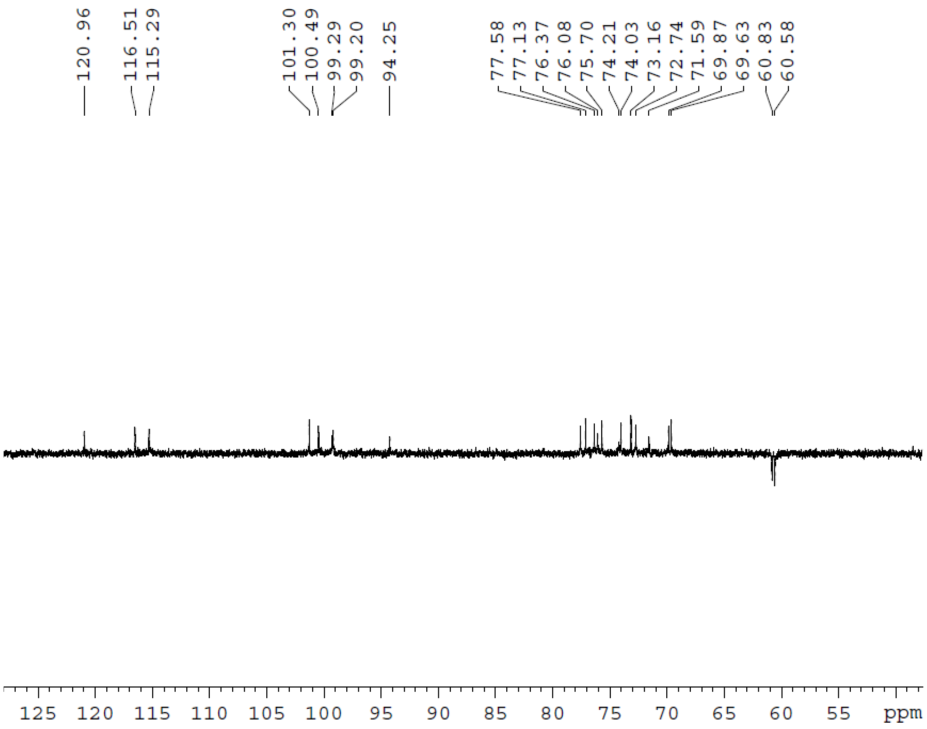

Fig. S39. DEPT 135 (DMSO-d_6) spectrum of compound **6**

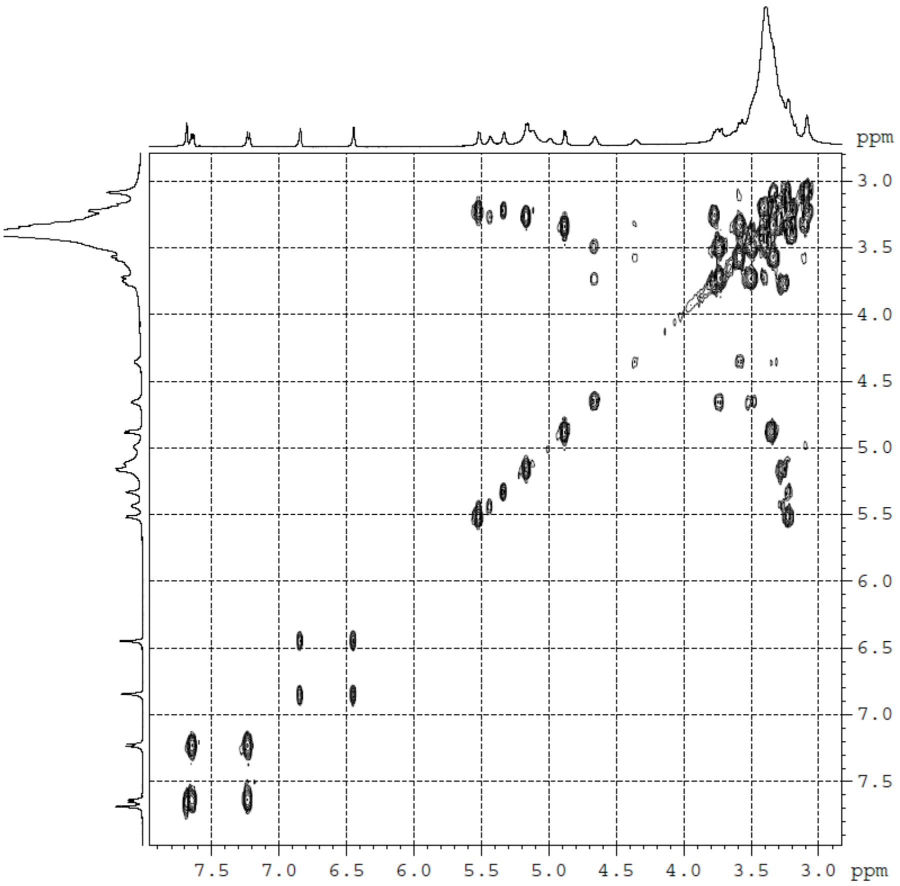

Fig. S40. ^{1}H ^{1}H COSY (DMSO-d_6) spectrum of compound **6**

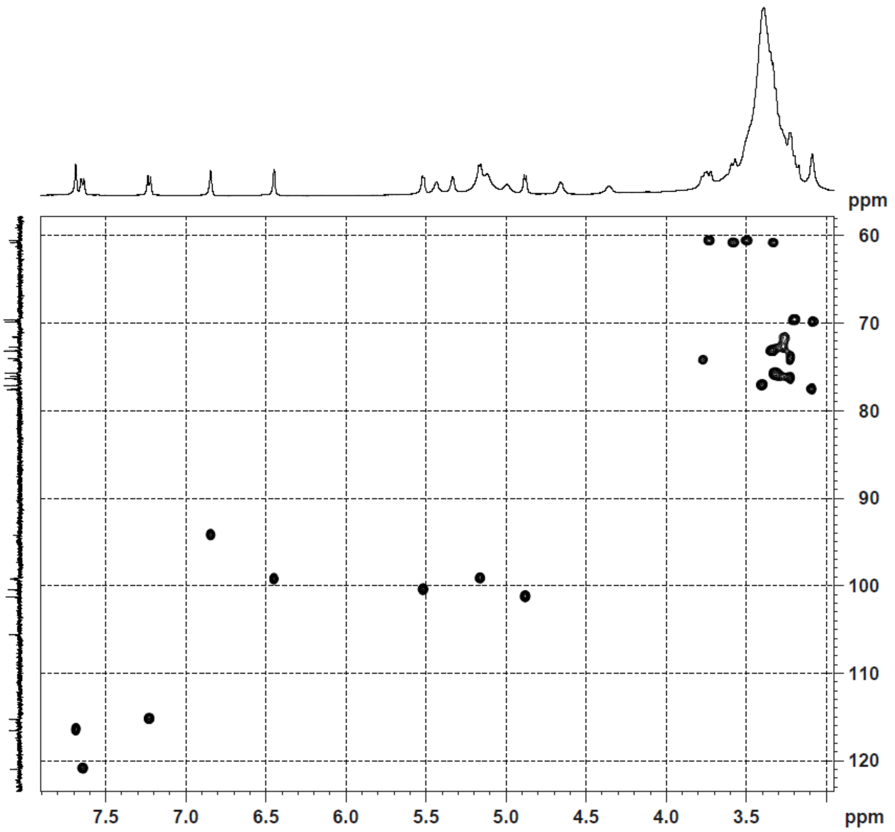

Fig. S41. HSQC (DMSO-d_6) spectrum of compound **6**

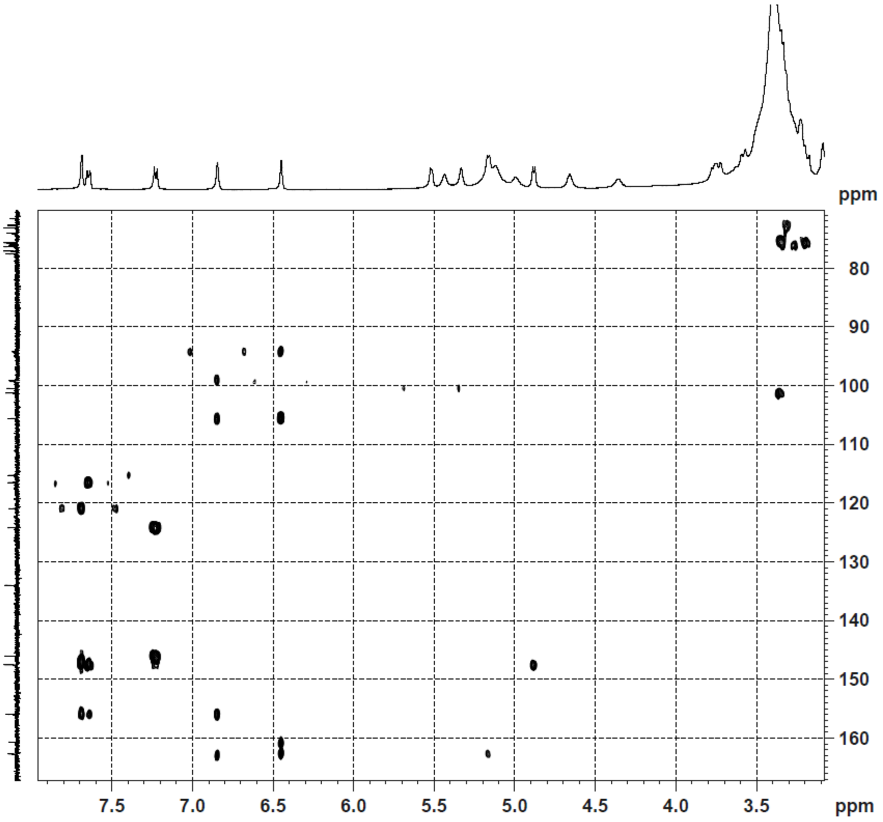

Fig. S42. HMBC (DMSO-d_6) spectrum of compound **6**

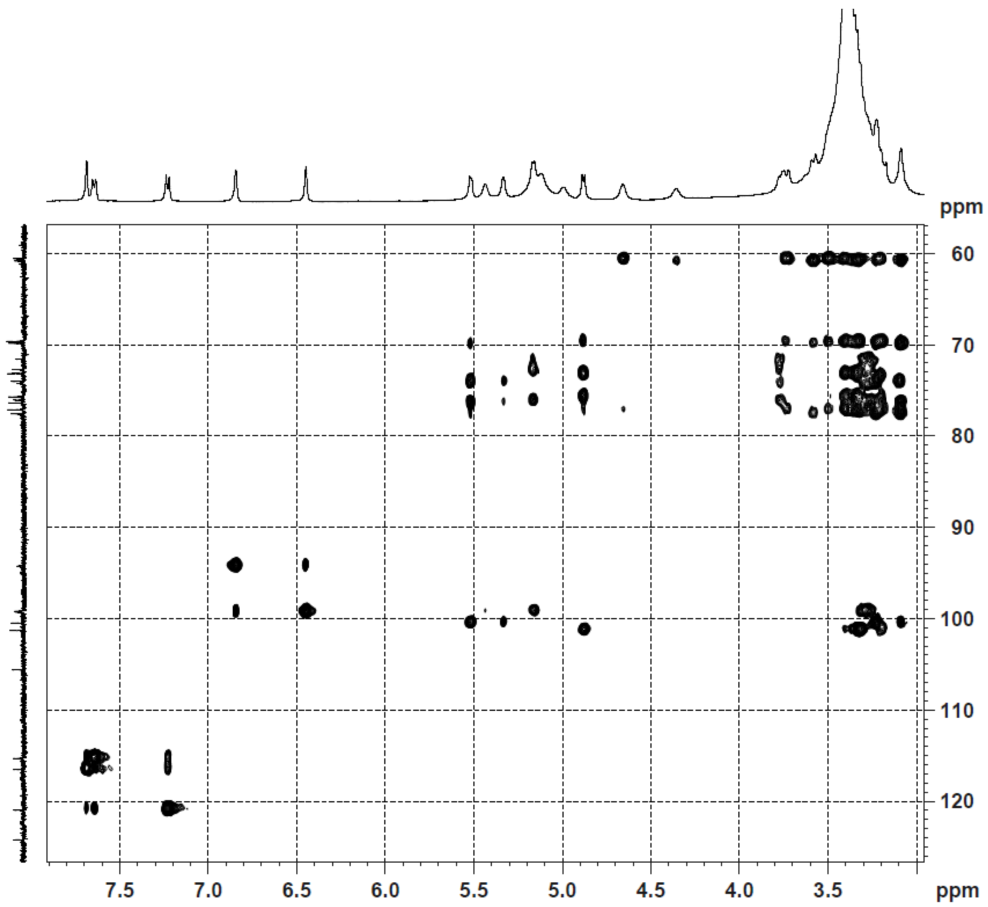

Fig. S43. HSQC-TOCSY (DMSO-d_6) spectrum of compound **6**

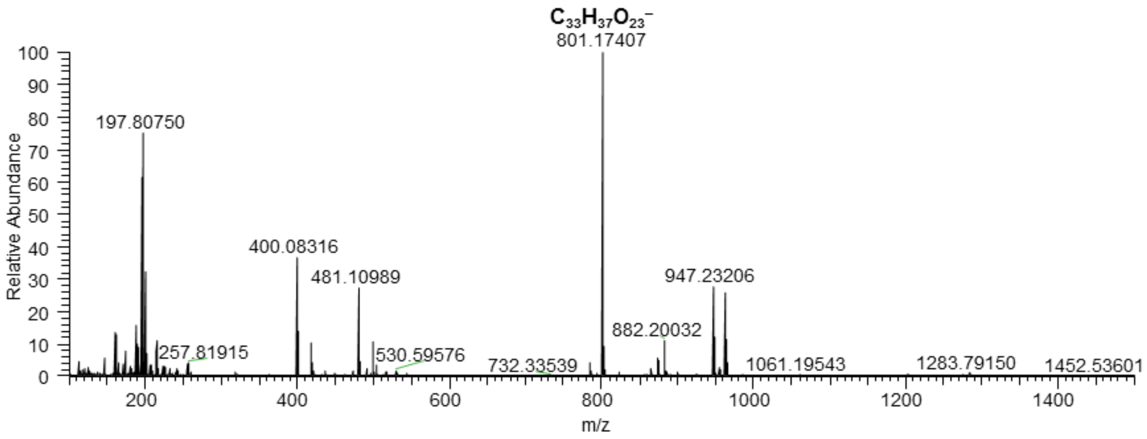

Fig. S44. ESI-Q-Orbitrap MS spectrum of compound **6**

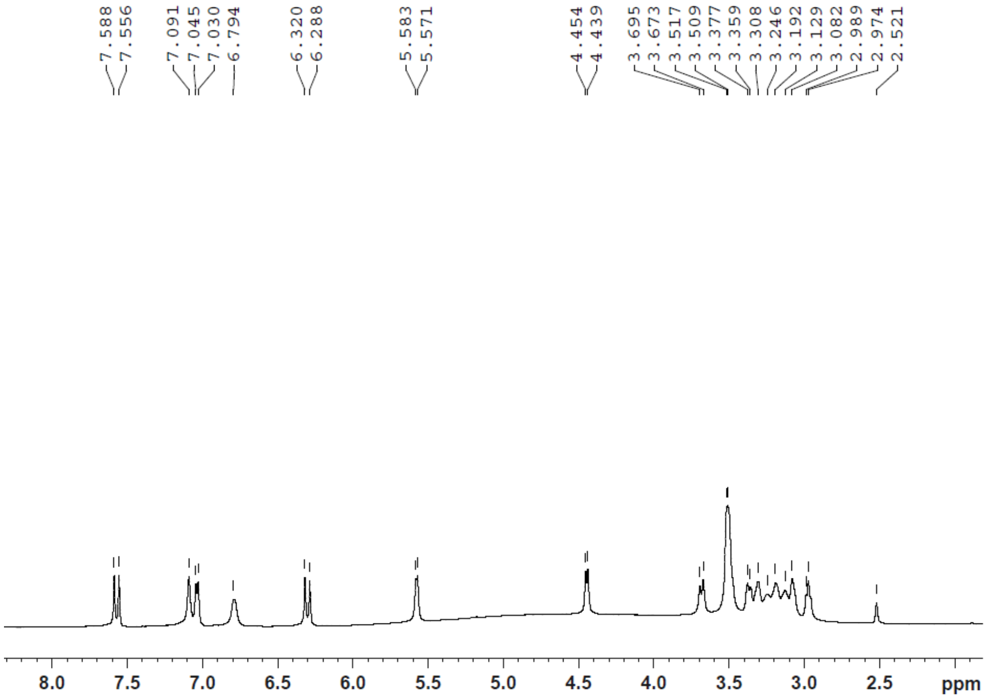

Fig. S45. ^{1}H NMR (500 MHz, DMSO-d_6) spectrum of compound **7**

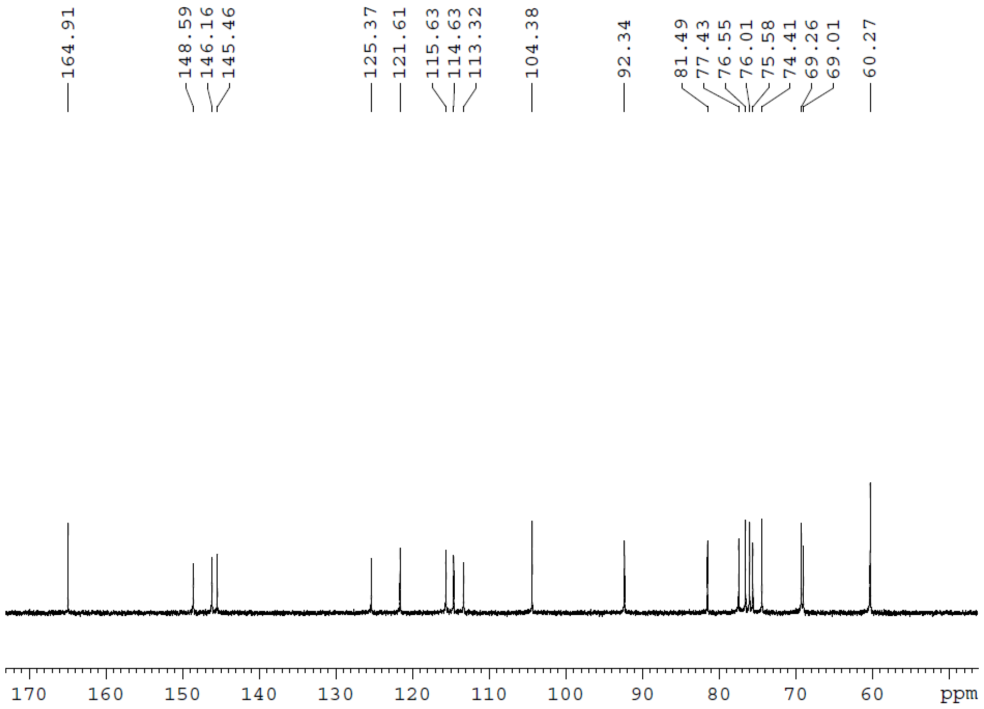

Fig. S46. ^{13}C NMR (125 MHz, DMSO-d_6) spectrum of compound **7**

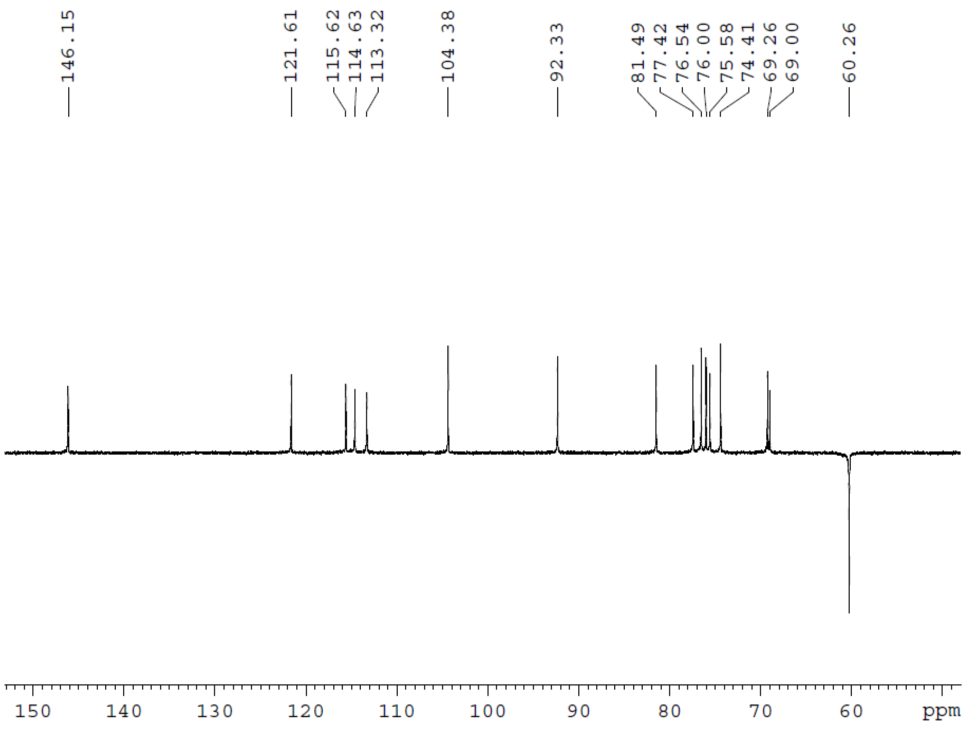

Fig. S47. DEPT 135 (DMSO-d_6) spectrum of compound **7**

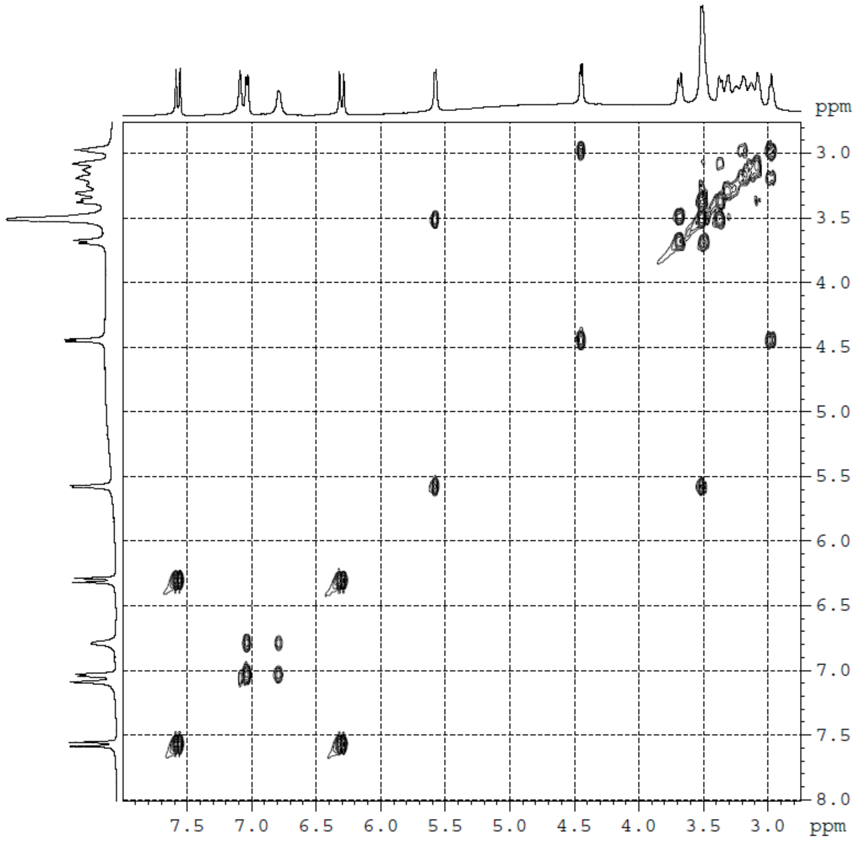

Fig. S48. ^1H ^1H COSY (DMSO-d_6) spectrum of compound **7**

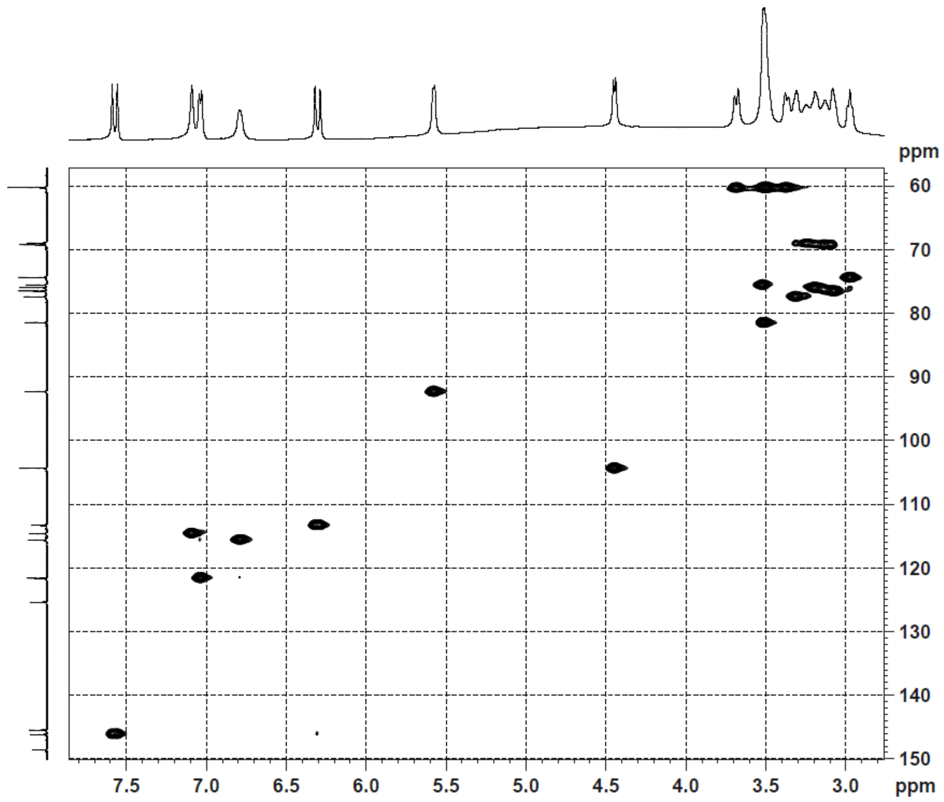

Fig. S49. HSQC (DMSO-d_6) spectrum of compound **7**

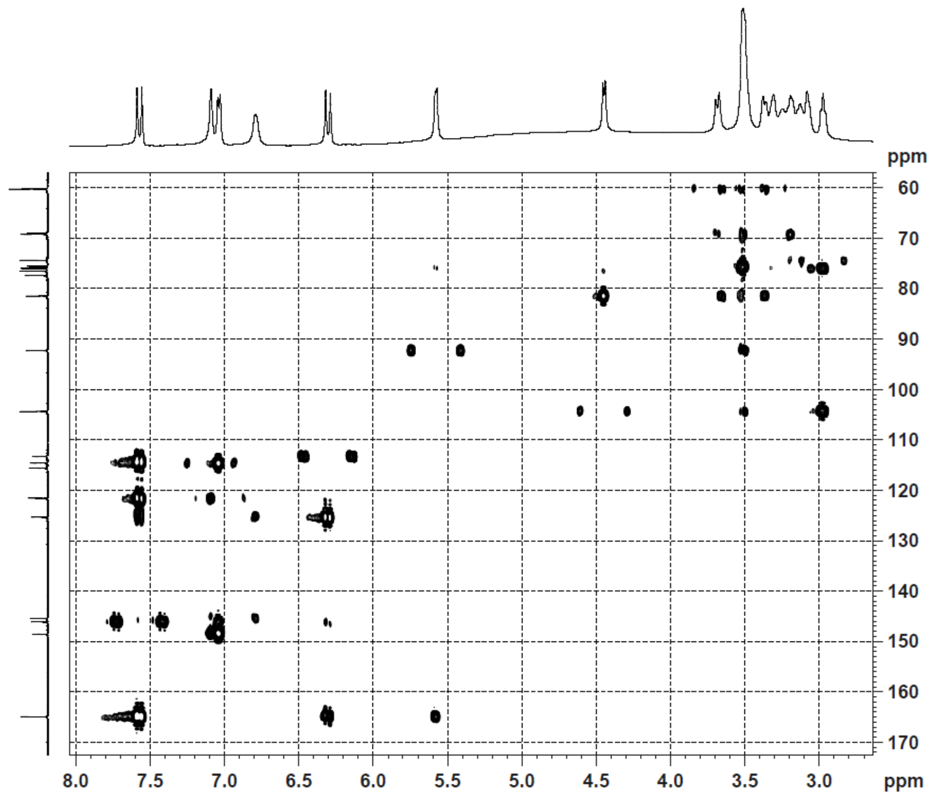

Fig. S50. HMBC (DMSO-d_6) spectrum of compound **7**

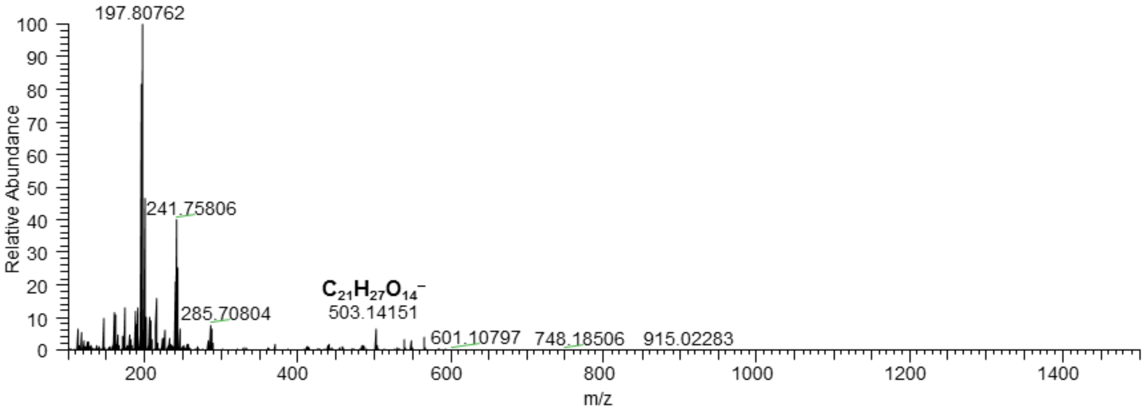

Fig. S51. ESI-Q-Orbitrap MS spectrum of compound **7**

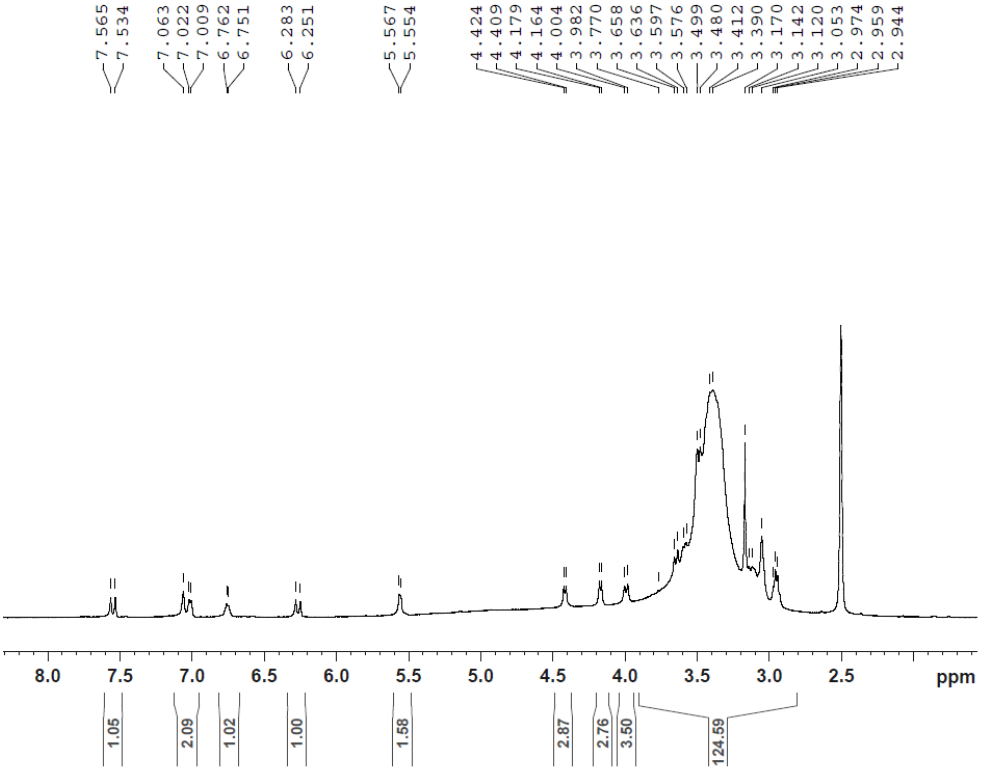

Fig. S52. ^{1}H NMR (500 MHz, DMSO-d_6) spectrum of compound **8**

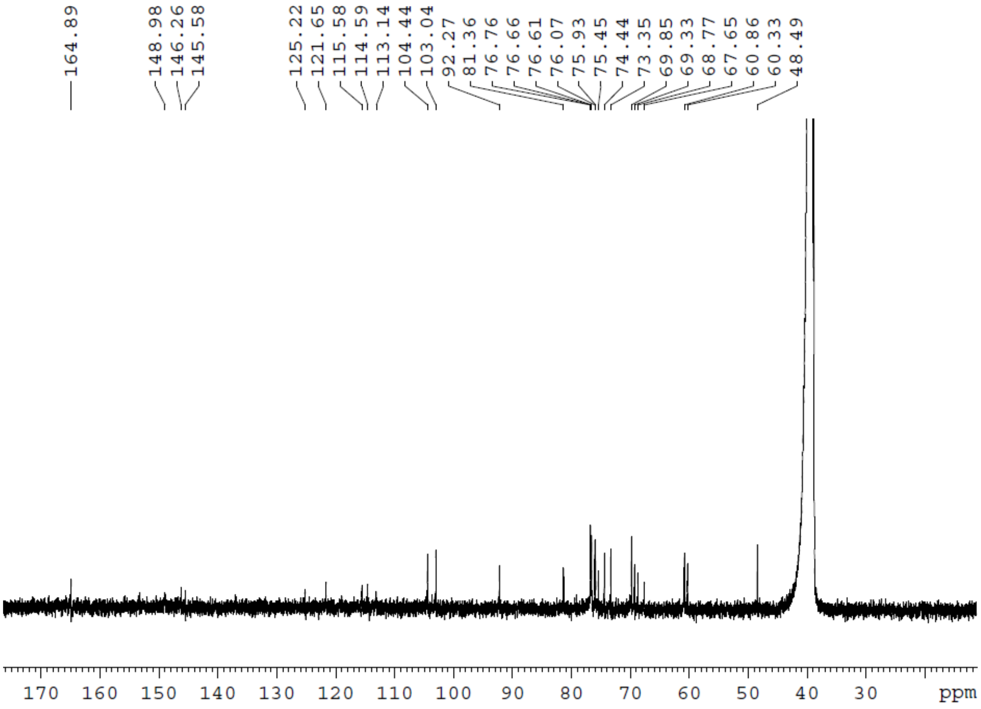

Fig. S53. ^{13}C NMR (125 MHz, DMSO-d_6) spectrum of compound **8**

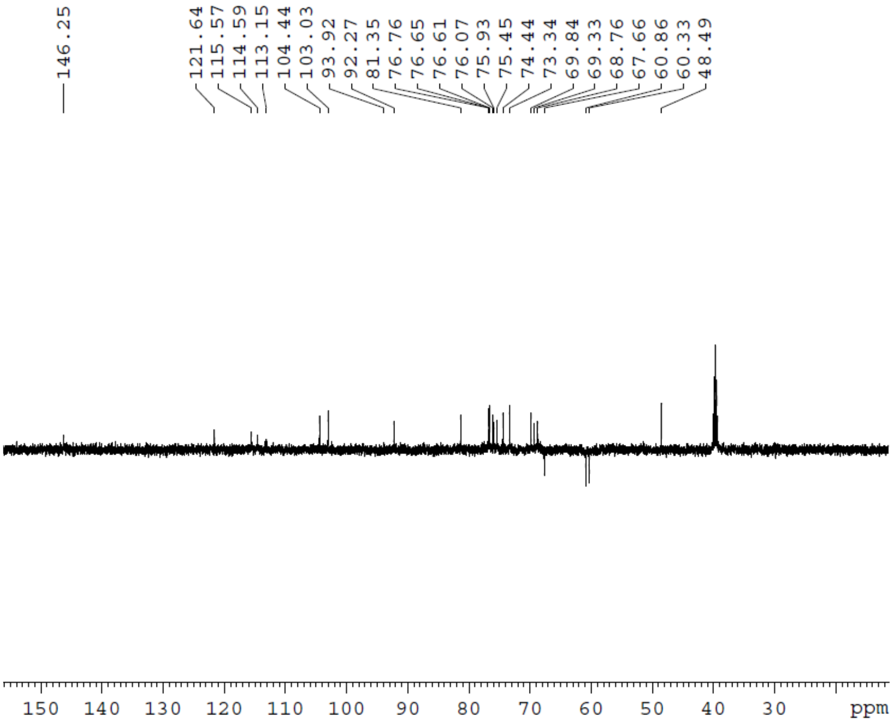

Fig. S54. DEPT 135 (DMSO-d_6) spectrum of compound **8**

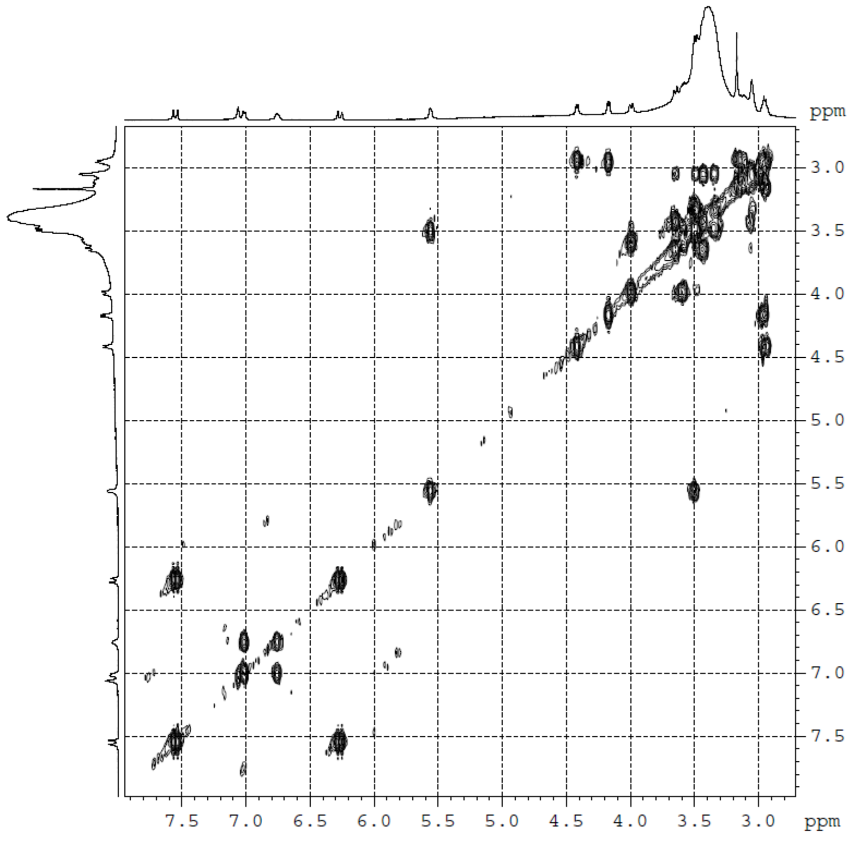

Fig. S55. ^1H ^1H COSY (DMSO-d_6) spectrum of compound **8**

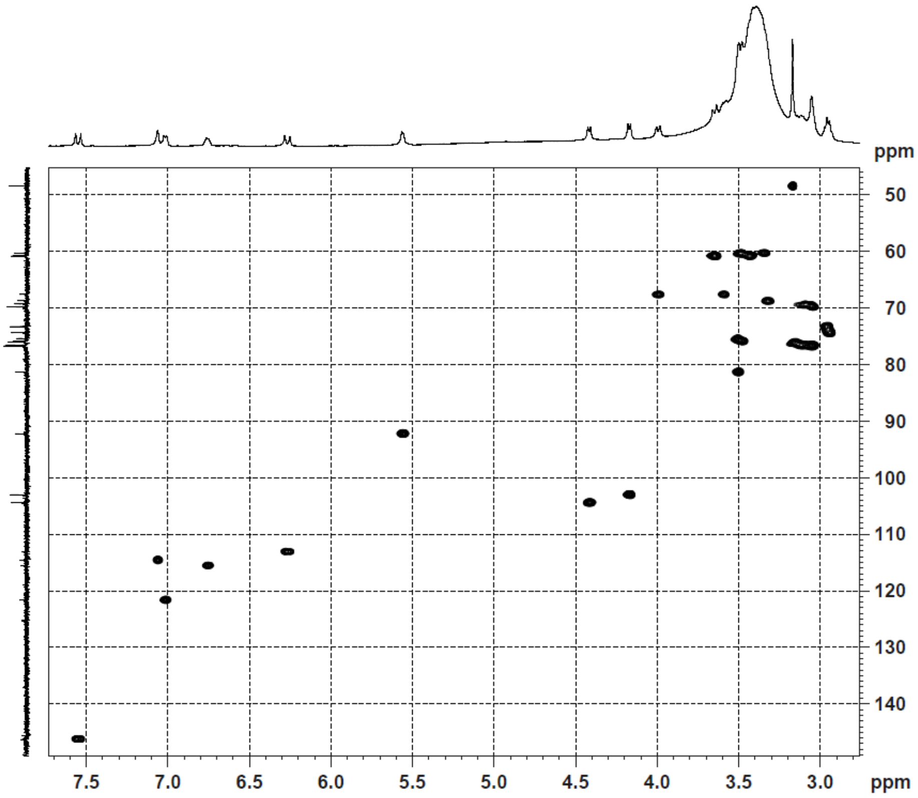

Fig. S56. HSQC (DMSO-d_6) spectrum of compound **8**

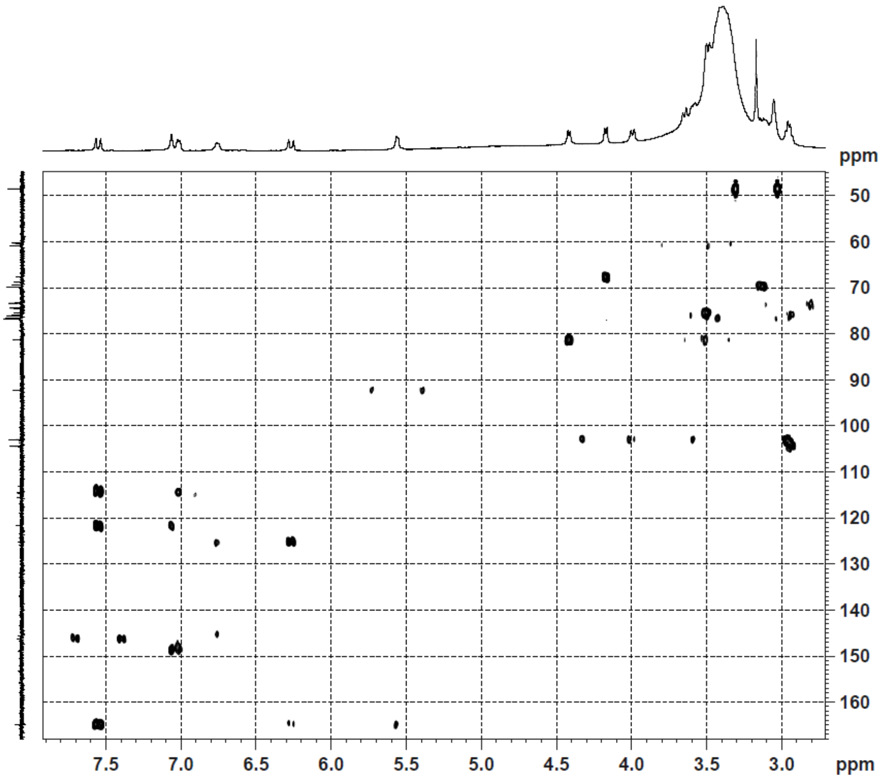

Fig. S57. HMBC (DMSO-d_6) spectrum of compound **8**

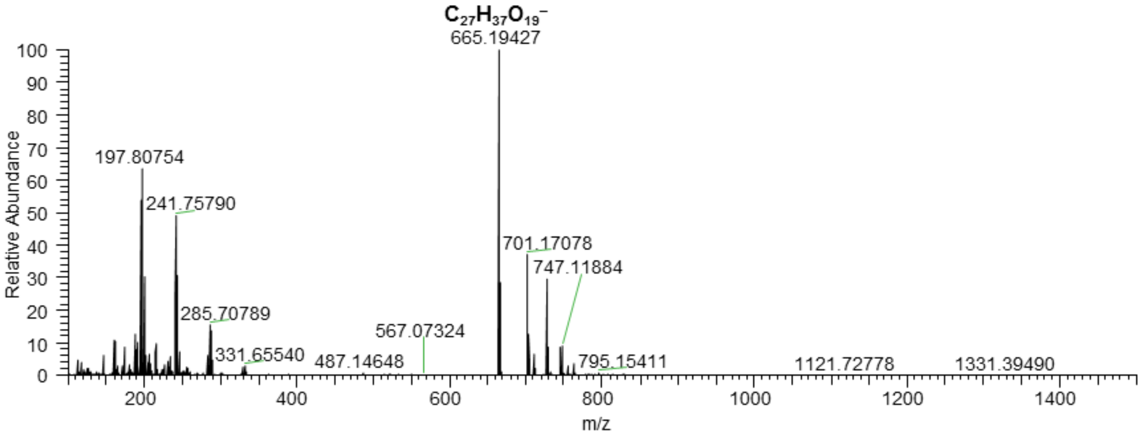

Fig. S58. ESI-Q-Orbitrap MS spectrum of compound **8**

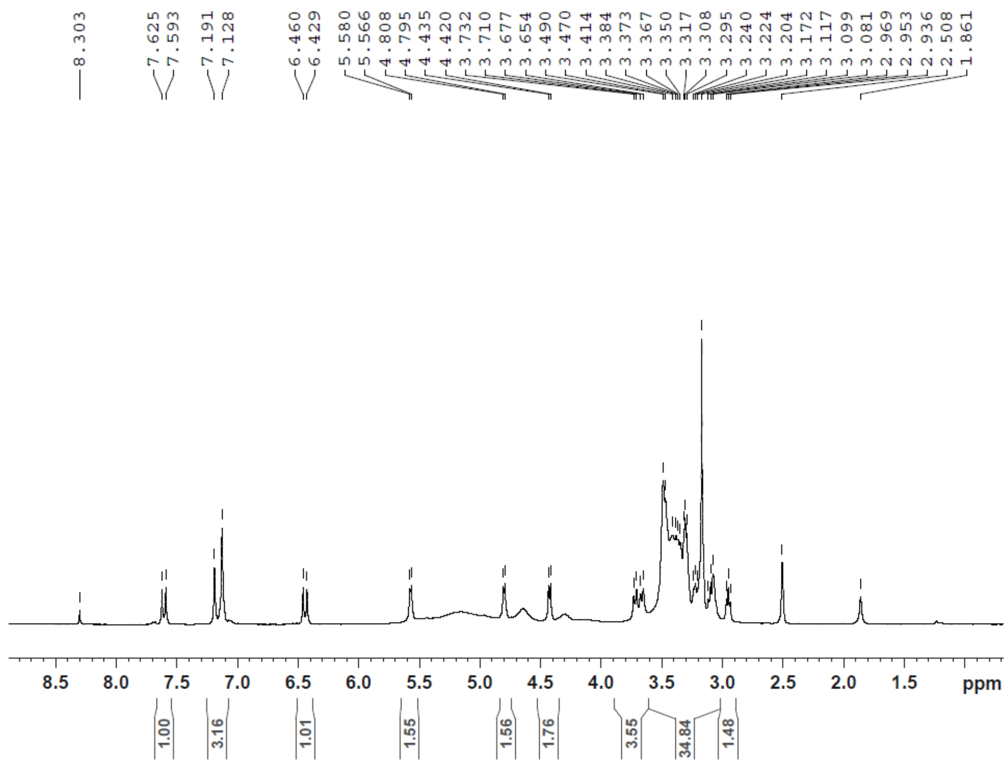

Fig. S59. ^{1}H NMR (500 MHz, DMSO-d_6) spectrum of compound **9**

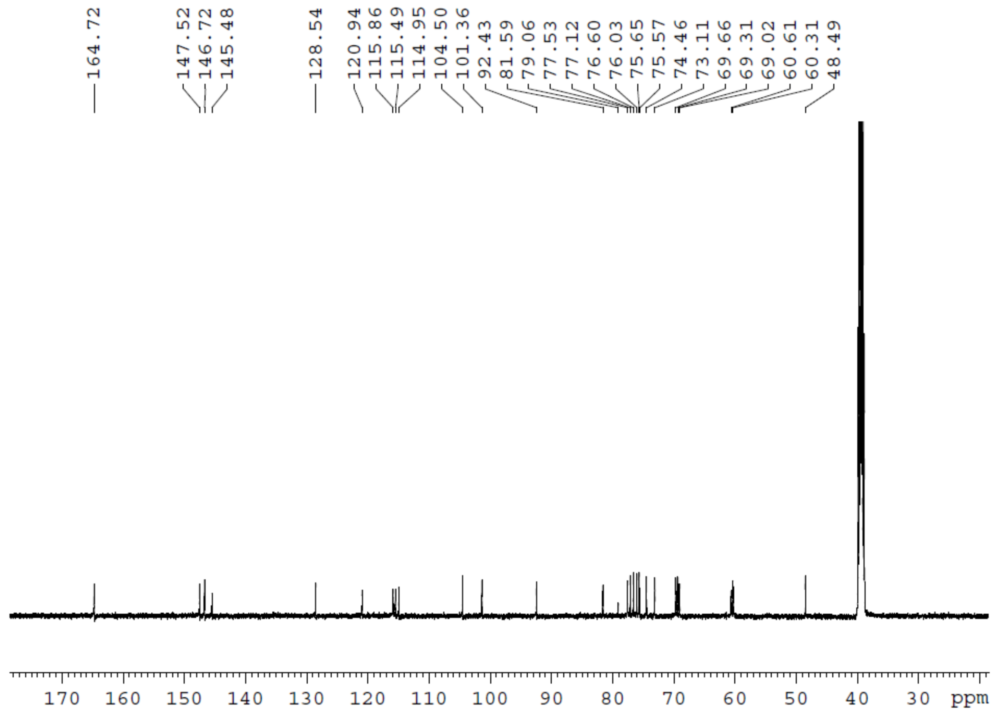

Fig. S60. ^{13}C NMR (125 MHz, DMSO-d_6) spectrum of compound **9**

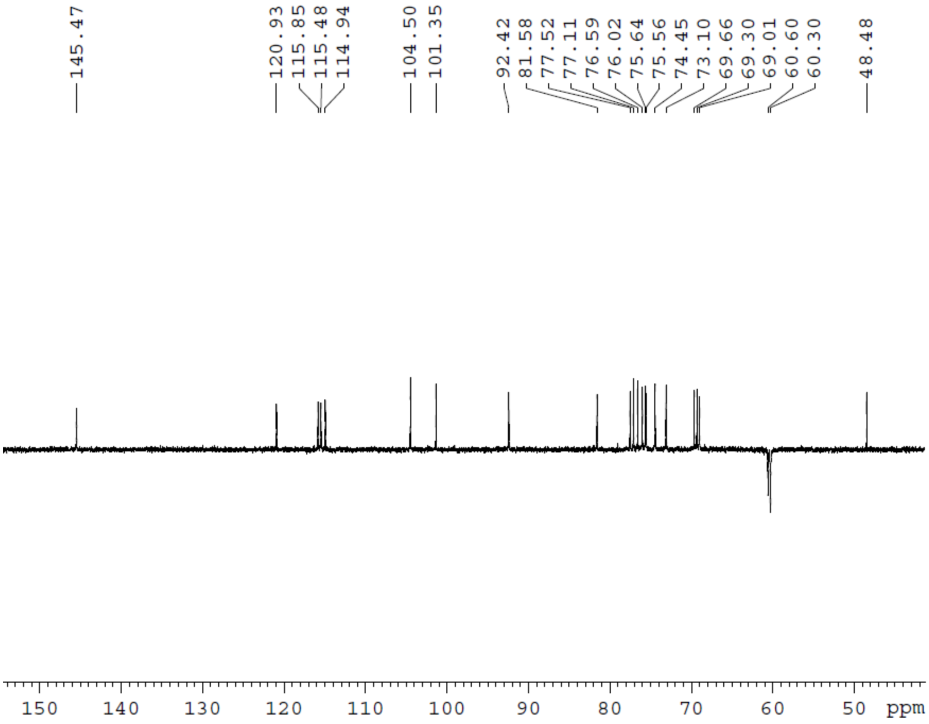

Fig. S61. DEPT 135 (DMSO-d_6) spectrum of compound **9**

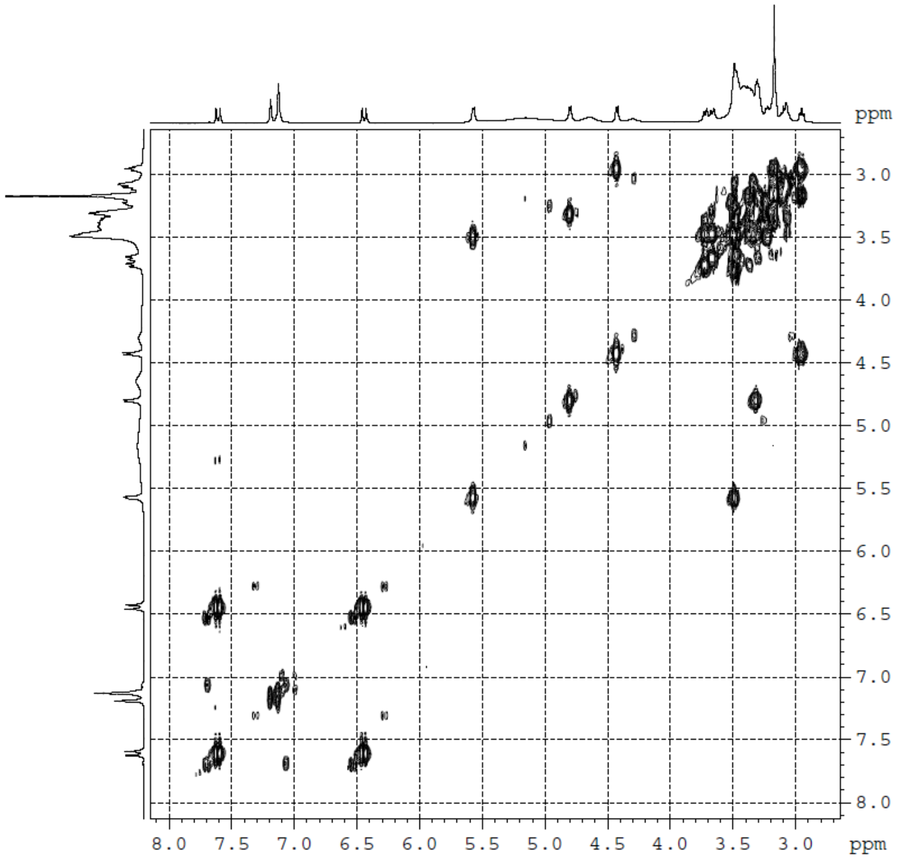

Fig. S62. ^1H ^1H COSY (DMSO-d_6) spectrum of compound **9**

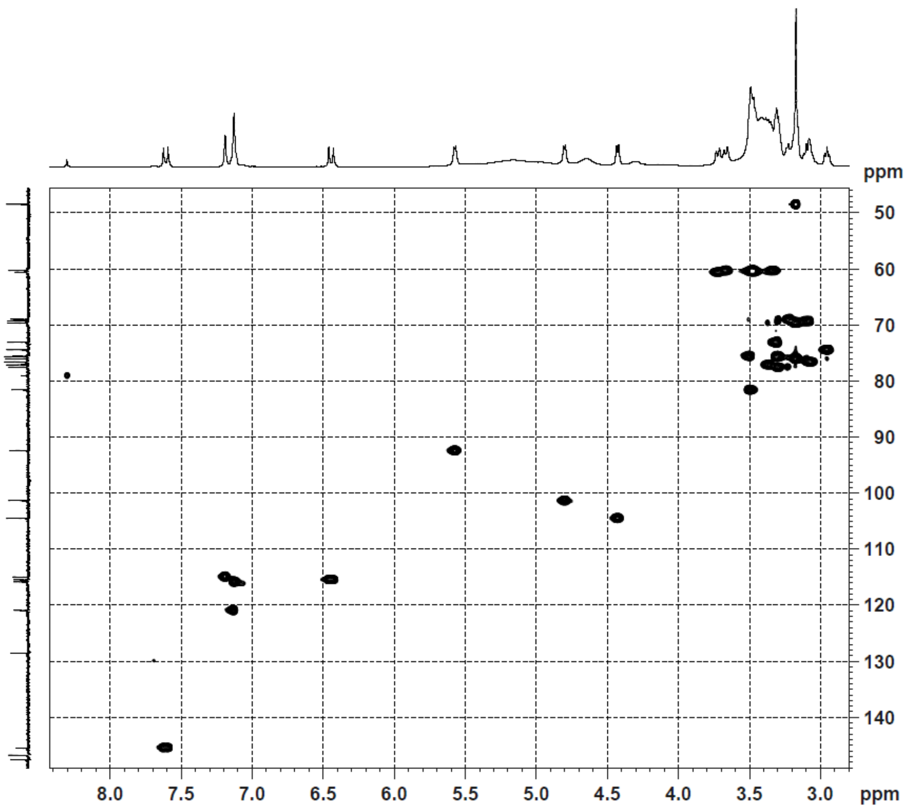

Fig. S63. HSQC (DMSO-d_6) spectrum of compound **9**

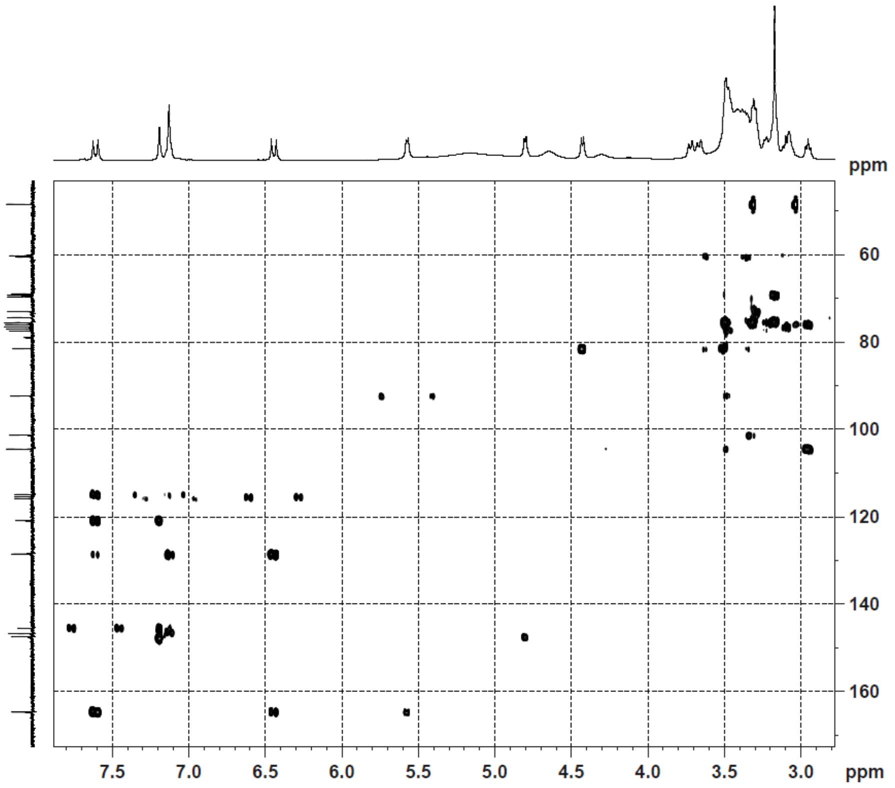

Fig. S64. HMBC (DMSO-d_6) spectrum of compound **9**

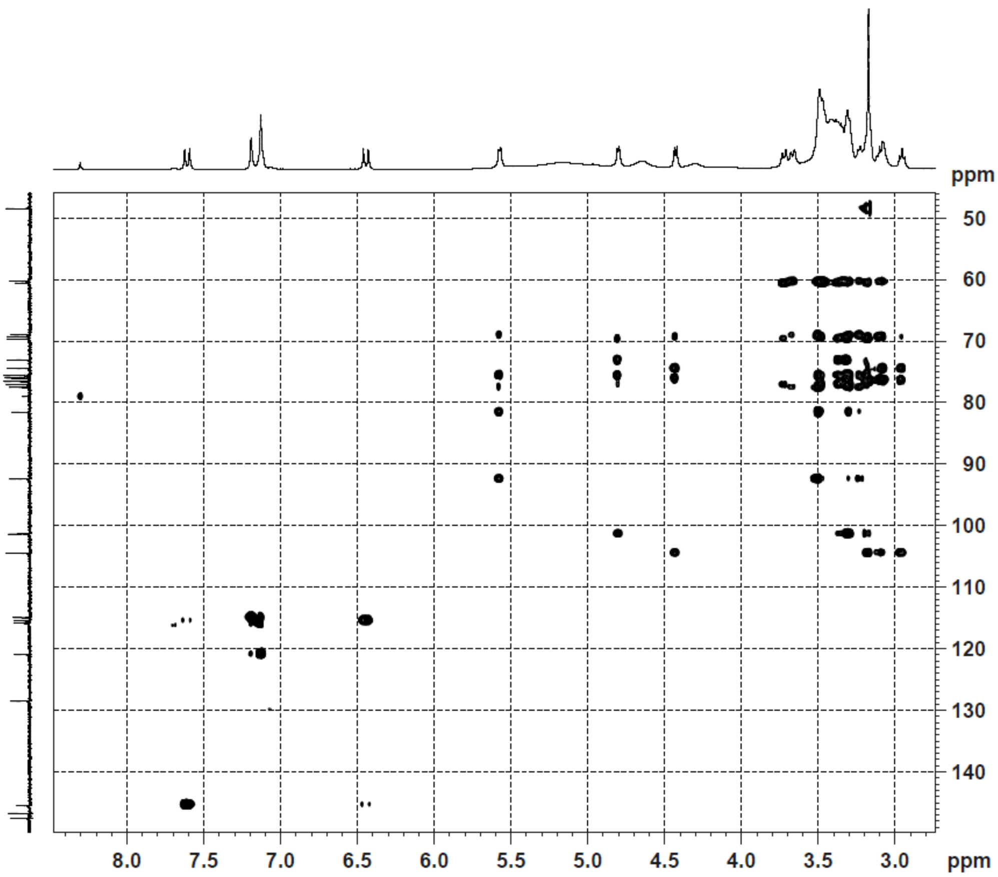

Fig. S65. HSQC-TOCSY (DMSO-*d*6) spectrum of compound **9**

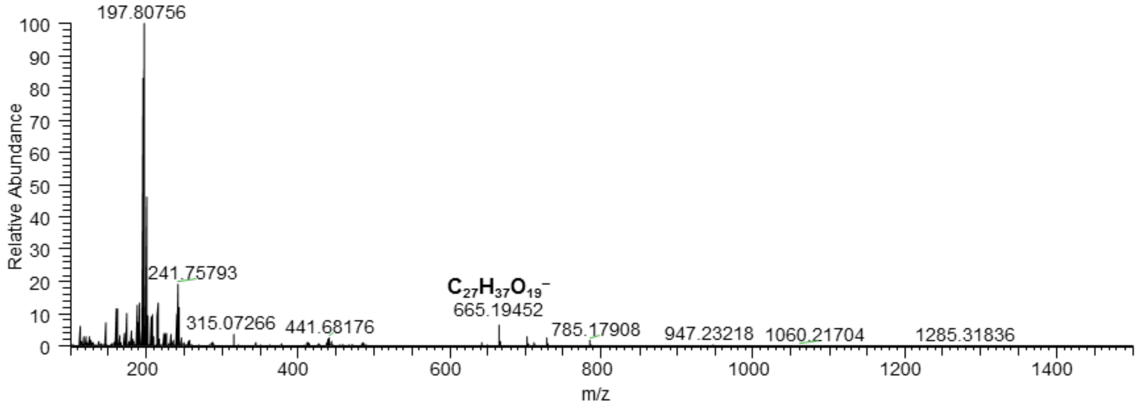

Fig. S66. ESI-Q-Orbitrap MS spectrum of compound **9**

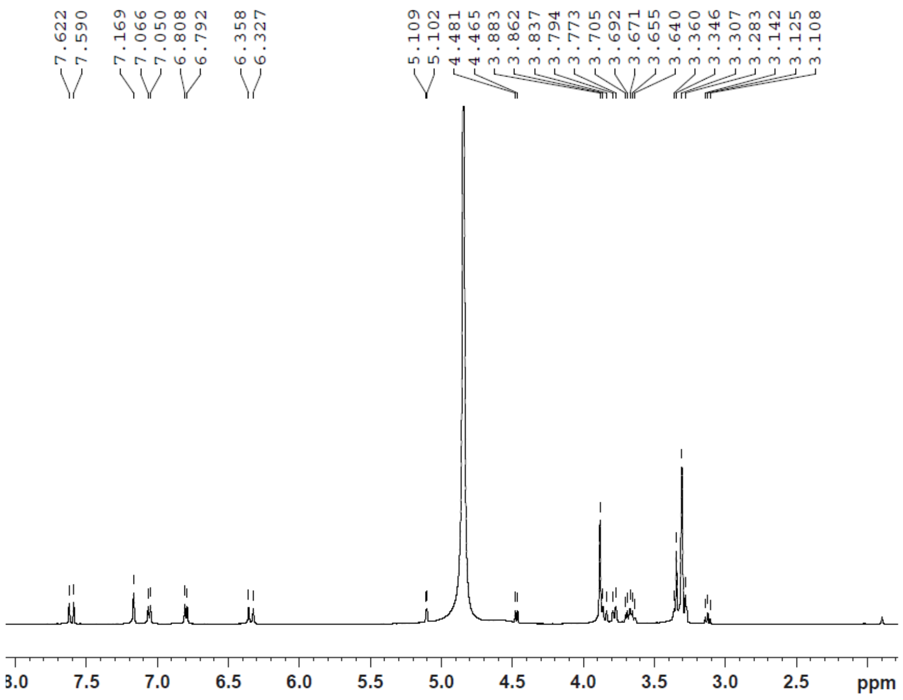

Fig. S67. ^{1}H NMR (500 MHz, CD_3OD) spectrum of compound **10**

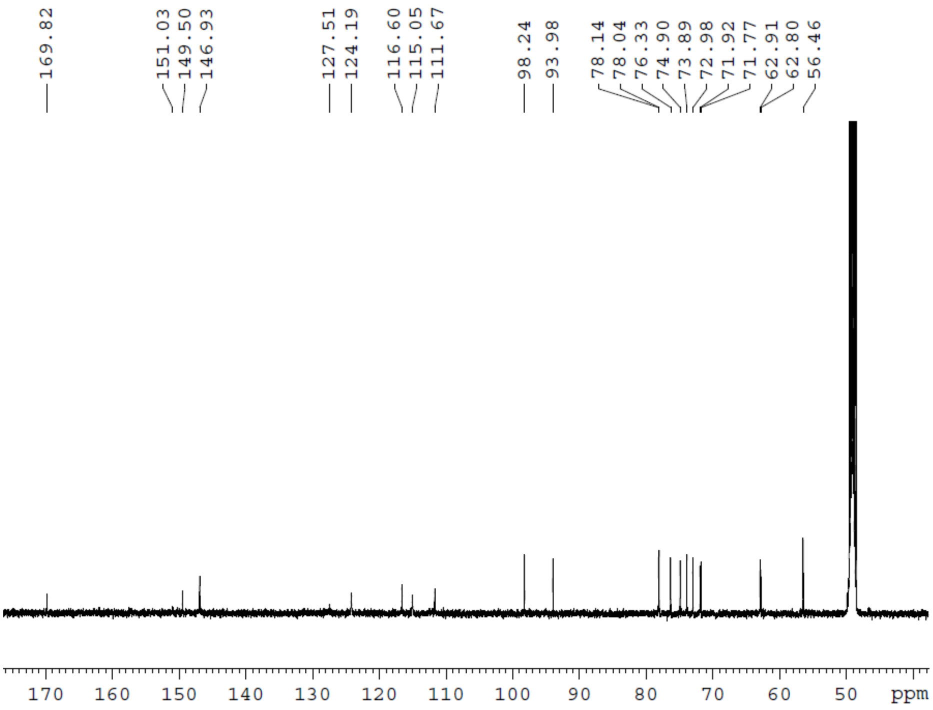

Fig. S68. ^{13}C NMR (125 MHz, CD_3OD) spectrum of compound **10**

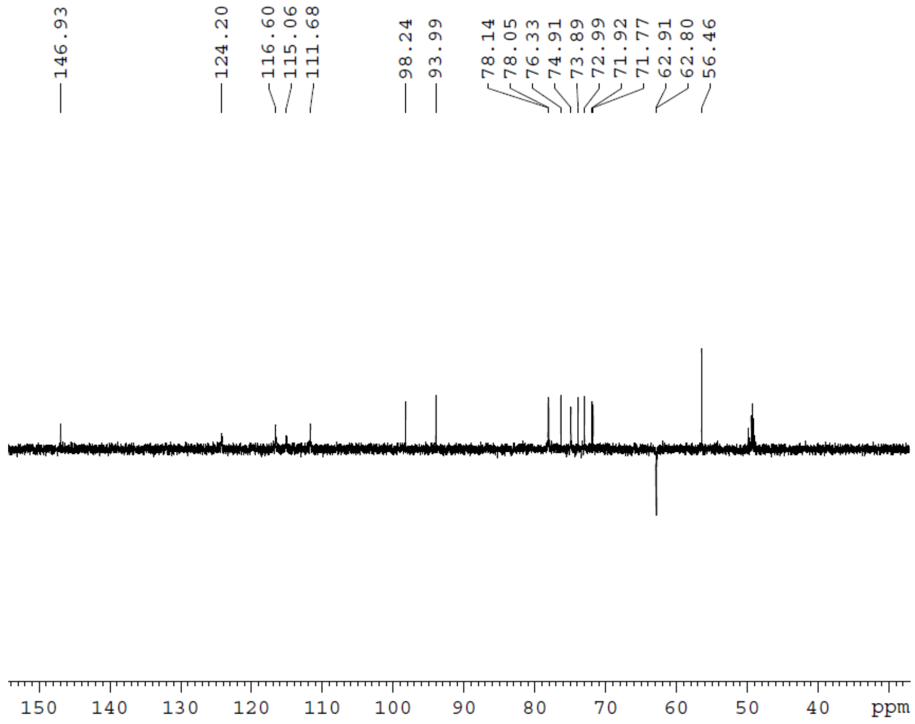

Fig. S69. DEPT 135 (CD3OD) spectrum of compound **10**

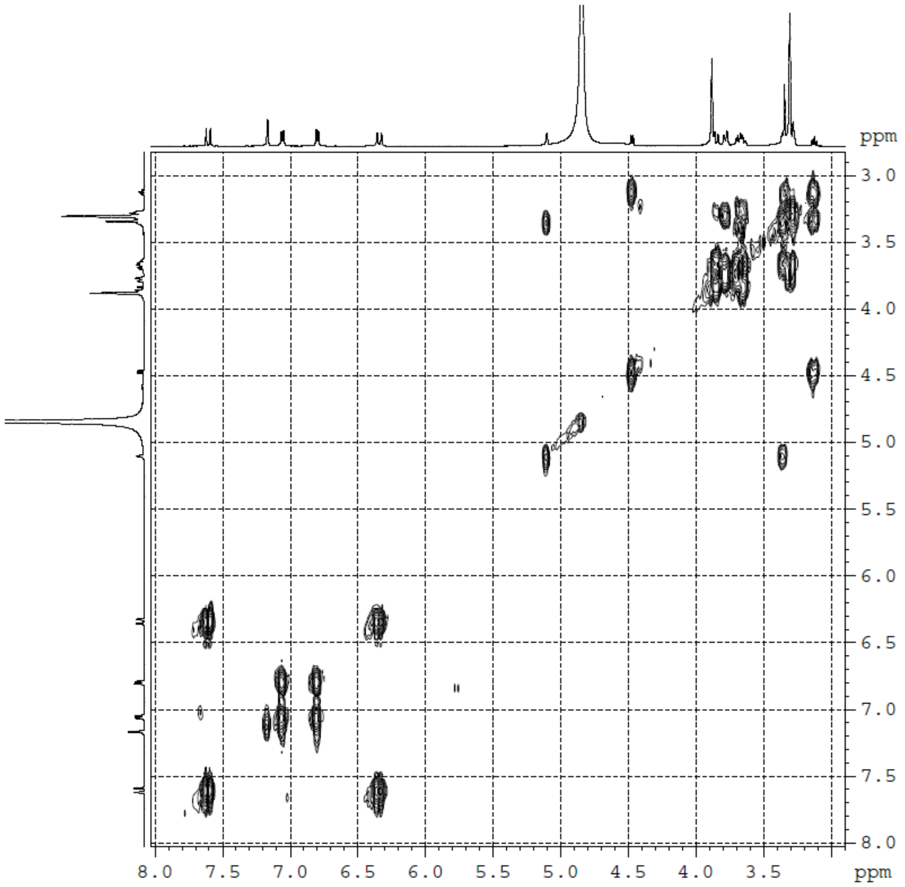

Fig. S70. ^{1}H ^{1}H COSY (CD3OD) spectrum of compound **10**

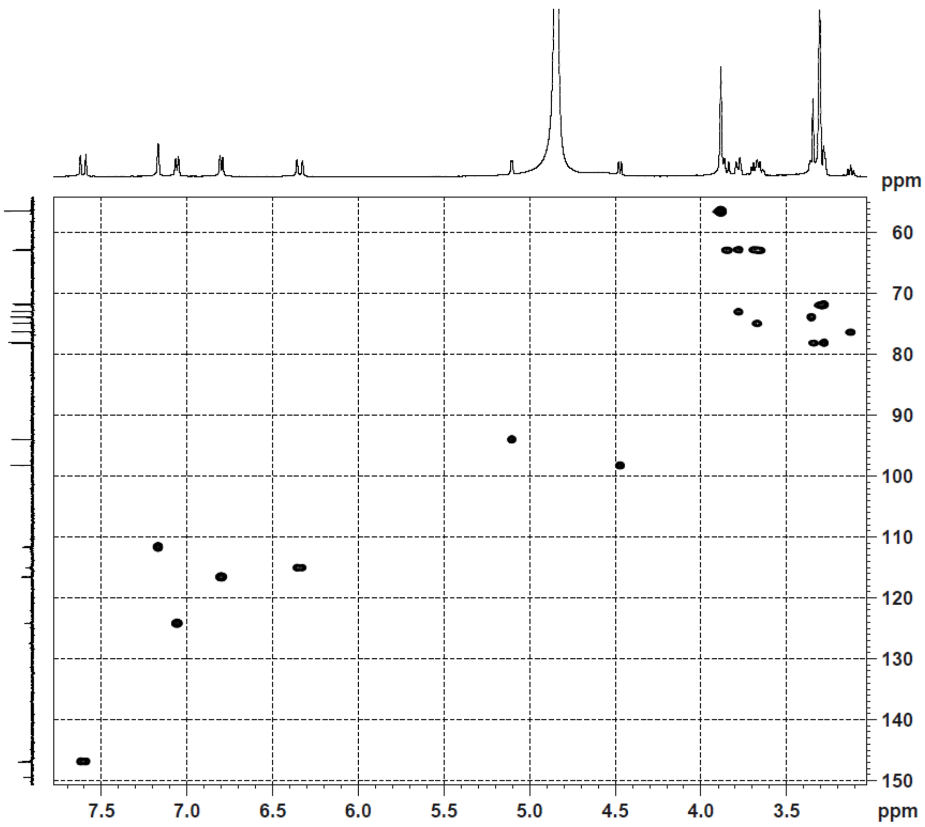

Fig. S71. HSQC (CD_3OD) spectrum of compound **10**

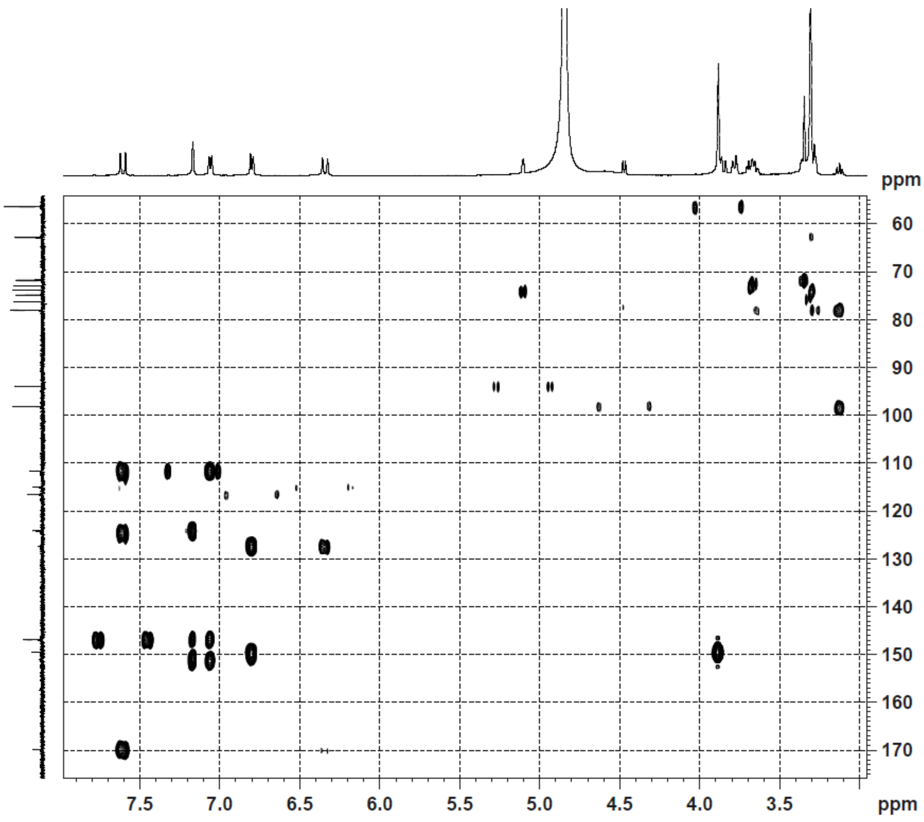

Fig. S72. HMBC (CD_3OD) spectrum of compound **10**

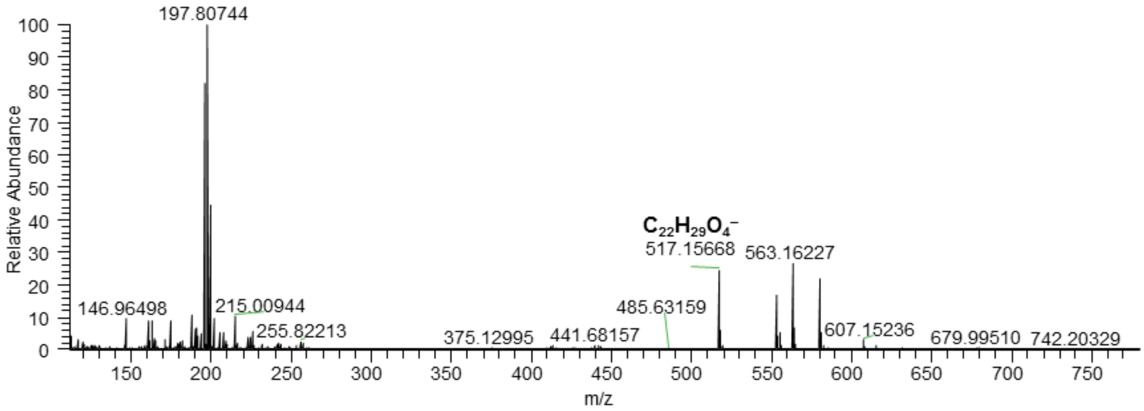

Fig. S73. ESI-Q-Orbitrap MS spectrum of compound **10**

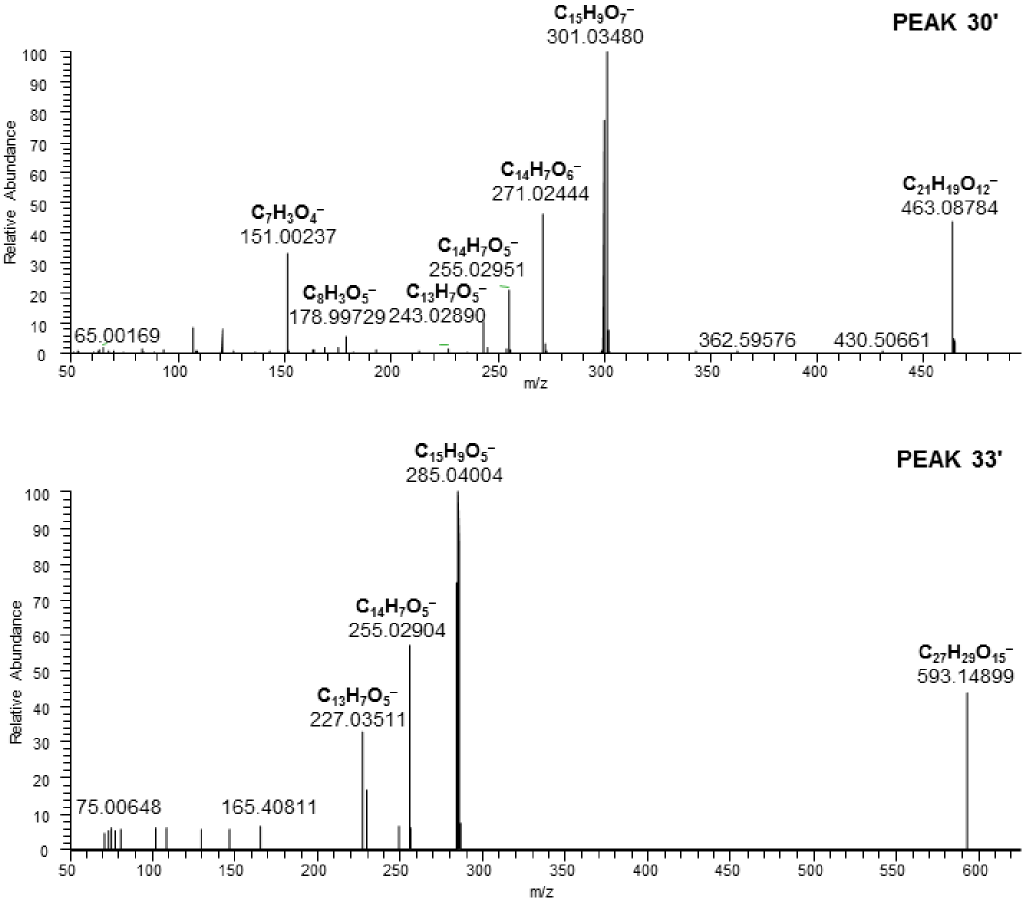

Fig. S74. The tandem MS of the [M−H]⁻ ions for peaks 30' and 33'

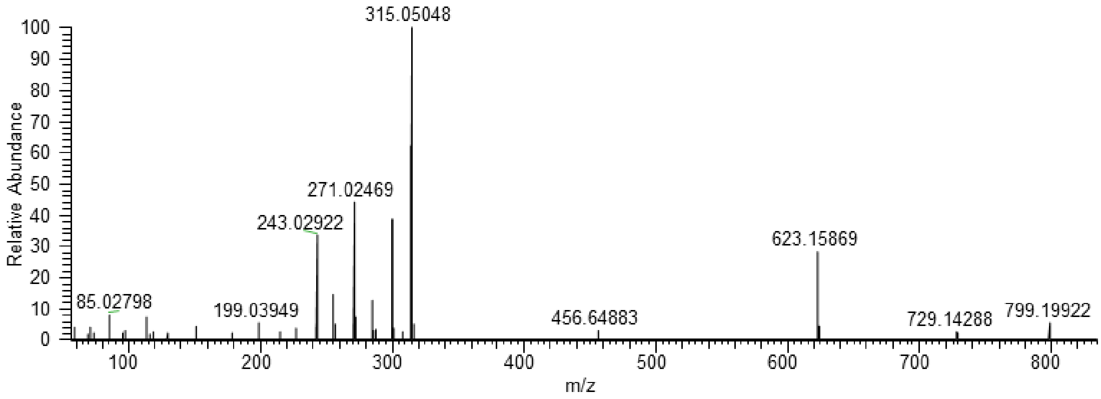

Fig. S75. The tandem MS of the [M−H]⁻ ions for peak 28'

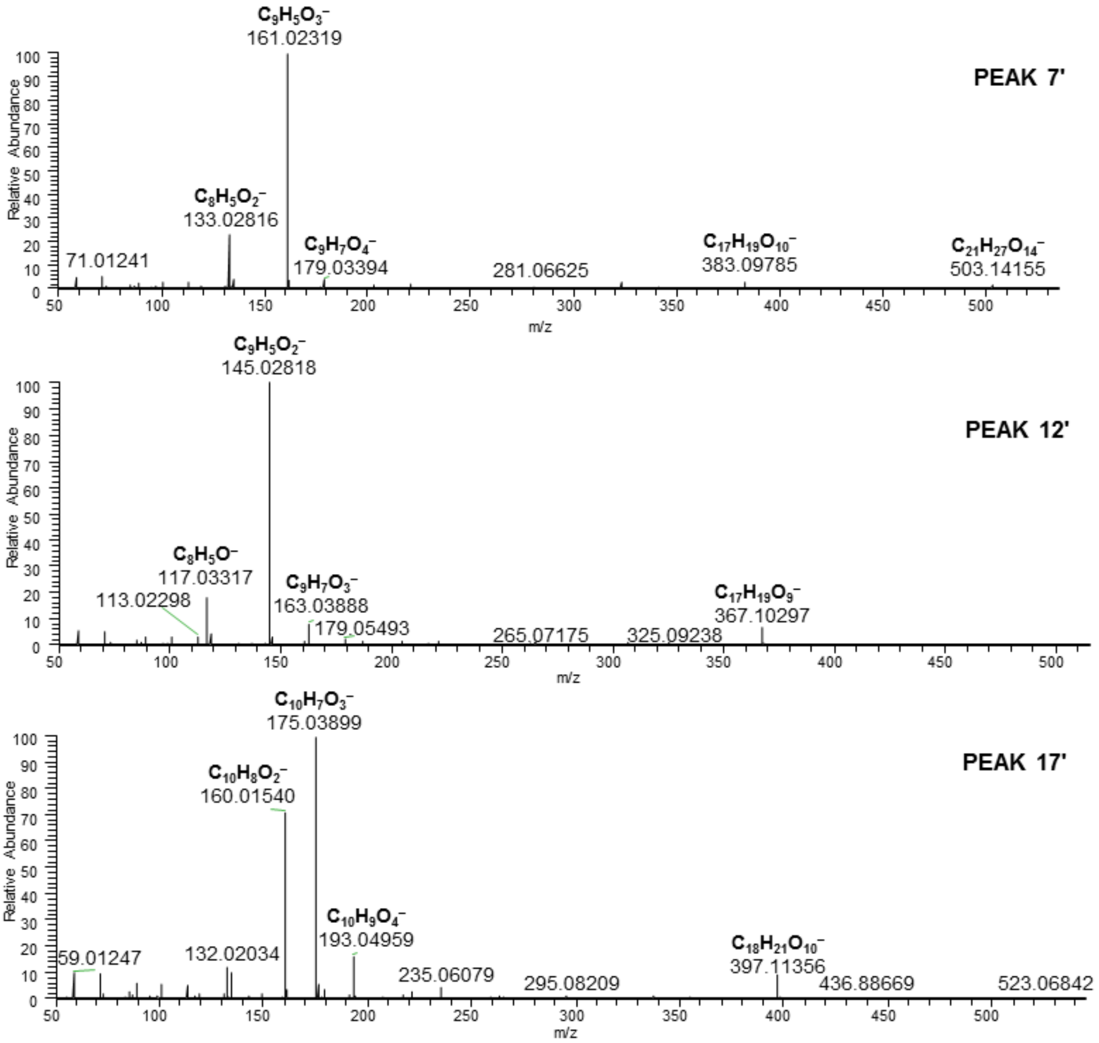

Fig. S76. The MS/MS spectrum of [M–H]$^-$ ions of fragment ions for peaks 7', 12' and 17' by ESI-Q-Orbitrap MS

Extraction and Isolation

The fresh aerial parts of *A. mongolicum* (17.8 kg) was successively heated reflux with 95% EtOH for 3 h and 50% EtOH for 2 h one time each to obtain *A. mongolicum* extract (515.0 g). Then 470.1 g of it was dissolved was 5 L H_2O and partitioned with EtOAc/H_2O (1:1, v/v) three times. As results, EtOAc layer extract (64.9 g) and H_2O layer extract (381.0 g) were yielded, reapectively. H_2O layer extract (342.2 g) was subjected to D101 macroporous CC (H_2O → 95% EtOH → Acetone), and H_2O (289.0 g), 95% EtOH (29.9 g) and acetone (0.5 g) eluates were gained successively.

The 95% EtOH eluate (20.0 g) was isolated by pHPLC [MeOH-H_2O (0–30 min, 20:80, v/v; 30–31 min, 20:80–30:70, v/v; 31–43 min, 30:70, v/v; 43–44 min, 30:70–45:55, v/v; 44–56 min, 45:55, v/v; 56–57 min, 45:55–100:0, v/v; 57–70 min, 100:0, v/v), flow rate: 40 mL/min;] to yield twenty-two fractions (Fr. 1–Fr. 22). Fraction 3 (1.2 g) was separated by pHPLC [CH_3CN-H_2O (0-30 min, 7:93, v/v; 30–31 min, 7:93–10:90, v/v; 31–65 min, 10:90, v/v), flow rate: 40 mL/min], as a result, fourteen fractions (Fr. 3-1–Fr. 3-14) were given. Fraction 3-2 (76.6 mg) was further purified by pHPLC [CH_3CN-H_2O (4:96, v/v), flow rate: 9 mL/min] to gain mongophenoside A_3 (**9**, 8.4 mg; t_R 32.7 min). Fraction 3-5 (78.4 mg) was isolated by pHPLC [CH_3CN-H_2O (7:93, v/v, flow rate: 9 mL/min), and mongoflavonoside B_2 (**6**, 14.6 mg, t_R 31.8 min) was obtained. Fraction 3-6 (124.3 mg) was purified by pHPLC [CH_3CN-H_2O (8:92, v/v), flow rate: 9 mL/min] to afford mongoflavonoside A_1 (**1**, 68.1 mg, t_R 21.5 min). Fraction 3-8 (108.5 mg) was separated by pHPLC [CH_3CN-H_2O (7:93, v/v), flow rate: 9 mL/min], and three fractions (Fr. 3-8-1–Fr. 3-8-3) were producted. Then Fraction 3-8-2 (54.9 mg) was further isolated by pHPLC [MeOH-H_2O (18:82, v/v), flow rate: 9 mL/min] to yield mongoflavonoside A_3 (**3**, 9.7 mg, t_R 29.0 min). Fraction 5 (532.1 mg) was subjected to pHPLC [CH_3CN-H_2O (8:92, v/v), flow rate: 9 mL/min], and eight fractions (Fr. 5-1–Fr. 5-8) were given. Among them, fraction 5-8 was identified as quercetin-3-*O*-*β*-D-rutinoside-7-*O*-*β*-D-glucuronide (**25**, 84.8 mg, t_R 50.7 min). Fraction 5-3 (101.8 mg) was isolated by pHPLC [CH_3CN-H_2O (4:96, v/v), flow rate: 9 mL/min] to obtain mongophenoside A_2 (**8**, 5.5 mg, t_R 32.6 min). Fraction 7 (3.1 g) was separated by pHPLC [gradient program: CH_3CN-H_2O (0-30 min, 8:92, v/v; 30–31 min, 8:92–13:87, v/v; 31–65 min, 13:87, v/v), flow rate: 40 mL/min], and seven frations (Fr. 7-1–Fr. 7-7) were yielded. Fraction 7-1 (167.7 mg) was isolated by pHPLC [CH_3CN-H_2O (8:92, v/v), flow rate: 9 mL/min] to afford quercetin-3,7,4'-tri-*O*-glucoside (**26**, 14.1 mg, t_R 21.6 min). Fraction 7-2 (1.1 g) was subjected to Sephadex LH-20 CC [MeOH-H_2O (1:1, v/v)] and pHPLC [CH_3CN-H_2O (7:93, v/v)] to obtain mongophenoside A_1 (**7**, 141.9 mg, t_R 24.6 min). Fraction 9 (0.2 g) was sunjected to Sephadex LH-20 CC [MeOH-H_2O (50:50, v/v)], and four fractions (Fr. 9-1–Fr. 9-4) were given. Fraction 9-3 (91.2 mg) was purified by pHPLC [CH_3CN-H_2O (12:88, v/v), flow rate: 9 mL/min] to yield kaempferol-3,7,4'-tri-*O*-*β*-glucoside (**18**, 20.9 mg, t_R 8.6 min). Fraction 11 (0.6 g) was isolated by Sephadex LH-20 CC [MeOH-H_2O (1:1, v/v)] to give five fractions (Fr. 11-1–Fr. 11-5). Fraction 11-4 (338.1 mg) was isolated by pHPLC [CH_3CN-H_2O (10:90, v/v), flow rate: 9 mL/min] to give *p*-hydroxycinnamate sophorose (**28**, 205.8 mg, t_R 20.7 min). Fraction 12 (1.1 g) was subjected to Sephadex LH-20 CC [MeOH-H_2O (1:1, v/v)], and four fractions (Fr. 12-1–Fr. 12-4) were obtgained. Fraction 12-3 (88.3 mg) was purified by pHPLC [CH_3CN-H_2O (11:89, v/v), flow rate: 9 mL/min] to yield mongoflavonoside A_4 (**4**, 4.6 mg, t_R 26.9 min). Fraction 12-4 (912.8 mg) was separated by pHPLC [CH_3CN-H_2O (11:89, v/v), flow rate: 9 mL/min] to product tuberonoid A (**29**, 125.8 mg, t_R 20.6 min). Fraction 13 (0.5 g) was isolated by Sephadex LH-20 CC [MeOH-H_2O (1:1, v/v)], and four fractions (Fr. 13-1–Fr. 13-4) were given. Fraction 13-2 (389.9 mg) was further separated by pHPLC [CH_3CN-H_2O (9:91, v/v), flow rate: 9 mL/min] to gain six fractions (Fr. 13-2-1–Fr. 13-2-6). Fraction 13-2-3 (29.9 mg) was separated by pHPLC [CH_3CN-H_2O (11:89, v/v), flow rate: 9 mL/min] to give mongophenoside B (**10**, 5.4 mg; t_R 26.0 min). Fraction 14 (0.7 g) was isoalted by pHPLC [CH_3CN-H_2O (11:89, v/v), flow rate: 9 mL/min], and nine fractions (Fr. 14-1–Fr. 14-9) were given. Fraction 14-5 (48.1 mg) was further purified by pHPLC [CH_3CN-H_2O (10:90, v/v), flow rate: 9 mL/min] to gain benzyl-*O*-*β*-D-glucopyranoside (**31**, 11.9 mg, t_R 29.4 min). Fraction 14-7 (102.7 mg) was subjected to pHPLC [CH_3CN-H_2O (10:90, v/v), flow rate: 9 mL/min] to yield *trans*-caffeic acid (**30**, 15.5 mg, t_R 32.4 min). Fraction 16 (3.2 g) was separated by Sephadex LH-20 CC [MeOH-H_2O (1:1, v/v)], and five fractions (Fr. 16-1–Fr. 16-5) were producted. Fraction 16-2 (402.9 mg) was further isolated by pHPLC

[CH_3CN-H_2O (16:84, v/v), flow rate: 9 mL/min] to give five fractions (Fr. 16-2-1–Fr. 16-2-5). Fraction 16-2-4 (49.5 mg) was purified by pHPLC [CH_3CN-H_2O (19:81, v/v), flow rate: 9 mL/min], and mongoflavonoside A$_2$ (**2**, 35.3 mg, t_R 11.3 min) was yielded. Fraction 18 (1.4 g) was subjected to Sephadex LH-20 CC (MeOH), and pHPLC [CH_3CN-H_2O (20:80, v/v), flow rate: 9 mL/min] to yield mongoflavonoside B$_1$ (**5**, 8.3 mg, t_R 20.3 min). Fraction 19 (1.1 g) was isolated by Sephadex LH-20 CC (MeOH) and pHPLC [CH_3CN-H_2O (22:78, v/v), flow rate: 9 mL/min] to give isorhamnetin 3-*O*-*β*-D-glucopyranoside (**27**, 6.0 mg, t_R 24.3 min). All the separations were conducted at the room temperature.

Mongoflavonoside A$_1$ (**1**): Yellow powder; $[\alpha]_D^{25}$ –54.0 (*c* = 0.30, H_2O); IR ν_{max} (KBr) cm^{-1}: 3354, 2921, 2852, 1716, 1699, 1652, 1601, 1507, 1457, 1397, 1248, 1209, 1180, 1072, 1023; UV λ_{max} (MeOH) nm (log ε): 265 (4.15), 330 (3.96); ^{1}H NMR (DMSO-d_6, 500 MHz) spectroscopic data: δ 6.48 (1H, br. s, H-6), 6.88 (1H, br. s, H-8), 8.16 (2H, d, *J* = 9.0 Hz, H-2',6'), 7.19 (2H, d, *J* = 9.0 Hz, H-3',5'), 5.50 (1H, d, *J* = 7.0 Hz, H-1''), 3.20 (1H, dd, *J* = 7.0, 8.0 Hz, H-2''), 3.23 (1H, dd, *J* = 8.0, 8.0 Hz, H-3''), 3.10 (1H, dd, *J* = 8.0, 8.0, H-4''), 3.11 (1H, m, H-5''), [3.35 (1H, dd, *J* = 6.5, 11.0 Hz), 3.58 (1H, br. d, *ca.* *J* = 11 Hz), H$_2$-6''], 5.26 (1H, d, *J* = 6.5, H-1'''), 3.32 (1H, dd, *J* = 6.5, 7.5 Hz, H-2'''), 3.36 (1H, dd, *J* = 7.5, 8.5 Hz, H-3'''), 3.37 (1H, dd, *J* = 8.5, 8.5 Hz, H-4'''), 3.98 (1H, d, *J* = 8.5 Hz, H-5'''), 5.05 (1H, d, *J* = 7.0 Hz, H-1''''), 3.31 (1H, dd, *J* = 7.0, 7.5 Hz, H-2''''), 3.33 (1H, dd, *J* = 7.5, 8.5 Hz, H-3''''), 3.21 (1H, dd, *J* = 7.0, 8.5, H-4''''), 3.42 (1H, m, H-5''''), [3.50 (1H, dd, *J* = 5.5, 11.0 Hz), 3.72 (1H, br. d, *ca.* *J* = 11 Hz), H$_2$-6''''], 12.57 (1H, br. s, 5-OH); ^{13}C NMR (DMSO-d_6, 125 MHz) spectroscopic data, see Table 1. ESI-Q-Orbitrap MS: Negative-ion mode *m/z* 785.17883 [M – H]$^-$ (calcd for $C_{33}H_{37}O_{22}$, 785.17710).

Mongoflavonoside A$_2$ (**2**): Yellow powder; $[\alpha]_D^{25}$ –26.0 (*c* = 0.97, MeOH); IR ν_{max} (KBr) cm^{-1}: 3367, 2923, 2891, 1655, 1606, 1503, 1454, 1423, 1363, 1306, 1246, 1208, 1183, 1072, 1022; UV λ_{max} (MeOH) nm (log ε): 267 (4.31), 351 (4.10); ^{1}H NMR (DMSO-d_6, 500 MHz) spectroscopic data: δ 6.21 (1H, br. s, H-6), 6.44 (1H, br. s, H-8), 8.11 (2H, d, *J* = 9.0 Hz, H-2',6'), 7.17 (2H, d, *J* = 9.0 Hz, H-3',5'), 5.51 (1H, d, *J* = 8.0 Hz, H-1''), 3.26 (1H, dd, *J* = 8.0, 8.5 Hz, H-2''), 3.41 (1H, m, overlapped, H-3''), 3.39 (1H, dd, *J* = 9.0, 9.0 Hz, H-4''), 3.28 (1H, m, H-5''), [3.51 (1H, dd, *J* = 5.5, 11.0 Hz), 3.63 (1H, br. d, *ca.* *J* = 11 Hz), H$_2$-6''], 4.27 (1H, d, *J* = 8.0 Hz, H-1'''), 3.00 (1H, dd, *J* = 8.0, 8.5 Hz, H-2'''), 3.16 (1H, dd, *J* = 8.5, 9.0 Hz, H-3'''), 3.06 (1H, dd, *J* = 9.0, 9.0 Hz, H-4'''), 3.22 (1H, m, H-5'''), [3.42 (1H, m, overlapped), 3.71 (1H, m, overlapped), H$_2$-6'''], 5.03 (1H, d, *J* = 7.5 Hz, H-1''''), 3.29 (1H, dd, *J* = 7.5, 8.5 Hz, H-2''''), 3.32 (1H, dd, *J* = 8.5, 9.0 Hz, H-3''''), 3.21 (1H, dd, *J* = 9.0, 9.0 Hz, H-4''''), 3.41 (1H, m, overlapped, H-5''''), [3.50 (1H, dd, *J* = 5.5, 11.0 Hz), 3.71 (1H, m, overlapped), H$_2$-6''''], 12.54 (1H, br. s, 5-OH); ^{13}C NMR (DMSO-d_6, 125 MHz) spectroscopic data, see Table 1. ESI-Q-Orbitrap MS: Negative-ion mode *m/z* 771.19971 [M – H]$^-$ (calcd for $C_{33}H_{39}O_{21}$, 771.19783).

Mongoflavonoside A$_3$ (**3**): Yellow powder; $[\alpha]_D^{25}$ –64.7 (*c* = 0.22, H_2O); IR ν_{max} (KBr) cm^{-1}: 3365, 2917, 2890, 1652, 1602, 1569, 1489, 1418, 1348, 1297, 1247, 1210, 1182, 1070, 1021; UV λ_{max} (H_2O) nm (log ε): 261 (3.73), 315 (3.54), 343 (3.50, sh); ^{1}H NMR (DMSO-d_6, 500 MHz) spectroscopic data: δ 6.46 (1H, br. s, H-6), 6.85 (1H, br. s, H-8), 8.14 (2H, d, *J* = 8.5 Hz, H-2',6'), 7.18 (2H, d, *J* = 8.5 Hz, H-3',5'), 5.53 (1H, d, *J* = 7.5 Hz, H-1''), 3.25 (1H, dd, *J* = 7.5, 8.0 Hz, H-2''), 3.40 (1H, m, overlapped, H-3''), 3.37 (1H, m, H-4''), 3.28 (1H, m, overlapped, H-5''), [3.51 (1H, dd, *J* = 5.5, 11.0 Hz), 3.63 (1H, br. d, *ca.* *J* = 11 Hz), H$_2$-6''], 4.26 (1H, d, *J* = 7.0 Hz, H-1'''), 2.99 (1H, dd, *J* = 7.0, 8.5 Hz, H-2'''), 3.15 (1H, dd, *J* = 8.5, 9.0 Hz, H-3'''), 3.05 (1H, dd, *J* = 9.0, 9.0 Hz, H-4'''), 3.19 (1H, m, H-5'''), [3.41 (1H, m, overlapped), 3.71 (1H, br. d, *ca.* *J* = 11 Hz), H$_2$-6'''], 5.12 (1H, d, *J* = 7.0 Hz, H-1''''), 3.25 (1H, dd, *J* = 7.0, 7.5 Hz, H-2''''), 3.28 (1H, m, overlapped, H-3''''), 3.22 (1H, dd, *J* = 8.5, 8.5 Hz, H-4''''), 3.70 (1H, d, *J* = 8.5 Hz, H-5''''), 5.04 (1H, d, *J* = 7.0 Hz, H-1'''''), 3.29 (1H, dd, *J* = 7.0, 7.5 Hz, H-2'''''), 3.28 (1H, m, overlapped, H-3'''''), 3.19 (1H, m, H-4'''''), 3.41 (1H, m, overlapped, H-5'''''), [3.49 (1H, dd, *J* = 6.0, 11.0 Hz), 3.70 (1H, br. d, *ca.* *J* = 11 Hz), H$_2$-6'''''], 12.56 (1H, br. s, 5-OH); ^{13}C NMR (DMSO-d_6, 125 MHz) spectroscopic data, see Table 1. ESI-Q-Orbitrap MS: Negative-ion mode *m/z* 947.23242 [M – H]$^-$ (calcd for $C_{39}H_{47}O_{27}$, 947.22992).

Mongoflavonoside A$_4$ (**4**): Yellow powder; $[\alpha]_D^{25}$ –45.6 (*c* = 0.22, H_2O); IR ν_{max} (KBr) cm^{-1}: 3366, 2920, 2888, 1650, 1602, 1500, 1452, 1421, 1359, 1299, 1235, 1202, 1175, 1066, 1018; UV λ_{max} (H_2O) nm (log ε): 265 (4.24), 272 (4.12, sh), 342 (4.11); ^{1}H NMR (DMSO-d_6, 500 MHz) spectroscopic data: δ 6.37 (1H, br. s, H-6), 6.73 (1H, br. s, H-8), 8.00 (2H, d, *J* = 9.0 Hz, H-2',6'), 6.88 (2H, d, *J* = 9.0 Hz, H-3',5'), 5.23 (1H, d, *J*

= 7.5 Hz, H-1''), 3.24 (1H, dd, *J* = 7.5, 8.0 Hz, H-2''), 3.38 (1H, m, overlapped, H-3''), 3.20 (1H, m, overlapped, H-4''), 3.45 (1H, m, H-5''), [3.37 (1H, m, overlapped), 3.81 (1H, br. d, *ca*. *J* = 11 Hz), H_2-6''], 4.14 (1H, d, *J* = 7.5 Hz, H-1'''), 2.96 (1H, dd, *J* = 7.5, 8.0 Hz, H-2'''), 3.13 (1H, m, H-3'''), 3.04 (1H, dd, *J* = 9.0, 9.0 Hz, H-4'''), 3.19 (1H, m, overlapped, H-5'''), [3.41 (1H, dd, *J* = 5.0, 11.0 Hz), 3.69 (1H, br. d, *ca*. *J* = 11 Hz), H_2-6'''], 4.40 (1H, br. s, H-1''''), 3.31 (1H, m, H-2''''), 3.20 (1H, m, overlapped, H-3''''), 3.05 (1H, m, H-4''''), 3.16 (1H, m, H-5''''), 0.90 (3H, d, *J* = 6.0 Hz, H_3-6''''), 5.16 (1H, d, *J* = 7.0 Hz, H-1'''''), 3.26 (1H, m, H-2'''''), 3.28 (1H, m, overlapped, H-3'''''), 3.22 (1H, dd, *J* = 8.5, 8.5 Hz, H-4'''''), 3.72 (1H, d, *J* = 8.5 Hz, H-5'''''), 12.58 (1H, br. s, 5-OH); ^{13}C NMR (DMSO-d_6, 125 MHz) spectroscopic data, see Table 1. ESI-Q-Orbitrap MS: Negative-ion mode *m*/*z* 931.23785 [M – H]$^-$ (calcd for $C_{39}H_{47}O_{26}$, 931.23501).

Mongoflavonoside B_1 (**5**): Yellow powder; $[\alpha]_D^{25}$ –12.0 (*c* = 0.30, MeOH); IR ν_{max} (KBr) cm^{-1}: 3362, 2921, 2619, 1721, 1654, 1605, 1507, 1448, 1361, 1299, 1271, 1201, 1169, 1070, 1035; UV λ_{max} (MeOH) nm (log ε): 257 (4.30), 270 (4.20, sh); 356 (4.19); ^{1}H NMR (DMSO-d_6, 500 MHz) spectroscopic data: δ 6.18 (1H, br. s, H-6), 6.39 (1H, br. s, H-8), 7.51 (1H, d, *J* = 2.0 Hz, H-2'), 6.83 (1H, d, *J* = 8.5 Hz, H-5'), 7.50 (1H, dd, *J* = 2.0, 8.5 Hz, H-6'), 5.40 (1H, d, *J* = 8.0 Hz, H-1''), 3.36 (1H, dd, *J* = 8.0, 9.0 Hz, H-2''), 3.43 (1H, dd, *J* = 9.0, 9.0 Hz, H-3''), 3.42 (1H, dd, *J* = 9.0, 9.0 Hz, H-4''), 3.51 (1H, m, H-5''), [4.12 (1H, dd, *J* = 6.0, 12.0 Hz), 4.22 (1H, br. d, *ca*. *J* = 12 Hz), H_2-6''], 1.71 (3H, s, 6''-$COCH_3$), 4.21 (1H, d, *J* = 8.0 Hz, H-1'''), 2.97 (1H, dd, *J* = 8.0, 8.5 Hz, H-2'''), 3.14 (1H, dd, *J* = 8.0, 9.0 Hz, H-3'''), 3.05 (1H, dd, *J* = 9.0, 9.0 Hz, H-4'''), 3.22 (1H, m, H-5'''), [3.41 (1H, m), 3.71 (1H, br. d, *ca*. *J* = 10 Hz), H_2-6'''], 12.49 (1H, br. s, 5-OH); ^{13}C NMR (DMSO-d_6, 125 MHz) spectroscopic data, see Table 1. ESI-Q-Orbitrap MS: Negative-ion mode *m*/*z* 667.15228 [M – H]$^-$ (calcd for $C_{29}H_{31}O_{18}$, 667.15049).

Mongoflavonoside B_2 (**6**): Yellow powder; $[\alpha]_D^{25}$ –78.0 (*c* = 0.32, H_2O); IR ν_{max} (KBr) cm^{-1}: 3362, 2921, 2619, 1716, 1653, 1602, 1506, 1450, 1361, 1299, 1271, 1201, 1169, 1066, 1035; UV λ_{max} (H_2O) nm (log ε): 253 (4.18, sh), 265 (4.20), 341 (4.04). ^{1}H NMR (DMSO-d_6, 500 MHz) spectroscopic data: δ 6.45 (1H, br. s, H-6), 6.85 (1H, br. s, H-8), 7.69 (1H, d, *J* = 1.5 Hz, H-2'), 7.23 (1H, d, *J* = 8.5 Hz, H-5'), 7.64 (1H, dd, *J* = 1.5, 8.5 Hz, H-6'), 5.52 (1H, d, *J* = 7.0 Hz, H-1''), 3.22 (1H, m, overlapped, H-2''), 3.22 (1H, m, overlapped, H-3''), 3.08 (1H, dd, *J* = 8.0, 8.0 Hz, H-4''), 3.09 (1H, m, H-5''), [3.33 (1H, dd, *J* = 5.0, 12.0 Hz), 3.58 (1H, br. d, *ca*. *J* = 12 Hz), H_2-6''], 5.16 (1H, d, *J* = 7.0 Hz, H-1'''), 3.27 (1H, dd, *J* = 7.0, 9.0 Hz, H-2'''), 3.29 (1H, dd, *J* = 9.0, 9.0 Hz, H-3'''), 3.26 (1H, dd, *J* = 8.0, 9.0 Hz, H-4'''), 3.76 (1H, d, *J* = 8.0 Hz, H-5'''), 4.88 (1H, d, *J* = 7.0 Hz, H-1''''), 3.33 (1H, m, overlapped, H-2''''), 3.32 (1H, m, overlapped, H-3''''), 3.20 (1H, dd, *J* = 9.0, 9.0 Hz, H-4''''), 3.40 (1H, m, H-5''''), [3.49 (1H, dd, *J* = 5.0, 11.5 Hz), 3.73 (1H, br. d, *ca*. *J* = 12 Hz), H_2-6''''], 12.63 (1H, br. s, 5-OH); ^{13}C NMR (DMSO-d_6, 125 MHz) spectroscopic data, see Table 1. ESI-Q-Orbitrap MS: Positive-ion mode *m*/*z* 801.17407 [M – H]$^-$ (calcd for $C_{33}H_{37}O_{23}$, 801.17201).

Mongophenoside A_1 (**7**): White powder; $[\alpha]_D^{25}$ –21.0 (*c* = 0.79, MeOH); IR ν_{max} (KBr) cm^{-1}: 3362, 2923, 1709, 1628, 1601, 1521, 1447, 1367, 1259, 1168, 1074, 1025; UV λ_{max} (H_2O) nm (log ε): 246 (3.81), 298 (3.89), 333 (4.04); ^{1}H (DMSO-d_6, 500 MHz) spectroscopic data: δ 7.06 (1H, br. s, H-2), 6.75 (1H, d, *J* = 8.0 Hz, H-5), 7.01 (1H, br. d, *ca*. *J* = 8 Hz, H-6), 7.55 (1H, d, *J* = 16.0 Hz, H-7), 6.27 (1H, d, *J* = 16.0 Hz, H-8), 5.56 (1H, d, *J* = 8.0 Hz, H-1'), 3.49 (1H, dd, *J* = 7.5, 8.0 Hz, H-2'), 3.48 (1H, m, overlapped, H-3'), 3.23 (1H, dd, *J* = 8.0, 8.0 Hz, H-4'), 3.28 (1H, m, H-5'), [3.45 (1H, dd, *J* = 4.5, 11.5 Hz), 3.67 (1H, br. d, *ca*. *J* = 12 Hz), H_2-6'], 4.42 (1H, d, *J* = 8.0 Hz, H-1''), 2.95 (1H, dd, *J* = 8.0, 8.5 Hz, H-2''), 3.16 (1H, dd, *J* = 8.5, 9.0 Hz, H-3''), 3.11 (1H, dd, *J* = 9.0, 9.5 Hz, H-4''), 3.06 (1H, m, H-5''), [3.35 (1H, dd, *J* = 4.0, 11.5 Hz), 3.48 (1H, m, overlapped), H_2-6'']; ^{13}C NMR (DMSO-d_6, 125 MHz) spectroscopic data, see Table 2. ESI-Q-Orbitrap MS: Negative-ion mode *m*/*z* 503.14151 [M – H]$^-$ (calcd for $C_{21}H_{27}O_{14}$, 503.13953).

Mongophenoside A_2 (**8**): White powder; $[\alpha]_D^{25}$ –14.5 (*c* = 0.28, MeOH); IR ν_{max} (KBr) cm^{-1}: 3356, 2920, 2880, 1716, 1706, 1647, 1600, 1515, 1456, 1300, 1259, 1164, 1073, 1024; UV λ_{max} (MeOH) nm (log ε): 246 (3.85), 300 (3.97), 329 (4.07); ^{1}H (DMSO-d_6, 500 MHz) spectroscopic data: δ 7.06 (1H, br. s, H-2), 6.76 (1H, d, *J* = 7.5 Hz, H-5), 7.02 (1H, br. d, *ca*. *J* = 8 Hz, H-6), 7.55 (1H, d, *J* = 16.0 Hz, H-7), 6.27 (1H, d, *J* = 16.0 Hz, H-8), 5.56 (1H, d, *J* = 7.0 Hz, H-1'), 3.50 (1H, m, overlapped, H-2'), 3.50 (1H, m, overlapped, H-3'), 3.33 (1H, m, H-4'), 3.48 (1H, m, H-5'), [3.59 (1H, br. d, *ca*. *J* = 12 Hz), 3.99 (1H, br. d, *ca*. *J* = 12 Hz), H_2-6'], 4.42 (1H, d, *J* = 7.5 Hz, H-1''), 2.94 (1H, dd, *J* = 7.5, 8.0 Hz, H-2''), 3.15 (1H, dd, *J* = 8.0, 9.0 Hz, H-3''), 3.11 (1H, dd, *J* = 9.0, 9.0 Hz, H-4''), 3.05 (1H, m, overlapped, H-5''), [3.34 (1H, m), 3.49 (1H, br. d, *ca*. *J* = 10 Hz), H_2-6''], 4.17 (1H, d, *J* = 7.5 Hz, H-1'''), 2.95 (1H, dd, *J* = 7.5, 8.0 Hz, H-2'''), 3.12 (1H, dd, *J* =

8.0, 9.0 Hz, H-3'''), 3.05 (1H, m, overlapped, H-4'''), 3.06 (1H, m, H-5'''), [3.42 (1H, m), 3.64 (1H, br. d, *ca.* *J* = 11 Hz), H_2-6''']; ^{13}C NMR (DMSO-d_6, 125 MHz) spectroscopic data, see Table 2. ESI-Q-Orbitrap MS: Positive-ion mode *m*/*z* 665.19427 [M – H]$^-$ (calcd for $C_{27}H_{37}O_{19}$, 665.19236).

Mongophenoside A$_3$ (**9**): White powder; $[\alpha]_D^{25}$ –80.0 (*c* = 0.24, H_2O); IR ν_{max} (KBr) cm^{-1}: 3367, 2923, 2876, 1715, 1634, 1607, 1508, 1437, 1307, 1267, 1170, 1072, 1025; UV λ_{max} (H_2O) nm (log ε): 217 (4.02), 236 (3.85), 297 (4.09), 319 (4.05); ^1H (DMSO-d_6, 500 MHz) spectroscopic data: δ 7.19 (1H, br. s, H-2), 7.12 (1H, m, overlapped, H-5), 7.12 (1H, m, overlapped, H-6), 7.61 (1H, d, *J* = 16.0 Hz, H-7), 6.45 (1H, d, *J* = 16.0 Hz, H-8), 4.80 (1H, d, *J* = 7.5 Hz, H-1'), 3.31 (1H, m, overlapped, H-2'), 3.30 (1H, m, overlapped, H-3'), 3.17 (1H, dd, *J* = 8.0, 9.0 Hz, H-4'), 3.37 (1H, m, H-5'), [3.48 (1H, m, overlapped), 3.72 (1H, br. d, *ca.* *J* = 11 Hz), H_2-6'], 5.57 (1H, d, *J* = 7.0 Hz, H-1''), 3.49 (1H, m, overlapped, H-2''), 3.50 (1H, m, overlapped, H-3''), 3.23 (1H, dd, *J* = 8.0, 8.0 Hz, H-4''), 3.29 (1H, m, H-5''), [3.46 (1H, m, overlapped), 3.66 (1H, br. d, *ca.* *J* = 11 Hz), H_2-6''], 4.43 (1H, d, *J* = 7.5 Hz, H-1'''), 2.95 (1H, dd, *J* = 7.5, 8.0 Hz, H-2'''), 3.17 (1H, dd, *J* = 8.0, 9.0 Hz, H-3'''), 3.08 (1H, dd, *J* = 9.0, 9.0 Hz, H-4'''), 3.07 (1H, m, H-5'''), [3.33 (1H, m, overlapped), 3.46 (1H, m, overlapped), H_2-6''']; ^{13}C NMR (DMSO-d_6, 125 MHz) spectroscopic data, see Table 2. ESI-Q-Orbitrap MS: Positive-ion mode *m*/*z* 665.19452 [M – H]$^-$ (calcd for $C_{27}H_{37}O_{19}$, 665.19236).

Mongophenoside B (**10**): White powder; $[\alpha]_D^{25}$ +8.0 (*c* = 0.45, MeOH); IR ν_{max} (KBr) cm^{-1}: 3344, 2920, 2850, 1707, 1681, 1647, 1600, 1517, 1457, 1428, 1380, 1270, 1162, 1129, 1079, 1033; UV λ_{max} (MeOH) nm (log ε): 232 (3.57), 293 (3.87), 323 (3.61); ^1H (CD_3OD, 500 MHz) spectroscopic data: δ 7.17 (1H, br. s, H-2), 6.80 (1H, d, *J* = 8.0 Hz, H-5), 7.06 (1H, br. d, *ca.* *J* = 8 Hz, H-6), 7.60 (1H, d, *J* = 16.0 Hz, H-7), 6.34 (1H, d, *J* = 16.0 Hz, H-8), 3.88 (3H, s, 3-OCH_3), 4.48 (1H, d, *J* = 8.0 Hz, H-1'), 3.13 (1H, dd, *J* = 8.0, 8.5 Hz, H-2'), 3.34 (1H, m, H-3'), 3.29 (1H, m, overlapped, H-4'), 3.28 (1H, m, overlapped, H-5'), [3.68 (1H, dd, *J* = 6.5, 11.0 Hz), 3.78 (1H, br. d, *ca.* *J* = 11 Hz), H_2-6'], 5.10 (1H, d, *J* = 3.5 Hz, H-1''), 3.35 (1H, dd, *J* = 3.5, 9.0 Hz, H-2''), 3.67 (1H, dd, *J* = 9.0, 9.0 Hz, H-3''), 3.28 (1H, m, overlapped, H-4''), 3.78 (1H, m, H-5''), [3.64 (1H, dd, *J* = 5.5, 12.0 Hz), 3.84 (1H, br. d, *ca.* *J* = 12 Hz), H_2-6'']; ^{13}C NMR (CD_3OD, 125 MHz) spectroscopic data, see Table 2. ESI-Q-Orbitrap MS: Negative-ion mode *m*/*z* 517.15668 [M – H]$^-$ (calcd for $C_{22}H_{29}O_{14}$, 517.15518).

Table S1. The qualitative analysis of *A. mongolicum* extracts by UPLC-ESI-Q-Orbitrap MS

No.	Compounds	t_R (min)	Formula	Theoretical	Measured	Error (ppm)	Identification or Main fragment ions
1'	Mongophenoside A3 (**9**)*	0.91	$C_{27}H_{37}O_{19}^-$	665.19236	665.19293	0.86	179.03409 (6052), 161.02336 (100.00)
2'	Mongophenoside A2 (**8**)*	1.18	$C_{27}H_{37}O_{19}^-$	665.19236	665.19281	0.68	179.034094 (5.54), 161.02328 (100.00)
3'	Mongoflavonoside B2 (**6**)*	1.31	$C_{33}H_{37}O_{23}^-$	801.17201	801.17407	2.57	801.17407 (2.75), 639.11975 (6.40), 625.13953 (19.15), 463.08783 (34.27), 301.03452 (38.33), 300.02676 (60.70), 299.01941 (100.00), 271.02451 (66.02), 243.02882 (6.66), 178.99776 (6.96), 151.00258 (12.48)
4'	Mongoflavonoside A1 (**1**)*	1.49	$C_{33}H_{37}O_{22}^-$	785.17710	785.17450	–3.31	785.17450 (2.07), 609.14539 (30.18), 446.08475 (53.44), 285.03986 (11.47), 284.03098 (16.90), 283.02444 (100.00), 255.02945 (44.94), 151.00233 (3.44)
5'	Mongoflavonoside A3 (**3**)*	1.54	$C_{39}H_{47}O_{27}^-$	947.22992	947.22919	–0.77	947.22919 (11.26), 785.17670 (4.95), 771.19708 (6.23), 609.14569 (35.73), 447.09320 (12.16), 285.03998 (100.00), 284.03207 (45.91), 283.02451 (47.57), 255.02943 (48.25), 227.03432 (12.36), 151.00233 (11.75)
6'	Mongoflavonoside A4 (**4**)*	1.64	$C_{39}H_{47}O_{26}^-$	931.23501	931.23627	1.35	931.23627 (10.28), 593.15100 (20.36), 447.09332 (5.40), 285.03995 (100.00), 284.03244 (17.92), 283.02509 (1.76), 255.02936 (41.82), 227.03427 (26.47), 151.00247 (5.20)
7'	Mongophenoside A1 (**7**)*	1.71	$C_{21}H_{27}O_{14}^-$	503.13953	503.14072	2.36	503.14072 (1.18), 383.07678 (2.56), 179.03387 (3.90), 161.02324 (100.00), 133.02817 (23.43)
8'	Quercetin-3-*O*-*β*-D-glucopyranosyl(1→2)-*β*-D-glucopyranosyl-7-*O*-*β*-D-glucuronide (**32**)	1.86	$C_{33}H_{37}O_{23}^-$	801.17201	801.17462	3.25	801.17407 (6.71), 625.13953 (22.68), 301.03439 (49.39), 300.02676 (100.00), 299.01941 (3.14), 271.02451 (49.35), 255.02969 (23.83), 243.02882 (8.92), 178.99776 (7.67), 151.00258 (16.70)
9'	Kaempferol-3,7,4'-tri-*O*-*β*-glucoside (**18**)*	1.86	$C_{33}H_{39}O_{21}^-$	771.19783	771.19672	–1.45	609.14575 (1.50), 447.09219 (2.64), 285.04025 (100.00), 284.03241 (44.67), 283.02466 (53.29), 255.02939 (44.10), 227.03418 (12.79), 178.99773 (7.67), 151.00241 (16.70)

10'	Quercetin-3,7,4'-tri-*O*-glucoside (**26**)*	2.20	$C_{33}H_{39}O_{22}^{-}$	787.19275	787.19312	0.47	625.13782 (6.90), 462.08038 (37.57), 301.03513 (100.00), 300.02722 (73.85), 299.01950 (92.50), 284.02841 (2.52), 283.02460 (3.07), 178.99760 (4.33), 151.00233 (8.33)
11'	Quercetin-3-*O*-*β*-D-glucopyranosyl(1→6)-*β*-D-glucopyranosyl-7-*O*-*β*-D-glucuronide (**33**)	2.46	$C_{33}H_{37}O_{23}^{-}$	801.17201	801.17389	2.34	801.17407 (6.03), 625.13953 (32.76), 301.03439 (99.43), 300.02676 (100.00), 299.01941 (4.10), 271.02451 (57.86), 255.02969 (20.62), 243.02882 (11.97), 178.99776 (11.80), 151.00258 (30.26)
12'	*trans*-*p*-Hydroxycinnamate sophorose (**28**)*	2.67	$C_{21}H_{27}O_{13}^{-}$	487.14462	487.14279	–3.75	367.10297 (6.79), 163.03888 (7.99), 145.02818 (100.00), 117.03317 (18.64)
13'	Quercetin-3-*O*-*β*-D-rutinoside-7-*O*-*β*-D-glucuronide (**25**)*	2.67	$C_{33}H_{37}O_{22}^{-}$	785.17710	785.18005	–4.24	785.18005 (5.22), 609.14569 (22.70), 301.03442 (41.66), 300.02707 (100.00), 299.01984 (2.11), 271.02451 (51.58), 255.02939 (23.22), 243.02937 (10.05), 178.99771 (6.07), 151.00244 (13.36)
14'	Quercetin-3-*O*-*β*-D-glucopyranosyl(1→4)-*β*-D-glucopyranosyl-7-*O*-*β*-D-glucuronide (**34**)	2.67	$C_{33}H_{37}O_{23}^{-}$	801.17201	801.17200	–0.02	801.17407 (6.66), 625.13953 (25.19), 301.03439 (39.74), 300.02676 (100.00), 299.01941 (2.19), 271.02451 (61.76), 255.02969 (16.96), 243.02882 (11.90), 178.99776 (5.94), 151.00258 (14.77)
15'	*cis*-*p*-Hydroxycinnamate sophorose (**35**)	2.96	$C_{21}H_{27}O_{13}^{-}$	487.14462	487.14313	–3.05	367.10297 (6.79), 163.03888 (7.99), 145.02818 (100.00), 117.03317 (18.64)
16'	Quercetin-3,4'-di-*O*-*β*-D-glucopyranoside (**23**)*	3.00	$C_{27}H_{29}O_{17}^{-}$	625.13993	625.14056	1.02	625.14056 (26.55), 463.08789 (17.92), 301.03491 (42.02), 300.02539 (17.74), 299.01932 (100.00), 271.02441 (60.41)
17'	Mongophenoside B (**10**)*	3.37	$C_{22}H_{29}O_{4}^{-}$	517.15518	517.15588	1.35	397.11356 (9.50), 193.04959 (16.56), 175.03899 (100.00), 160.01540 (71.94)
18'	Tuberonoid A (**29**)*	3.60	$C_{22}H_{29}O_{14}^{-}$	517.15518	517.15564	0.89	397.11356 (9.50), 193.04959 (16.56), 175.03899 (100.00), 160.01540 (71.94)
19'	*trans*-Caffeic acid (**30**)*	3.73	$C_{9}H_{7}O_{4}^{-}$	179.03389	179.03395	0.36	179.03389 (18.46), 161.02324 (100.00), 135.04378 (100.00)
20'	Benzyl-*O*-*β*-D-glucopyranoside (**31**)*	3.75	$C_{14}H_{19}O_{8}^{-}$	315.10744	315.10828	2.65	268.93088 (100.00)
21'	Kaempferol-3-*O*-rutinoside-7-*O*-glucuronide (**15**)*	4.07	$C_{33}H_{37}O_{21}^{-}$	769.18218	769.18140	–1.02	593.15063 (39.19), 285.03964 (100.00), 284.03210 (81.62), 255.02931 (72.41), 227.03410 (45.87), 178.

							99751 (0.81), 151.00253 (2.45)
22'	Kaempferol-3-*O*-*β*-D-glucopyranosyl(1→2)-*O*-*β*-D-glucopyranosyl-4'-*O*-*β*-D-glucopyranoside (**36**)	4.09	$C_{33}H_{39}O_{21}^{-}$	771.19783	771.19867	1.08	285.03976 (100.00), 284.03244 (55.81), 283.02512 (0.12), 255.02921 (2.29), 227.03360 (0.81), 151.00240 (2.66)
23'	Kaempferol-3-*O*-gentiobioside-4'-*O*-glucopyranoside (**17**)*	4.26	$C_{33}H_{39}O_{21}^{-}$	771.19783	771.19714	–0.90	609.14596 (43.20), 285.04008 (100.00), 284.03192 (44.67), 283.02454 (53.41), 255.02942 (61.40), 227.03374 (15.94), 178. 99770 (0.43), 151.00259 (2.45)
24'	Kaempferol-3-rutinoside-4'-glucopyranoside (**16**)*	4.32	$C_{33}H_{39}O_{20}^{-}$	755.20292	755.20386	1.25	755.20496 (2.30), 593.15015 (41.59), 446.08542 (5.99), 285.03998 (100.00), 284.03241 (36.55), 283.02409 (54.25), 255.02953 (52.49), 227.03419 (12.96), 178. 99770 (0.61), 151.00249 (1.98)
25'	Rutin (**22**)*	4.40	$C_{27}H_{29}O_{16}^{-}$	609.14501	609.14551	0.82	609.14551 (43.20), 301.03394 (48.08), 300.02719 (11.59), 271.02440 (100.00), 255.02942 (61.40), 243.02915 (15.94), 151.00246 (2.29)
26'	Quercetin 3-*O*-(6''-*O*-*α*-L-rhamnopyranosyl)-*β*-D-glucopyranoside-7-*O*-*β*-D-glucopyranoside (**24**)*	5.36	$C_{33}H_{39}O_{20}^{-}$	755.20292	755.20306	0.19	593.15002 (26.16), 285.03995 (100.00), 284.03238 (33.45), 283.02466 (6.73), 255.02939 (44.10), 227.03418 (12.79)
27'	Mongoflavonoside A$_2$ (**2**)*	5.50	$C_{33}H_{39}O_{21}^{-}$	771.19783	771.19849	0.85	609.14596 (43.20), 285.04008 (100.00), 284.03241 (31.64), 283.02454 (53.41), 255.02942 (61.40)
28'	Isorhamnetin-3-*O*-rutinosyl-7-*O*-*β*-D-glucuronide (**37**)	6.62	$C_{34}H_{39}O_{22}^{-}$	799.19275	799.19391	1.45	799.19391 (5.36), 623.15869 (27.48), 315.05048 (100.00), 300.02713 (39.15), 299.01956 (4.47), 271.02469 (43.73), 243.02922 (32.91)
29'	Quercetin-3-*O*-*β*-D-glucopyranosyl(1→4)-*β*-D-glucopyranoside (**21**)*	6.76	$C_{27}H_{29}O_{17}^{-}$	625.13993	625.14117	1.99	625.14056 (30.89), 301.03494 (38.25), 300.02719 (100.00), 271.02463 (63.94), 255.02899 (30.43)
30'	Isoquercetin (**19**)*	6.95	$C_{21}H_{19}O_{12}^{-}$	463.08710	463.08771	1.31	463.08771 (44.71), 301.03480 (100.00), 300.03480 (77.40), 271.02444 (45.93), 255.02951 (21.68), 243.02890 (10.77)
31'	Kaemperol-3-*O*-*β*-D-gluco	7.50	$C_{33}H_{39}O_{20}^{-}$	755.20292	755.20349	0.76	755.20349 (9.40), 285.03986 (100.00), 284.03207

	pyranosyl(1→4)[*α*-L-rhamanopyranosyl(1→6)]-*β*-D-glucopyranoside (**14**)*						(54.63), 255.02940 (51.57), 227.03423 (28.78)
32'	Mongoflavonoside B_1 (**5**)*	7.76	$C_{29}H_{31}O_{18}^-$	667.15049	667.15118	1.03	301.03409 (36.28), 300.02725 (100.00), 299.01929 (1.50), 285.04056 (10.07), 284.03271 (4.21), 255.02946 (2.43), 178. 99770 (3.95), 151.00247 (3.86)
33'	Kaempferol-3-*O*-*β*-D-rutinoside (**13**)*	7.82	$C_{27}H_{30}O_{15}^-$	593.15010	593.15076	1.12	593.15076 (38.41), 285.03972 (100.00), 284.03198 (76.48), 255.02914 (62.67), 227.03400 (41.34)
34'	Kaempferol-3-*O*-*β*-D-glucopyranosyl(1→4)-*β*-D-glucopyranoside (**12**)*	7.87	$C_{27}H_{29}O_{16}^-$	609.14501	609.14581	1.31	609.14551 (19.16), 285.03986 (100.00), 284.03214 (52.77), 255.02939 (50.42), 227.0423 (30.16)
35'	Kaempferol-3-*O*-*β*-D-glucopyranoside (**11**)*	8.09	$C_{21}H_{19}O_{11}^-$	447.09218	447.09305	1.93	447.09305 (76.03), 285.03949 (44.45), 284.03204 (85.22), 255.02936 (100.00), 227.0421 (77.78)
36'	Quercetin-3-*O*-(6''-*O*-acetyl)-*β*-D-glucopyranoside (**20**)*	8.57	$C_{23}H_{21}O_{13}^-$	505.09767	505.09839	1.43	505.09839 (44.85), 301.03333 (33.67), 300.02689 (100.00), 271.02432 (78.42), 255.02922 (29.76), 243.02908 (16.41)
37'	Isorhamnetin 3-*O*-*β*-D-glucopyranoside (**27**)*	9.15	$C_{22}H_{21}O_{12}^-$	477.10275	477.09286	1.50	447.09305 (53.28), 299.01935 (12.83), 285.03915 (31.09), 284.03204 (100.00)

*The compounds unambiguously identified with the reference standards comparison; Glc: *β*-D-glucopyranosyl; Glu: *β*-D-glucuronyl; Rha: *α*-L-rhamnopyranosyl; Rut: rutinnosyl.
